# Genome assembly of an Australian native grass species reveals a recent whole-genome duplication and biased gene retention of genes involved in stress response

**DOI:** 10.1093/gigascience/giad034

**Published:** 2023-05-12

**Authors:** Nissanka P De Silva, Christopher Lee, Paul Battlay, A Fournier-Level, Joslin L Moore, Kathryn A Hodgins

**Affiliations:** School of Biological Sciences, Monash University, Clayton, 3800 Victoria, Australia; School of Biological Sciences, Monash University, Clayton, 3800 Victoria, Australia; School of Biological Sciences, Monash University, Clayton, 3800 Victoria, Australia; School of BioSciences, University of Melbourne, Melbourne, 3010 Victoria, Australia; School of Biological Sciences, Monash University, Clayton, 3800 Victoria, Australia; Arthur Rylah Institute for Environment Research, Heidelberg, 3084 Victoria, Australia; School of Biological Sciences, Monash University, Clayton, 3800 Victoria, Australia

**Keywords:** genome assembly, annotation, paleo-allopolyploidization, whole-genome duplication (WGD), biased fractionation

## Abstract

**Background:**

The adaptive significance of polyploidy has been extensively debated, and chromosome-level genome assemblies of polyploids can provide insight into this. The Australian grass *Bothriochloa decipiens* belongs to the BCD clade, a group with a complex history of hybridization and polyploid. This is the first genome assembly and annotation of a species that belongs to this fascinating yet complex group.

**Findings:**

Using Illumina short reads, 10X Genomics linked reads, and Hi-C sequencing data, we assembled a highly contiguous genome of *B. decipiens*, with a total length of 1,218.22 Mb and scaffold N50 of 42.637 Mb. Comparative analysis revealed that the species experienced a relatively recent whole-genome duplication. We clustered the 20 major scaffolds, representing the 20 chromosomes, into the 2 subgenomes of the parental species using unique repeat signatures. We found evidence of biased fractionation and differences in the activity of transposable elements between the subgenomes prior to hybridization. Duplicates were enriched for genes involved in transcription and response to external stimuli, supporting a biased retention of duplicated genes following whole-genome duplication.

**Conclusions:**

Our results support the hypotheses of a biased retention of duplicated genes following polyploidy and point to differences in repeat activity associated with subgenome dominance. *B. decipiens* is a widespread species with the ability to establish across many soil types, making it a prime candidate for climate change– resilient ecological restoration of Australian grasslands. This reference genome is a valuable resource for future population genomic research on Australian grasses.

## Background

Whole-genome duplication (WGD), or polyploidy, occurs via the doubling of chromosomal material either involving 1 species (autopolyploidy) or via hybridization of 2 species (allopolyploidy). The polyploid origin of many plant species has long been recognized [1, 2], but while polyploidy is commonly observed in angiosperms, its evolutionary importance has been controversial. Some studies support the hypothesis that polyploidy can drive rapid adaptive evolution [3, 4], while others have argued that polyploidy has played a minimal role in evolution and contributed little to adaptation [5]. However, there is growing evidence that ancestral WGD events caused key changes in major angiosperm clades, supporting their successful diversification [6].

After a WGD event, various molecular changes occur to restore the diploid state (diploidization; [7–11] via genome rearrangement, gene loss, and epigenetic change [7, 12]. Genes that encode DNA repair mechanisms and organelle functions tend to revert to single-copy status following WGD events [13, 14]. However, other duplicated genes may be retained [15]. Several hypotheses explain the patterns of duplicated gene retention and their evolutionary fate. For instance, the gene balance hypothesis states that genes coding for products that are dose sensitive are protected from fractionation because, if fractionated, the stoichiometry of the products and other gene products that they interact with will be affected and may bring about negative or lethal effects to the organism [16]. These principles are thought to apply to genes responsible for controlling functions related to gene regulation such as transcription factors or kinases acting as hubs with the potential to control entire gene networks [17, 18].

Functionally distinct subgenomes may also be retained after WGD. Biased fractionation through the preferential loss of the duplicated genes from the same subgenome has been observed in many polyploids [19–22]. Gene expression also tends to be biased between the subgenomes with higher gene expression in the subgenome that experiences less gene loss [19, 20, 23, 24]. The less fractionated and more transcriptionally active subgenome is referred to as the dominant subgenome, and this asymmetry frequently occurs in hybrids with divergent parental genomes [25, 26].

Poaceae (grasses) is the most successful plant family in terms of occurrence, ecological dominance, and species richness [27], and approximately 80% of species in this family are polyploid, and all grasses are derived from a polyploid event called rho [28]. Our study species, *Bothriochloa decipiens* (NCBI:txid883134), belongs to the tribe Andropogoneae (subfamily Panicoideae). This tribe contains many ecologically and economically important species, and independent allopolyploidization events have been exceptionally frequent in this group [29]. *B. decipiens* is part of a cosmopolitan grass genus [30] closely related to *Capillipedium* and *Dichanthium* (together referred to as BCD). These 3 genera have the ability to interbreed despite their morphological differences; the term *compilospecies* was coined to describe this type of hybrid species complex [31, 32], and *B. decipiens* may be a donor species to this compilospecies complex [33].

Here we report a chromosome-level genome assembly, annotation, and comparative analysis of a species from the BCD clade, *B. decipiens*. This is the first genome assembly and annotation of a species that belongs to this fascinating yet complex group. Our highly contiguous *B. decipiens* genome assembly showed clear evidence of recent paleo-polyploidy. Using repeat signatures diverged between putative homoeologous chromosomes, we were able to organize chromosomes into subgenomes, allowing estimation of the timing of the speciation event prior to the most recent allopolyploidization event in this species. We further describe signatures of biased fractionation between subgenomes, as well as biases in the functions of genes retained as duplicated or single copy. This genome reference will act as an important resource for population genomic analysis of the BCD clade and will aid our understanding of the rich history of allopolyploidy in this group and its evolutionary significance.

## Analyses

### Genome size estimation, genome assembly, and transcriptome assembly

Using flow cytometry (FCM) (see Methods), the haploid genome size of the accession COB1-7 was estimated to be 1.25 Gb. The genome was assembled combining assemblies from linked read sequencing (10X) with HiRise scaffolding using Chicago and Hi-C libraries (Dovetail Genomics; [34] Table 1). As *B. decipiens* is known to have a haploid chromosome number *n* = 20 [35, 36] and the final assembly had a L90 = 20 (Table 1), we assumed that these scaffolds represented the 20 haploid chromosomes of *B. decipiens*.

**Table 1:** Statistics of the *Bothriochloa decipiens* genome assembly

Assembly statistics	10X	10X + Chicago	10X + Chicago + Hi-C
Contig L50/N50 (number/size)	19,068 scaffolds/80.27 kb (kilo base pairs)	19,068 scaffolds/80.27 kb	16,083 scaffolds/80.20 kb
Scaffold L50/N50 (number/size)	125 scaffolds/2.733 Mb (mega base pairs)	116 scaffolds/3.080 Mb	10 scaffolds/53.95 Mb
Scaffold L90/N90 (number/size)	1,808 scaffolds/0.023 Mb	616 scaffolds/0.090 Mb	20 scaffolds/42.637 Mb
Largest scaffold size	14.847 Mb	14.607 Mb	95.095 Mb
Total number of scaffolds	25,759	19,068	15,895
Total genome size	1,217.63 Mb	1,218.36 Mb	1,218.22 Mb
% Gaps	6.78	6.84	6.86
BUSCO**^†^**(*n*)	39:200:13:3	43:200:9:3	45:200:8:2
BUSCO**^†^** (%)	15:78:5:1	16:78:3:1	17:78:3:0.8

† Number of BUSCO genes found in the assembly using the eukaryota odb9 dataset. Genes are split into 4 categories: complete and single copy, complete and duplicated, fragmented, and missing and reported, respectively.

RNA sequencing (RNA-seq) data from 2 tissues (leaf and stem) were used to assemble the transcriptome of *B. decipiens* using Trinity v.2.8.5 (RRID:SCR_013048) [37]. The final transcriptome assembly contained 197,655 transcripts, with 104,784 Trinity annotated genes, with an average length of 1,062 bp and an N50 length of 1,677.

### Genome annotation, functional annotation, quality validation, and repeat identification

**Figure 1:**
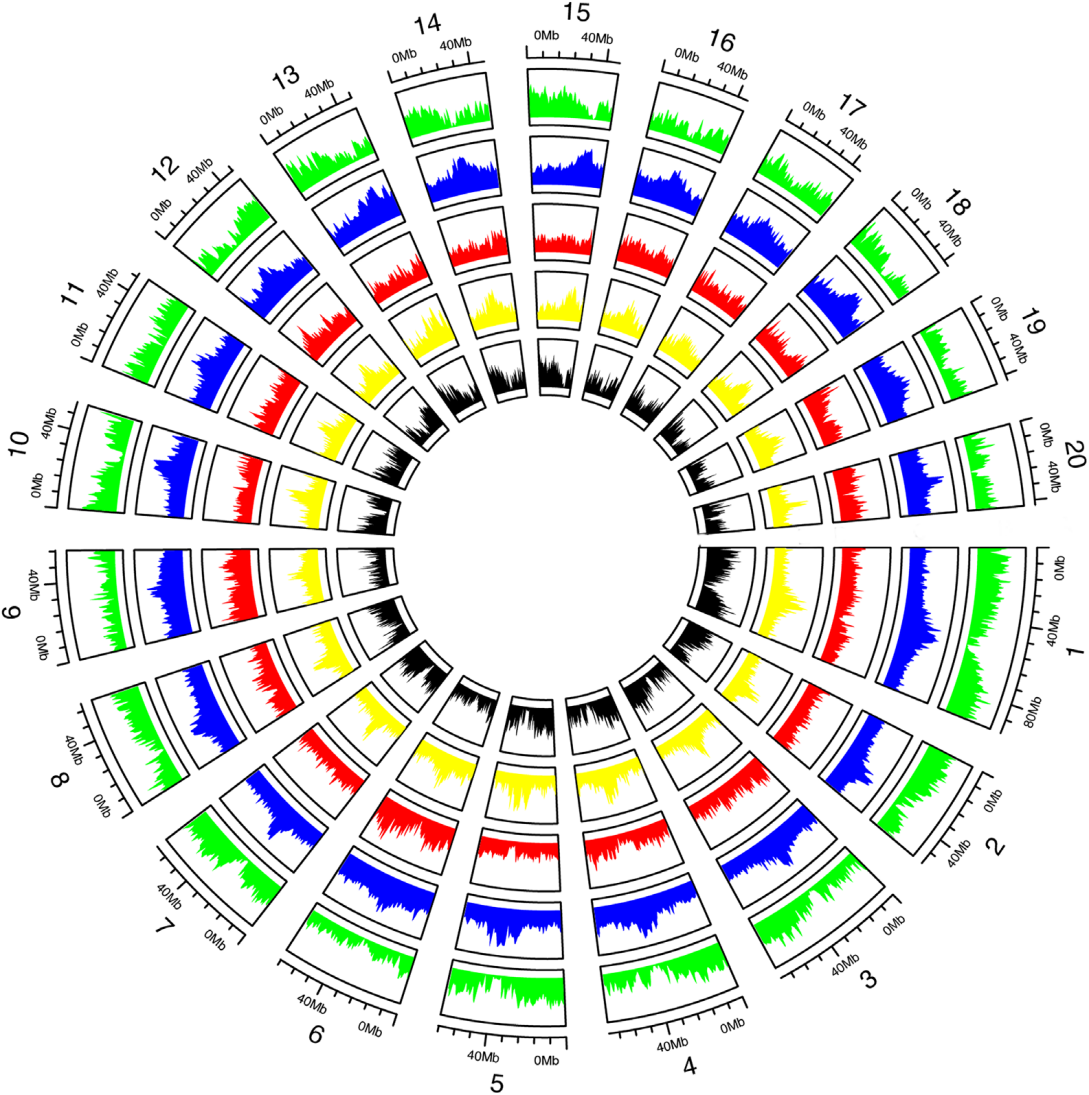
The *Bothriochloa decipiens* genome landscape. Location across the 20 chromosomes and distribution, in 1-Mb windows of gene density (green track), repeat density (blue track), DNA-TE density (red track), LTR-TE density (yellow track), and GC (guanine-cytosine) content (black track).

We identified 60,652 putative protein coding genes (Supplementary Table S1). This was done by running iterative runs of the genome annotation pipeline MAKER v.3.01.03 (RRID:SCR_005309) [38] and training gene predictions using AUGUSTUS v.3.3.3 (RRID:SCR_008417) [39] and SNAP v.2013-11-29 [40]. Functional annotations for the predicted genes were done by searching against several databases (see Methods) (Table 2). We identified 94.1% of the core eukaryotic genes among our annotated genes, 22.7% being single copy, 71.4% duplicated, and 2.4% fragmented compared to BUSCO markers present in the library “eukaryota_odb10.2020-09.10.” Of the total genome assembly, 57.88% corresponded to repetitive elements (Table 3). Repeated elements annotation was done using EDTA v.2.0.0 (RRID:SCR_022063) [41] (see Methods). The majority of the repeated elements were long terminal repeats (LTRs) (39.51%). We also observed quite a large proportion of helitrons (10.35%) being present in the genome. A detailed summary of the transposable element (TE) annotations is provided in Table 3. Analysis of the *B. decipiens* repeat and gene density across each chromosome reveals that gene density was low toward the center of each scaffold, where repeat density was high (Fig. 1).

**Table 2:** Summary of functional annotation for protein coding genes

Database	Number of gene models with annotation	Percentage of gene models with annotation
UniProtKB/Swiss-Prot	42,417	69.93
Tair10	49,099	80.95
Pfam	43,444	71.62
KEGG orthology	17,465	28.79
KEGG orthology—EC (Enzyme Commission) number annotations	8302	13.68

**Table 3:** Summary of length of genome sequence repeat-masked by repeat element class

Major repeat class	Superfamily	Mbp present	% of genome
Long terminal repeats (LTRs)	Copia	76.29	6.72
	Gypsy	315.39	27.80
	Unknown	56.62	4.99
Terminal inverted repeats (TIRs)	CACTA	28.09	2.48
	Mutator	20.17	1.78
	PIF Harbinger	8.19	0.72
	Tc1 Mariner	12.62	1.11
	hAT	6.60	0.58
	polinton	0.04	0.00
Non-LTR	LINE element	0.62	0.05
Non-TIR	helitron	117.49	10.35
Repeat region		14.59	1.29

### Synteny and whole-genome duplication

**Figure 2:**
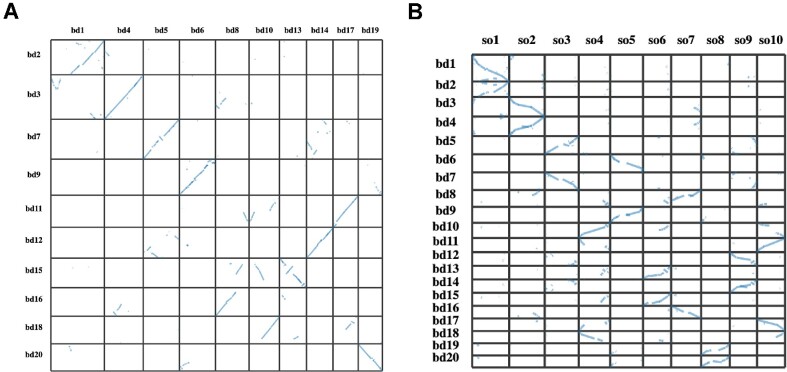
(A) The syntenic relationship between the pairs of homoeologous chromosomes in *B. decipiens*. (B) The syntenic relationship between the *B. decipiens* and *S. bicolor* orthologous genes. Each *S. bicolor* chromosome (so) shares synteny with a pair of *B. decipiens* chromosomes (bd), suggesting that the allotetraploid *B. decipiens* genome was formed by the hybridization of 2 *Sorghum*-like ancestors.

In order to determine if *B. decipiens* had undergone a WGD, a reciprocal BLASTP (RRID:SCR_001010) [42] was conducted using *B. decipiens* protein sequences as the query against themselves, and homoeologous scaffolds were identified using the collinear blocks obtained via MCScanX (RRID:SCR_022067) [43]. This was also corroborated by the alignment against themselves of the 20 largest scaffolds (>40 Mb, representing 1.11 Gb of the 1.22-Gb genome) using Minimap2 v.2.1.8 (RRID:SCR_018550) [44] (Supplementary Fig. S1). Of these 20 scaffolds, 10 pairs (with more than 50% matching across both scaffolds) were identified as homoeologous (Supplementary Fig. S1). Similarly, we identified collinear blocks between the 2 putative subgenomes of *B. decipiens* and *Sorghum bicolor* (the closest diploid relative with a high-quality genome assembly) by conducting a reciprocal BLASTP [42] comparing protein sequences from each species using MCScanX [43]. We identified 33,146 *B. decipiens* genes that were orthologous to 19,611 *S. bicolor* genes across syntenic blocks. A relatively recent paleo-polyploidization event was evident as each chromosome from *S. bicolor* almost completely aligned to a pair of *B. decipiens* scaffolds as seen in the dot plot in Fig. 2B. Further, these pairs of *B. decipiens* scaffolds show large syntenic blocks of duplicated genes, as seen in the dot plot in Fig. 2A. There was also some evidence of rearrangements between the subgenomes: for example, a translocation from scaffold 18 (homoeologous to scaffold 10) to scaffold 8, which appears to have regions from both subgenomes as a result (Fig. 2). Therefore, this translocation likely occurred after the allopolyploidization event. Translocations are apparent in the *B. decipiens* genome alignment against itself (Supplementary Fig. S1) and also in the syntenic relationship between the *B. decipiens* chromosomes when all 20 of them are aligned against themselves (Supplementary Fig. S2). Other structural changes can be observed, including several inversions, clearly identifiable on scaffold 13 when compared to scaffold 15 or to chromosome 6 in *S. bicolor* (Fig. 2B).

### Genomic exchange across subgenomes and homoeologous pairs/chromosomes

**Figure 3:**
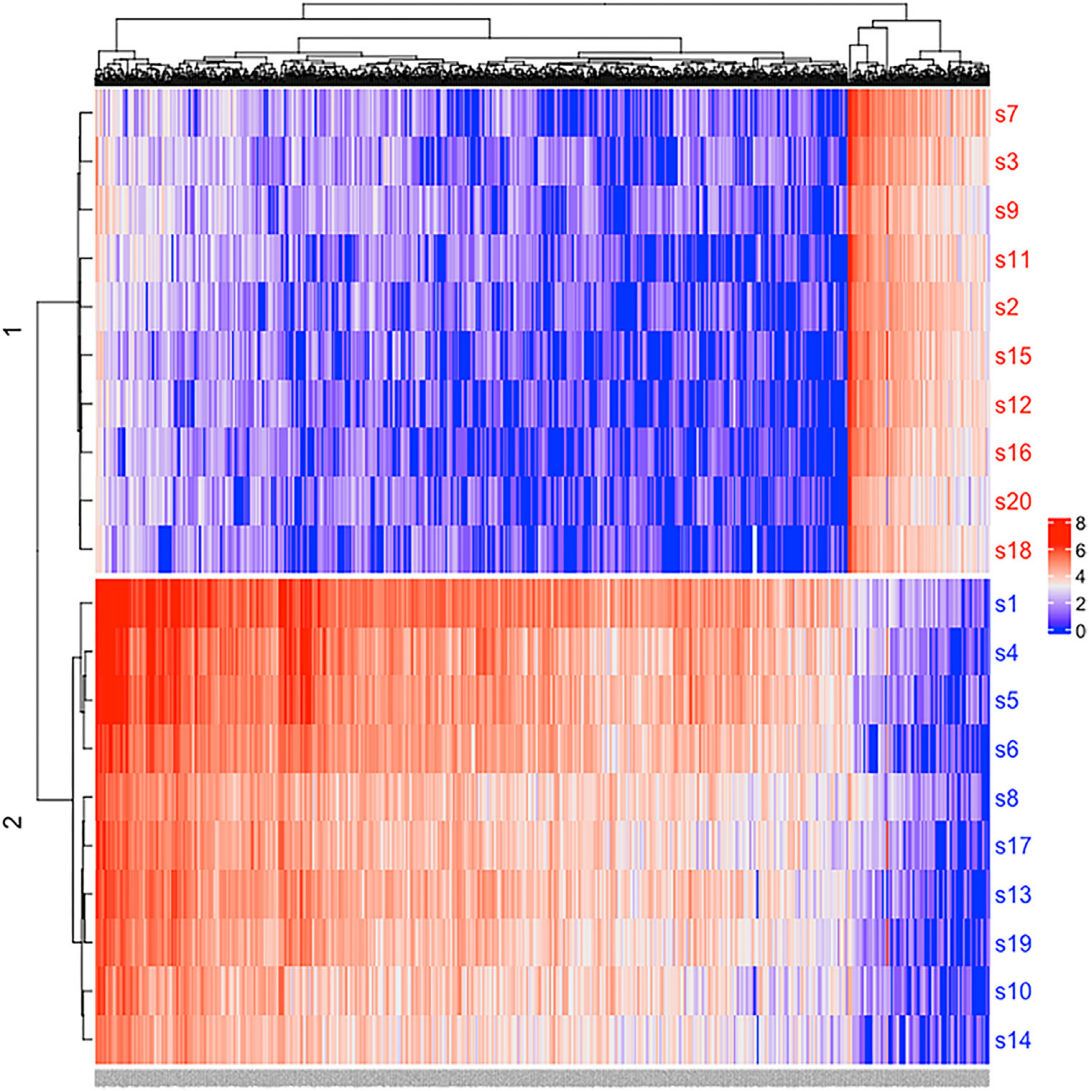
The differentiation of homoeologous pairs of chromosomes into subgenome A (blue) and subgenome B (red) based on the hierarchical clustering of Euclidean distances among scaffolds using counts of 13-mers.

The difference in the distribution of repetitive elements between pairs of homoeologous chromosomes can provide evidence of subgenome ancestry [45]. Diagnostic repeat signatures are found on one of each homoeologous pair of chromosomes, and they represent the remains of mobile elements with different activity in the diploid ancestors before the merging of the 2 genomes [45]. As there are no genomic data from any close diploid relative that does not share the most recent allopolyploidization event, we clustered the putative homoeologous chromosomes based on repeat abundance using *k*-mer distributions. We partitioned the *B. decipiens* genome into subgenomes A and B by modifying the methods described in [46] (see Methods). We found 919 13-mers (13-bp sequences) occurring at least 100 times across the whole genome and also were at least 3-fold enriched in one of the homoeologous chromosomes relative to the other. Based on the consistent enrichment for these 919 13-mers along the putative homoeologous chromosomes, each scaffold of a pair was assigned to a subgenome (Fig. 3). The A group was defined based on the excessive abundance of 773 13-mers and the B group based on the abundance of the other 146 13-mers (Fig. 3). We then computed the densities of A- and B-preferred 13-mers across the scaffolds (Supplementary Fig. S3A, B) and identified potential homoeologous exchange between subgenomes. Scaffold 8 from the B subgenome had a high density of subgenome A–preferred *k*-mers at one end of the scaffold (Supplementary Fig. S3B), consistent with the observation of a translocation from the dot plots (Fig. 2). We then tested for homoeologous exchange across the subgenomes using a hidden Markov model (HMM) implemented in the R/HMM package [47]. We did this to determine if there was evidence for exchange of chromosomal regions between the subgenomes. In particular, we were interested in evidence of reciprocal homoeologous exchange as this would not be apparent in the dot plots (e.g., Fig. 2). However, we did not find evidence of reciprocal homoeologous exchange. For each chromosome clustered into the A or B subgenome, only rare instances of assignment to the alternate subgenome by the HMM occurred (except scaffold 8 mentioned above). Specifically, alternative assignment occurred in 3 regions (scaffolds 9, 12, and 16), but these were regions with few A- and B-preferred *k*-mers, and therefore sampling error would be more likely to contribute to misassignment to subgenomes using the HMM-based approach. Also, these instances did not reflect reciprocal exchanges between the subgenomes. Impacts of these ambiguities in subgenome assignment were examined by including and excluding these regions in downstream analyses relying on subgenome assignment (i.e., biased fractionation).

We examined subgenome-enriched LTRs, as differences in LTR activity in parental species can help differentiate subgenomes and can be used to assess the timing of allopolyploidy. We identified LTR repeats that belonged to 9 LTR families that were at least 3 times more common in 1 subgenome. A- or B-preferred *k*-mers also overlapped with these repeated elements. There were 8 LTR subfamilies (Grande1_ZM_pol/Gypsy, RIRE2_pol/Gypsy, Copia-11_SB/Copia, Copia-73_Mad/Copia, Copia-13_SB/Copia, SZ7_pol/Gypsy, CRM/Gypsy, Atlantys_OS_polGypsy) identified in subgenome A and only 1 (Copia-9_SB/Copia) identified in subgenome B. Their genomic locations are shown in Supplementary Fig. S3C, D.

### The timeline of paleo-tetraploidy

**Figure 4:**
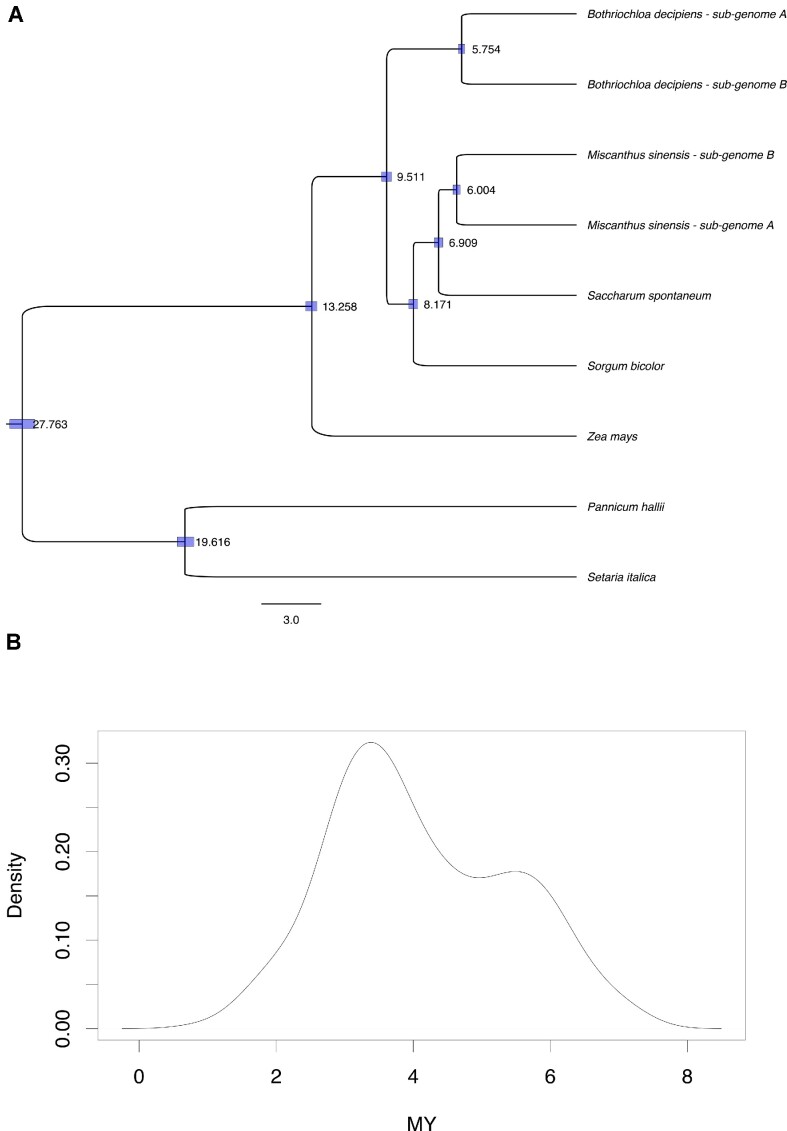
(A) Phylogenetic tree (MYA) of the Andropogoneae showing the time (MYA) of divergence between the diploid ancestors of the allotetraploid *B. decipiens*. (B) Density distribution of divergence in the time estimate for all LTR families with subgenome-specific expansion activity.

We used 1:1 orthologs identified using OrthoFinder v.2.3.8 (RRID:SCR_017118) [48] across members of the Andropogoneae tribe using *Panicum hallii* and *Setaria italica* as outgroups to identify the likely timing of the subgenome divergence in *B. decipiens* (see Methods; Fig. 4A). We found that the diploid progenitors of the allopolyploid ancestor of *B. decipiens* speciated approximately 5.8 million years ago (MYA). Our tree also dated the divergence of the progenitors of *M. sinensis* to around 6 MYA.

### Timing of subgenome-specific LTR expansion

The greater abundance of LTR subfamilies specific to 1 subgenome is a signal of mobile element activity unique to one of the diploid ancestors of the allopolyploid [45]. Therefore, dating the insertion events of the subgenome-specific LTRs (see Methods) can be used to provide a rough estimate of the timing of the hybridization event. We found that the subgenome-specific LTR activity began 2 to 3 MYA and peaked around 3.5 MYA (Fig. 4B). Activity declined around 6 MYA (Fig. 4B), and this coincides with the timing of the speciation event of the diploid ancestors prior to the allopolyploidy event inferred through the phylogenetic analysis (Fig. 4A). Overall, this analysis suggests that the diploid ancestors may have evolved independently for about 3 to 4 MYA before the hybridization event.

### Biases in gene retention between subgenomes

We analyzed the collinear blocks between *S. bicolor* and *B. decipiens* to assess differences in retention of duplicated genes between subgenomes. Subgenome-specific retention was inferred as the number of genes retained in a given subgenome divided by the number of inferred ancestral (i.e., preduplication) gene numbers. Collinear blocks obtained from the 2 independent scanning methods McScanX [43] and OrthoFinder v.2.3.8 [48] were used in 2 independent analyses to confirm patterns of fractionation (see Methods). We then used a 2-sided Fisher's exact test to determine if there was a significant difference in retention of genes between the subgenomes under the null hypothesis that gene loss between subgenomes was random. The percentage of genes retained was significantly higher in subgenome A compared to subgenome B (McScanX, Fisher's exact test, *P* = 2.2 × 10^−16^ and OrthoFinder, Fisher's exact test, *P* = 1.63 × 10^−10^ (Table 4).

**Table 4:** Subgenome-specific gene retention

Clustering method	Ancestral genes (retained + single)	Ancestral genes retained on A	Ancestral genes retained on B	Ancestral genes retained on A + B	Percent retained on A	Percent retained on B	Percent retained on A + B
McScanX	19,523	16,666	15,464	12,195	85	79	62
OrthoFinder	15,262	12,716	12,278	9,620	83	80	53

Differences in the function of duplicated genes retained after the WGD compared to those returning to single copy were tested through gene ontology enrichment analysis using the R/topGo package (RRID:SCR_014798) [49]. All single-copy genes in A or B, or duplicated genes in both A and B, were used as foreground genes and the remaining ancestral genes (retained duplicated or single copy) as background genes. Gene Ontology (GO) terms related to organelle functions, such as chloroplast organization, chloroplast RNA modification, and regulation of mitochondrion organization, were among the most significant terms associated with genes returning to single copy (Table 5A, Supplementary Fig. S4A). The most significantly enriched terms for genes maintained as duplicates were those relating to transcription, including positive and negative regulation of transcription, and terms related to external stress responses, such as response to water deprivation and salt stress (Table 5B, Supplementary Fig. S4B).

**Table 5:** Summary of the gene enrichment analysis. (A) Summary of the most significant GO terms (Fisher's exact test *P* ≤ 1 × 10^−3^) relating to RNA modification, transmembrane activity, and organelle functions among overrepresented genes in genes retained as single copies as reported by the gene enrichment analysis. (B) Summary of the most significant GO terms (Fisher's exact test *P* ≤ 1 × 10^−3^) relating to transcription and external stress response among overrepresented genes in genes retained as duplicates as reported by the gene enrichment analysis.

(A)					
GO ID	Term	Annotated	Significant	Expected	*P* value
GO:0009451	RNA modification	451	128	74	3.6 × 10^−12^
GO:0120009	Intermembrane lipid transfer	14	11	2.26	3.3 × 10^−7^
GO:0009658	Chloroplast organization	442	92	71.26	2.1 × 10^−4^
GO:0071712	Endoplasmic reticulum (ER)–associated misfolded protein catabolism	23	11	3.71	3.0 × 10^−4^
GO:1900865	Chloroplast RNA modification	53	22	8.55	3.3 × 10^−4^
GO:0010821	Regulation of mitochondrian organization	17	10	2.74	4.5 × 10^−4^
(B)					
GO:0045893	Positive regulation of transcription	962	550	455.35	7.4 × 10^−18^
GO:0045892	Negative regulation of transcription	593	333	280.69	7.0 × 10^−9^
GO:0006355	Regulation of transcription	3,176	1,759	1,503.31	1.9 × 10^−8^
GO:0006357	Regulation of transcription by RNA polymerase	836	456	395.71	6.5 × 10^−8^
GO:0009414	Response to water deprivation	963	460	455.82	1.7 × 10^−6^
GO:1901002	Positive regulation of response to salt stress	84	60	39.76	2.4 × 10^−6^
GO:0009788	Negative regulation of abscisic acid signaling pathway	107	70	50.65	4.6 × 10^−5^
GO:0009646	Response to absence of light	88	60	41.65	6.1 × 10^−5^
GO:0009409	Response to cold	689	371	326.13	3.8 × 10^−4^

### TE density between the subgenomes

TE density is known to be lower in genic regions for the dominant subgenome in some other neo- and paleopolyploids [25, 26, 50]; therefore, we analyzed the TE density in regions flanking genes to test if a difference in TE density exists between the 2 subgenomes and between single-copy and duplicated genes identified in the biased gene retention analysis. This is thought to reflect increased downregulation of genes owing to methylation of nearby TEs [51, 52]. The density of TEs with 5,000 bp of genes was not significantly different between the subgenomes upstream of the genes (Mann–Whitney *U* test, *P* = 3.3 × 10^−1^) (Table 6A) and also downstream of genes (Mann–Whitney *U* test, *P* = 1.4 × 10^−1^) (Table 6A). The TE density flanking single-copy and duplicated genes was also not significantly different for the upstream of genes (Mann–Whitney *U* test, *P* = 2.6 × 10^−1^) (Table 6B) or downstream of genes (Mann–Whitney *U* test, *P* = 4.5 × 10^−1^) (Table 6B).

**Table 6:** Summary of TE density in flanking regions of genes. (A) Genome-specific TE density flanking genes (within 5,000 bp for the start or end of the gene). (B) TE density distribution with relation to the location of genes near single-copy genes and genes retained as duplicated (within 5,000 bp of the start or end of the gene).

(A)		
Subgenome	Mean TE density to the upstream of the genes	Mean TE density to the downstream of the genes
A	0.374 ± 0.01	0.375 ± 0.01
B	0.382 ± 0.01	0.383 ± 0.01
(B)		
Gene type		
Single copy	0.362 ± 0.008	0.357 ± 0.007
Duplicated	0.347 ± 0.005	0.349 ± 0.005

## Discussion

Here we report the chromosome-level genome assembly of *B. decipiens*, a native Australian grass species important in grassland rehabilitation. Our comparative analysis revealed that this species is a paleo-polyploid, consistent with previous phylogenetic analysis of the group [29]. Our assembly and comparative analyses revealed a relatively recent WGD. Although the diploid progenitors are unknown, our clustering based on unique repeat signatures grouped the chromosomes into subgenomes. Phylogenetic analysis suggested that the diploid ancestors of the paleo-allopolyploid *B. decipiens* speciated approximately 5.8 MYA. Additionally, we showed evidence of biased fractionation with significantly higher gene retention in one of the subgenomes. This subgenome also appeared to have had more active LTRs just prior to the allopolyploidy event. Consistent with expectations, genes that were retained as duplicates following the WGD event were enriched for functions involving transcription and stress response.

### Patterns of gene loss and retention following allopolyploidy

The 2 subgenomes show asymmetric gene loss, with subgenome A retaining more genes than subgenome B (Table 4). This “biased fractionation” commonly occurs after allopolyploidization [53] and has been observed in *Arabidopsis* [21], maize [22, 54], and *Brassica* [19], although it is not always observed [55]. Biased fractionation can be a result of genome dominance, where gene expression tends to be higher in one subgenome compared to the other, leading to greater gene loss in the subgenome with reduced expression [19, 20, 23]. Unlike some previous studies [25, 26, 50], we did not find a difference in the density of TE flanking genes between the subgenomes. Differences in the density of methylated TE nearby genes are thought to contribute to biased fractionation in some paleopolyploids [24, 56, 57]. DNA methylation is known for its role in epigenetic gene silencing [51, 52, 58] and in restricting TE activity [59]. Interestingly, subgenome A also appears to have had more active LTRs at the time of the most recent allopolyploidization event, as evidenced by the greater number of diagnostic *k*-mers and LTR families associated with subgenome A (Supplementary Fig. S3). It would be interesting to explore if the higher LTR activity prior to the WGD (Fig. 4B) and the dominance of subgenome A (Table 4) were related. More retained genes and more active TEs have also been observed in the dominant subgenome of *Miscanthus* [46], but further investigation into the relationship between TE activity and genome dominance is warranted.

Functions overrepresented among the genes retained as duplicates and the genes retained as single copy in the 2 subgenomes are aligned with well-established hypotheses and empirical work [60–64]. Genes containing domains responsible for functions such as RNA modifications and transmembrane activity tend to revert to singletons in plants [63]. Congruently, we identified genes related to RNA modification and transmembrane activity in our GO enrichment analysis of single-copy genes (Table 5A, Supplementary Fig. S4A). We also identified enriched GO terms related to organelles such as chloroplast, mitochondria, and endoplasmic reticulum in the single-copy gene set. Genes encoding functions related to organelles are commonly retained as single copies [61]. The deleterious effect of altering the gene dosage balance could explain the retention of single-copy genes responsible for organelle-mediated processes such as photosynthesis in chloroplasts and respiration in mitochondria. These functions involve proteins from both the organelle and nuclear genome. The interactions between these nonnuclear genomes are regulated to maintain the balance of the protein products created from them [65–67]. During a WGD, this balance could get affected as only the nuclear genome is duplicated, not the organelle's [14]. Alternatively, biased fractionation may preserve coadapted genes [68]. Nuclear encoded genes performing organelle functions and organelle genes have coevolved in each ancestral genome separately, and biased gene loss and reversion to a single copy might maintain coadapted gene complexes and prevent negative interactions of genes between the subgenomes [68]. Alongside genes reverting to single copies, many genes were retained as duplicates. Genes coding for subunits of multimeric proteins or complexes, transcription factors, and signal transduction mechanisms are biased to avoid fractionation [60, 62, 64]. GO terms related to transcription, signal transduction, and protein biosynthesis were overrepresented among retained duplicate genes (Table 5B, Supplementary Fig. S4B). The gene dosage hypothesis explains these patterns of biased retention [16]: if either gene copy that codes for a dosage-sensitive gene product that interacts with other gene products is lost, the dosage imbalance may be deleterious to the organism. We also found that retained duplicated genes had an overrepresentation of GO terms related to response to external stressors like water deprivation, salt stress, and response to abscisic acid signaling (Table 5B, Supplementary Fig. S4B). Genes that give plants the ability to respond to various environmental stresses are frequently retained as duplicates after WGD [69]. As neofunctionalization and subfunctionalization can be possible fates of genes retained as duplicates [70, 71], retention of such genes may promote adaptive evolution to abiotic stresses [72–75]. Multiple paleo-polyploidization events that occurred independently throughout the history of evolution of angiosperms may have promoted the diversification of this clade across a wide range of environmental conditions by contributing to adaptation to new environments and stress [76].

### The evolutionary history of the Andropogoneae

Species in the Andropogoneae clade, which *B. decipiens* belongs to, are dominant in modern-day C4 grasslands. Most allopolyploidization events in the Andropogoneae occurred recently, in the late Miocene [29], which coincides with the expansion of C4 grasslands [77]. Our phylogenetic analysis suggests the speciation event leading to the ancestral genomes occurred at the end of the Miocene, approximately 5.8 MYA, which corresponds to other estimates reported for *Bothriochloa* spp. [29]. It also appears to have occurred at a similar time as the speciation event leading to the subgenomes A and B in *Miscanthus* [46]. For *B. decipiens*, the insertion times of the subgenome-specific LTRs suggest that these species were diverged for up to 4 million years before the hybridization event, although the date estimates are prone to error due to substitution rate variation among LTR families [78].

The genus *Bothriochloa* belongs to a group known as the BCD clade along with the 2 other genera, *Capillipedium* and *Dichanthium* [79]. Species in this clade are able to interbreed even though they are morphologically diverged. *Bothriochloa bladhii* has been identified as a compliospecies, which is a species able to absorb genomes from different species in the BCD complex [29, 79]. However, *B. decipiens* is not included in the early studies on the relationships among the BCD clade [31]. Interestingly, a more recent phylogenetic study of species from the BCD clade in Australia suggested that *B. decipiens* may be an ancestor of this compliospecies complex because it had direct phylogenetic relationships with other species in the BCD clade, including *B. bladhii*, making *B. decipiens* a key species to further our understanding of the evolution of this group [33]. Due to advances in sequencing technology and assembly methods, we now have the opportunity to assemble large and complex genomes to chromosomal levels and further our understanding of the evolution of such diverse and complex groups as the BCD clade. Our high-quality reference genome should spur future comparative genomic studies of allopolyploidy and hybridization in this clade and in grasses more generally.

Most allopolyploidization events in the Andropogoneae are recent, such as the one we report in *B. decipiens*. These events are concurrent with the expansion of C4 grasslands, and some studies argue that the allopolyploidy gave these grasses the ability to adapt to new environments, thereby enabling successful establishment and expansion [80, 81]. The biased retention of duplicated genes related to stress response provides support for this hypothesis, but a causal relationship between allopolyploidization and C4 grassland expansion remains to be established. Future analysis examining the adaptive significance of retained duplicates using both comparative and population genomic approaches across a greater number of taxa, including groups with frequent WGD such as the Andropogoneae, will further our understanding of the adaptive significance of allopolyploidy and its potential role in niche expansion for C4 grasses.

### Potential implications

This genome will be an important resource for population genomic studies involving native grasses in this genus. Such analyses will shed light on the adaptive genetic landscape of these important foundation species, which could play a critical role in the development of climate change–resilient grassland restoration practices in Australia and elsewhere [82, 83]. Further, this genome will be important in broader comparative analyses of the Andropogoneae, which should provide greater insight into the evolutionary significance of allopolyploidy.

## Methods

### Species description

*B. decipiens* (blue pitted grass) is a warm-season, perennial, tufted grass that can grow up to a meter in height [84]. Due to its ability to establish well from direct seeding on many soil types and the ability to withstand pressure caused by overgrazing, it has become an important species for rehabilitation. It is widespread in subtropical New South Wales (NSW) and Queensland as well as tropical Queensland [85]. It is a close relative and phenotypically similar to the polyploid *Bothriochloa macra*, which is a widespread native grass species in southeastern Australia. The sporophytic chromosome number of *B. decipiens* is reported to be 2n = 40 [86], and it is a genetic diploid.

### Sample collection

The seeds used to grow the diploid *B. decipiens* accession COB1-7 used in this study were collected from Cobbitty, NSW (34°03′N, 150°68′E). Using these seeds, a plant was grown and maintained at Monash University, Clayton to obtain leaf and inflorescence tissue samples for DNA and RNA extractions for the study.

### Flow cytometry

We used FCM to estimate the genome size and predict the relative ploidy of 24 populations of *B. macra* and *B. decipiens* collected from different locations in states Victoria and NSW and also sought evidence for within-population variation in ploidy. Estimating ploidy from different populations was necessary as *B. decipiens* and polyploid *B. macra* are extremely similar morphologically, and ploidy is the best method to reliably distinguish the 2 species [33]. We estimated the ploidy of at least 5 plants from each population following a modified plant FCM protocol [87]. Leaf samples from each population were collected from greenhouse-grown plants and immediately placed on ice for same-day cytometric analysis. Two DNA genome size standards were selected, *Solanum lycopersicum* (2C = 1.96) and *Pisum sativum* (2C = 9.09), and grown from seed. Approximately 40 mg of fresh leaf material was used for each sample and placed into a 2.0-ml tube with a single 3-mm tungsten carbide bead and 436 µL of an ice-cold nuclei suspension buffer modified from de Laats buffer (1984): 15 mM HEPES, 1 mM EDTA, 0.2% (v/v) Triton X-100, 80 mM KCl, 20 mM NaCl, 300 mM sucrose, 0.5 mM spermine, 15 mM β-mercaptoethanol, and 0.25 mM PVP, adjusted to pH 7. Samples were placed in a Qiagen Tissuelyser II and ground for 24 seconds at 25 hertz, and then the sample rack was reversed and ground again. The homogenate was filtered through 2 layers of Millipore Miracloth (22–25 µm pore size) suspended in a 3-piece nozzle. Then, 1 µL of 10 µg/µL RNAse was added for every 100 µL of filtrate and incubated at 37°C for 20 minutes. Next, 15 µL of 0.1 µg/444 µL propidium iodide station solution was added to the filtrate, and samples were run on the BD Accuri C6 Cytometer using the settings outlined in [88]. Internal standards were run separately on the cytometer at the beginning and end of the session—no change in dye fluorescence was recorded over that period of time.

A total of 38 samples produced an observable signal in the FCM run. All samples, excluding standards, were run in a blind fashion so that prior knowledge of expected ploidy did not bias the identification of nuclei peaks. The 2C values were determined for all *Bothriochloa* samples by comparing the FL2-A value of the sample to the internal standards, *Solanum* and *Pisum*, which have a known 2C value of 1.96 and 9.09 pg, respectively [87]. The average 2C genome size of diploid and polyploid plants was 2.80 pg (range, 2.56–2.99 pg) and 5.38 pg (range, 4.94–5.91 pg), respectively (Supplementary Fig. S5). Only 1 population (COB1) consisted of diploid individuals, and all individuals from this population were tested to confirm our findings. The COB1–7 accession was 2.56 pg, which leads to a haploid genome size estimate of 1.25 Gb (Supplementary Fig. S6). The polyploid samples were the closely related and phenotypically similar *B. macra*.

### DNA extraction

For DNA extraction, fresh leaf tissue was collected from diploid individual COB1–7, flash frozen in liquid nitrogen, and stored at −80°C. The tissue was then shipped to Dovetail Genomics for the completion of DNA extractions by using the following steps. To obtain high molecular weight DNA for 10X Genomics linked read sequencing, 1.8 g of leaf material was ground with mortar and pestle to a fine powder, to which 200 ml prewarmed CTAB and 100 µL BME was added. This was incubated at 68°C for 15 minutes. Once incubated, a mixture of 2× phenol chloroform, 1× isoamyl, and 0.7× isopropanol was added and centrifuged to form a pellet. The pellet was combined with 9.5 ml G2 DNA enhancer buffer solution, 200 µL protease, and 19 µL RNase. Again, the mixture was incubated at 50°C for 1 hour. The precipitated genomic DNA was used in library construction.

### 10X library preparation sequencing and 10X assembly

Genomic DNA (gDNA) with an adjusted concentration between 1.0 and 1.25 ng/µL was used to prepare the whole-genome sequencing libraries using the Chromium Genome Library and Gel Bead Kit v.2, Chromium Genome Chip Kit v.2, Chromium i7 Multiplex Kit, and Chromium controller according to the manufacturer's instructions (10X Genomics). Genomic DNA was combined with Master Mix, a library of Genome Gel Beads, and partitioning oil to create Gel Bead-in-Emulsions (GEMs) on a Chromium Genome Chip. The GEMs were isothermally amplified with primers containing an Illumina Read 1 sequencing primer, a unique 16-bp 10X barcode, and a 6-bp random primer sequence. Barcoded DNA fragments were recovered for Illumina library construction. The amount and fragment size of post-GEM DNA were quantified prior using a Bioanalyzer 2100 with an Agilent high-sensitivity DNA kit. Prior to Illumina library construction, the GEM amplification product was sheared on an E220 Focused Ultrasonicator (Covaris) to approximately 350 bp. Then, the sheared GEMs were converted to a sequencing library following the 10X standard operating procedure. The library was quantified by quantitative polymerase chain reaction (qPCR) with a Kapa Library Quant kit (Kapa Biosystems–Roche) and sequenced on a NovaSeq6000 sequencer (RRID:SCR_020150) (Illumina) with paired-end 150-bp reads.

### Chicago library preparation and sequencing

A Chicago (RRID:SCR_014941) library was prepared as described in [34]. Briefly, ∼500 ng high molecular weight gDNA was reconstituted into chromatin in vitro and fixed with formaldehyde. Fixed chromatin was digested with DpnII, the 5′ overhangs were filled in with biotinylated nucleotides, and then free blunt ends were ligated. After ligation, crosslinks were reversed and the DNA purified from protein. Purified DNA was treated to remove biotin that was not internal to ligated fragments. The DNA was then sheared to ∼350-bp fragments and sequencing libraries were generated using the NEBNext Ultra II kit with Illumina-compatible indices. Biotin-containing fragments were isolated using streptavidin beads before PCR enrichment of each library. The libraries were sequenced on an Illumina HiSeq X Ten (RRID:SCR_016385) to produce 467 million 2 × 150-bp paired-end reads.

### Dovetail Hi-C library preparation and sequencing

A Dovetail Hi-C library was prepared as described in [89]. Briefly, for each library, formaldehyde was used to fix chromatin in the nucleus in place. Fixed chromatin was digested with DpnII, the 5′ overhangs were filled in with biotinylated nucleotides, and then free blunt ends were ligated. After ligation, crosslinks were reversed and the DNA purified. Purified DNA was treated to remove biotin that was not internal to ligated fragments. The DNA was then sheared to ∼350-bp mean fragment size, and sequencing libraries were generated using NEBNext Ultra enzymes and Illumina-compatible adapters. Biotin-containing fragments were isolated using streptavidin beads before PCR enrichment of each library. The libraries were sequenced on an Illumina HiSeq X Ten to produce 400 million 2 × 150-bp paired-end reads.

### Genome assembly

The 10X sequence data were assembled de novo with Supernova (RRID:SCR_016756) [90]. This de novo assembly, along with Chicago library reads and Dovetail Hi-C library reads, was used as input data for HiRise (RRID:SCR_023037), a proprietary software designed specifically for using proximity ligation data to scaffold genome assemblies [34]. An iterative analysis was conducted. First, Chicago library sequences were aligned to the draft *de novo* assembly from Supernova using SNAP (RRID:SCR_007936) [91]. The separations of Chicago read pairs mapped to the draft scaffolds were analyzed by HiRise to estimate the genomic distance between read pairs, and the model was used to identify and break putative misjoins, score prospective joins, and make joins above the default threshold. After aligning and scaffolding Chicago data, Dovetail Hi-C library sequences were aligned and scaffolded following the same method. After scaffolding using Chicago and Hi-C library data, visual inspection of the contact maps identified 2 misjoin events in the largest scaffold and 1 other scaffold (black circles in Supplementary Fig. S7). Manual corrections were performed using link density plots in Juicebox (RRID:SCR_021172) [92] to make breaks within those 2 scaffolds and produce the link density plot for the final assembly (Supplementary Fig. S8).

### Messenger RNA sequencing library preparation

RNA was extracted separately from young (a few weeks) and old (a year) tissue (leaf and stem) from 1 individual using the Qiagen RNeasy kit. RNA was pooled and a library was synthesized and sequenced by Genewiz on an Illumina Novaseq 6000 platform in 2 × 150-bp mode, resulting in 64,756,621 reads.

### Transcriptome assembly

Raw RNA-seq reads were first cleaned by trimming the adapters using Trimmomatic v. 0.38 (RRID:SCR_011848) with the parameter “ILLUMINACLIP:TruSeq3- PE.fa:2:30:10:2:keepBothReads LEADING:3 TRAILING:3 MINLEN:36” [93]. The trimmed reads were used to assemble the transcriptome using Trinity v.2.8.5 (RRID:SCR_013048) [37] using default parameters. Assembly statistics for the transcriptome assembly was obtained after running the TrinityStats.pl script on the reference transcriptome assembly fasta file (obtained from Trinity).

### Annotation of repetitive sequences

A custom repeat library was constructed following recommendations of the MAKERP pipeline for advanced repeat construction [94]. Both structure-based and homology-based approaches were used to increase the power to detect repeats. Sequences of miniature inverted repeat transposable elements (MITEs) were collected using MITE-Hunter (RRID:SCR_020946) [95] using all the default parameters. LTR retrotransposons were collected using LTRharvest (RRID:SCR_018970) and LTR-digest [96, 97]. The candidates were filtered for false positives caused by other tandem repeats such as centromeres, tandem gene duplications, or other transposable elements. A common feature of these sequences is that the alignment between the 2 putative “LTRs” often extends beyond the boundary of the “LTR” into the flanking region, so these false-positive candidates were excluded by identifying those sequences whose alignments extended beyond the LTR boundary into the flanking region. Representative sequences (exemplars) were chosen as described previously [54] to reduce the redundancy of the LTR. Then, other repetitive elements were collected by first masking the genome sequence with the previously obtained MITE and LTR sequences. Next, unmasked sequences were extracted and processed by RepeatModeler v.2.0.3 (RRID:SCR_015027) [98] by providing a database built out of the unmasked sequences as an input, to identify additional repeats. As many repeats carry gene fragments, all the collected repetitive elements were searched against a plant protein database that contains those from swissprot plant protein and NCBI Refseq plants [99] with transposon proteins excluded. Elements with significant hits to genes were removed along with 50 bp upstream and downstream of the hit. If the remaining sequence was less than 50 bp, then it was completely excluded. Sequences matching the plant proteins as well as 50 bp of flanking sequences were removed using the package ProtExcluder [94]. After this, if the remaining portion of the sequence was shorter than 50 bp, the entire sequence was excluded. Sequences of all the identified repetitive elements were joined together to form a final custom repeat library to be used to mask the repetitive elements of the genome in the MAKER genome annotation protocol [38]. To identify the type of repeat (including the repeat family), the unidentified repeats from RepeatModeler, as well as the LTRs and MITES from the custom library, were searched against 2 transposase databases. The first was Tpases020812 [100]. This database is composed of transposase protein sequences from the RepeatMasker v.4.1.1 (RRID:SCR_012954) [101] and from 2 other sources [102, 103], and it was searched using BLASTX (RRID:SCR_001653) [104]. The second was the publicly available Dfam-curated library of repeats [105], which was searched using Hmmer v.3.3.1 (RRID:SCR_005305) [106] implemented through [107]. The sequences that matched the database were classified according to their top hits. Using this custom repeat library, RepeatMasker version 4.1.1 [101] was used to mask the genome and identify the distribution of repeat types.

### Genome annotation

MAKER v.3.01.03 (RRID:SCR_005309) [38] genome annotation pipeline was used to annotate the genome. The input files provided for the first run were the genome assembly fasta file (Genbank accession JALGXP000000000), the reference transcriptome assembly fasta file (obtained from Trinity), and the protein homology evidence from a plant protein database [99] that combines the Swissprot plant protein database and NCBI Refseq for plants excluding transposable elements. Repetitive regions were masked using our custom repeat library. Additional regions with low complexity were soft masked using RepeatMasker v.4.1.1 [101]. Iterative runs of MAKER v.3.01.03 [38] were undertaken in order to train the gene predictors SNAP v.2013-11-29 [40] and AUGUSTUS v.3.3.3 [39] as recommended by [38]. The first round of annotation was based on alignments of the transcriptome to the genome. For the first round, the est2genome option in the MAKER control file was set to 1 to allow MAKER to infer gene models directly from the RNA-seq evidence in the transcriptome. After the completion of the first round of annotations, gene models with an AED (Annotation Edit Distance) score of 0.25 or greater and a length of 50 or more amino acids were retained and used to train SNAP v.2013-11-29 [40] to obtain a SNAP HMM file. We then trained AUGUSTUS v.3.3.3 [39] using BUSCO v.3.0.2 (RRID:SCR_015008) [108]. First, training sequences were identified using the gene models predicted by MAKER from the first run by excising regions with messenger RNA annotations and 1,000 bp on either side. These were used to run BUSCO using the embryophyte set of conserved genes and an initial HMM model from rice. After training both SNAP and AUGUSTUS, MAKER was run again, with SNAP HMM and Augustus files. A total of 3 rounds of training for each gene predictor were run. We used the script genestats [109] to calculate the numbers and lengths of genes, exons, introns, and untranslated region (UTR) sequences present in the predicted gene models by the final MAKER run (Supplementary Table S1). We ran BUSCO v.5.1.3 [110] with the eukaryota_odb10 lineage dataset on the predicted transcript fasta file by MAKER to assess the quality and the completeness of the annotated genome.

### Detailed identification of repeat family diversity in the genome

Apart from constructing a repeat library to be used in the MAKER genome annotation pipeline above, EDTA v.2.0.0 [41] was used to obtain more information about the repeat diversity in the genome. Input files used to run the EDTA pipeline for a more accurate repeat annotation were the genome assembly fasta file, fasta file with the coding sequences of *B. decipiens* obtained after the final annotation round of MAKER above, and a BED file with the locations of genes in the genome as predicted by the MAKER annotation pipeline above.

### Subgenome and homoeologous exchange identification

First, we identified the 20 largest scaffolds (> 40 Mb; representing 1.11 Gb of the 1.22-Gb genome). These scaffolds were then aligned against themselves using Minimap2 v.2.1.8 (RRID:SCR_018550) [44] to identify scaffolds that shared homology and synteny that would indicate putative homoeologous chromosomes. The alignments were plotted using the R/pafr package v.0.0.2 [111]. Of these 20 scaffolds, 10 pairs (with more than 50% matching across both scaffolds) were identified as the pairs of homoeologous scaffolds (Supplementary Fig. S1). As there are no genomic data from any close diploid relatives that do not share the most recent allopolyploidization event, we clustered the putative homoeologous chromosomes based on repeat abundance using *k*-mer distributions. We partitioned the *B. decipiens* genome into subgenomes A and B by modifying the methods described in [46]. Specifically, we first identified 13 base pair sequences (13-mers) using Jellyfish v. 2.3.0 (RRID:SCR_005491) [112] and retained *k*-mers at high abundance in the assembly (100× or above). For each pair of scaffolds, we compared the counts of these 13-mers, identifying those that differed in abundance by 3-fold or more between scaffolds. To control for any differences in scaffold length impacting this assessment, we further reduced the set of diagnostic 13-mers to those that retained a 3-fold difference after standardizing *k*-mer count for the scaffold length, while keeping only those diverging in the same direction as the absolute *k*-mer count. Hierarchical clustering of scaffolds based on difference in 13-mer counts was used to identify putative subgenomes as implemented in the R/ComplexHeatmaps package (RRID:SCR_017270) [113].

We tested for homoeologous exchange among the subgenomes using an HMM implemented in the R/HMM package [47]. We used the most common *k*-mer type (A or B) in 1-Mbp windows as the observed states and the subgenome type for each of the 1,121 windows. The initial HMM used equal starting probabilities and transition probabilities of 0.01. We trained the HMM emission probabilities (viterbiTraining) using scaffold 5 and scaffold 15 as they appeared not to be subject to any subgenome exchange based on the A and B *k*-mer density plots (Supplementary Fig. S3).

We examined subgenome-enriched LTRs, as differences in LTR activity in parental species can help differentiate subgenomes and can be used to assess the timing of allopolyploidy. LTRs were used for this because they are rapidly evolving, making it easy to differentiate between related subfamilies. The timing of insertions can be calculated by examining the substitution rates for members of the same subfamily using the 5′ and 3′ regions [46]. Specifically, intact retrotransposons in the genome were identified using LTR-HARVEST [96]. The “best” option was used for pairing overlapping LTR sequences, allowing the inner sequences of the retrotransposons to contain gaps. We performed an all-versus-all BLAST (RRID:SCR_004870) [42] on the LTR segments of the identified LTRs with an e-value cut off of 1e-2. Hits with the percentage of alignment between query and subject equal to or greater than 90% over their entire length were selected. We then used the MCL algorithm [114] to cluster the filtered blast alignments into retrotransposon subfamilies using an inflation parameter (-I) of 3. We counted the occurrence and the total base pairs that each LTR subfamily obtained from above clustering in the putative A and B subgenomes identified above. We identified LTR subfamilies that were 3 times more common in one of the subgenomes using both occurrence and bp count. Then we determined if these repeats overlapped with multiple A or B genome-preferred *k*-mers to confirm that *k*-mers were representing longer repetitive sequences and to confirm that these *k*-mers were marking repeat expansion that occurred just before the allopolyploidy event.

### Gene function prediction

The predicted protein sequences obtained from the final run of MAKER were aligned to the UniProtKB/Swiss-Prot [115] and TAIR10 [116] protein databases using BLASTP (RRID:SCR_001010) [42] with an e-value cutoff of 1.0e-5. The GO term associated with the best hit for each BLASTP search was identified in each of the 3 databases above and assigned to the *B. decipiens* query. InterProScan v. 5.51–85.0 (RRID:SCR_005829) [117] was used to search the query protein fasta against the Pfam [118] protein family database and identify functional protein domains. Pfam accessions and GO terms were retrieved for the *B. decipiens* query sequences. The query protein sequences were BLAST searched against the KEGG database [119–121] using the online tool KofamKOALA—KEGG orthology search [122] with an e-value cutoff of 1.0e-5. KEGG orthology terms and enzyme codes were retrieved for each hit.

### Genome synteny and whole-genome duplication

To determine if the *B. decipiens* genome had undergone WGD, a reciprocal BLASTP was conducted using *B. decipiens* protein sequences as the query against themselves with a minimum e-value greater than 1.0e-5. Then, MCScanX [43] was used to identify syntenic blocks within the genome. The collinear blocks obtained via MCScanX between the putative A and B subgenomes of *B. decipiens* were visualized using Synvisio [123]. Similarly, we identified collinear blocks between the 2 putative subgenomes of *B. decipiens* and *S. bicolor* by conducting a reciprocal BLASTP comparing protein sequences from each species using MCScanX and plotted the results using Synvisio.

### Estimating the timeline of paleotetraploidy

We estimated the timing of speciation events in the Andropogoneae using *P. hallii* and *S. italica* as outgroups. The reference gene sets for *S. bicolor* v3.1.1, *P. hallii* v2.2, *S. italica* v2.1, *M. sinensis* v7.1, and *Zea mays* (B73 RefGen_v4) were downloaded from Phytozome v12.1 (RRID:SCR_006507) [124]. The *Saccharum spontaneum* reference gene set [125] was also downloaded. We separated the A and B subgenomes of *M. sinensis* (as identified in [46]), as well as those of *B. decipiens*, to compare the timeline of the paleopolyploidy events between *Miscanthus* and *B. decipiens* species. We identified 1:1 orthologs between all species (or subgenomes) using OrthoFinder v.2.3.8 (RRID:SCR_017118) [48]. A core set of 392 single-copy genes was retained, and multiple sequence alignments were performed for each orthologous cluster using Genodup [126] and Mafft (RRID:SCR_011811) [127]. Poorly aligned regions were removed using Gblocks 0.91b (RRID:SCR_015945) [128], and the final alignments were concatenated into a single alignment.

### Phylogenomic analysis

The best model of evolution was inferred using jModelTest2 [129, 130]. A phylogenetic tree was constructed using RAxML (RRID:SCR_006086) [131] with the GTRGAMMAI model of evolution and 1,000 bootstrap replicates. *S. italica* and *P. hallii* were designated as outgroups.

### Divergence date estimation

Divergence among lineages in the phylogeny were estimated from the concatenated alignment using BEAST v.2.5 (RRID:SCR_010228) [132, 133] after using bModelTest [134] to infer the best substitution model. Parameters included the general time-reversible (GTR) substitution model with unequal frequencies, 4 gamma categories, estimated shape, and invariant sites. We chose a relaxed lognormal clock with estimated clock rates. We set priors to constrain the estimated dates at the *Setaria–Panicum* (12.8–20 MYA) and the Andropogoneae (13–21.2 MYA) nodes, using a uniform distribution between the minimum age and maximum ages of divergence times obtained from the TimeTree database [135]. BEAST2 (RRID:SCR_017307) [132] analysis was conducted for 50 million generations and logging at every 5,000 trees. Convergence between runs was assessed with Tracer v.1.6 [136]. We used Tracer v.1.6 to visualize replicate analyses and determined convergence when the joint density (posterior) of each replicate stabilized to an overlapping, stationary distribution. We continued analyses until the ESS (Effective Sample Size) reached at least 200. Trees were summarized with TreeAnnotator [133] using a burn-in value of 20%.

### Timing of subgenome-specific LTR expansions

We aligned the LTRs of each LTR family cluster using Mafft (RRID:SCR_011811) [127]. We computed Jukes–Cantor distance matrices using the R/ape package [137]. We estimated the divergence times of each LTR family as k/2r (k = divergence, r = substitution rate) [138]. We used 1.3 × 10^−8^ as the substitution rate per site per year [139].

### Determination of biases in subgenome gene retention

We analyzed the collinear blocks between *S. bicolor* and *B. decipiens* to assess differences in retention of duplicated genes between subgenomes. Subgenome-specific retention was inferred as the number of genes retained in a given subgenome divided by the number of inferred ancestral (i.e., preduplication) gene numbers. Consequently, we calculated the number of ancestral (preduplication) genes as those orthologous genes present in *S. bicolor* and in 1 or both of the 2 subgenomes. We then compared this number to the total number of genes present only in subgenome A, only in subgenome B, or in both subgenomes. We then used a 2-sided Fisher's exact test to determine if there was a significant difference in retention of genes between the subgenomes under the null hypothesis that gene loss between subgenomes was random. We also used the results from OrthoFinder (RRID:SCR_017118) to confirm this pattern. Specifically, we identified 1:1 and 1:2 orthologs between *S. bicolor* and *B. decipiens*, only retaining 1:2 orthologs that were mapped to chromosomes on both *B. decipiens* subgenomes.

Differences in the function of duplicated genes retained after the WGD compared to those returning to single copy were tested through gene enrichment analysis using R/topGo (RRID:SCR_014798) [49] where a Fisher's exact test was used to compare the expected number of genes to the observed number of genes. All single-copy genes in A or B, or duplicated genes in both A and B, were used as foreground genes and the remaining ancestral genes (retained duplicated or single copy) as background genes.

### TE densities between the subgenomes

For this, we used the software pipeline TE density [140]. Here, TE density is defined as the number of TE-occupied base pairs in a given base pair range (window) divided by the window size. First we calculated the TE density relative to all the genes present in the genome by providing the gene annotation BED file obtained through the genome annotation by MAKER and the TE annotation BED file obtained via the EDTA v.2.0.0 [41] pipeline. The window size was defined as 5,000 bp for the calculations, although larger (50,000 bp) and smaller window sizes (500 bp) yielded qualitatively similar results. The program outputs calculated TE density value for both upstream and downstream of each gene. We then used a Mann–Whitney *U* test to determine if there was a significant difference between the average TE density in both upstream and downstream of genes found in the 2 subgenomes. We then investigated if there were any differences in TE density near genes identified as single copy or duplicated in the above analysis of biases in gene retention (McScanX scanning method). To do this, we used a Mann–Whitney *U* test to determine if there was a significant difference between the average TE density in both upstream and downstream of genes found retained as single copy or as duplicated in the genome.

## Availability of Source Code

R scripts used in this study can be found in the public GitHub repository [141].

## Abbreviations

BCD: *Bothriochloa, Capillipedium*, and *Dichanthium*; BLAST: Basic Local Alignment Search Tool; BLASTP: Basic Local Alignment Search Tool Program; bp: base pairs; BUSCO: Benchmarking Universal Single-Copy Orthologs; FCM: flow cytometry; Gb: Giga bases; gDNA: genomic DNA; GO: Gene Ontology; HMM: hidden Markov model; KEGG: Kyoto Encyclopedia of Genes and Genomes; LTR: long terminal repeat; Mb: mega base pairs; MITE: miniature inverted repeat transposable element; MYA: million years ago; NCBI: National Center for Biotechnology Information; NSW: New South Wales; PCR: polymerase chain reaction; RNA-seq: RNA sequencing; TE: transposable element; UTR: untranslated region; WGD: whole-genome duplication.

## Funding

This study was supported by the Hermon Slade Foundation (grant number HSF1703), Monash Graduate Scholarship, Monash University, Monash International Tuition Scholarship, Monash University and Denis and Maisie Carr Award and Travel grant 2020, and School of Biological Sciences, Monash University.

## Supplementary Material

giad034_GIGA-D-22-00164_Original_Submission

giad034_GIGA-D-22-00164_Revision_1

giad034_Response_to_Reviewer_Comments_Original_Submission

giad034_Reviewer_1_Report_Original_Submission Dhanushya Ramachandran -- 8/27/2022 Reviewed

giad034_Reviewer_2_Report_Original_Submission Michael McKain -- 9/19/2022 Reviewed

giad034_Reviewer_2_Report_Revision_1 Michael McKain -- 3/15/2023 Reviewed

giad034_Reviewer_3_Report_Original_Submission Kehua Wang -- 10/5/2022 Reviewed

giad034_Supplemental_Files

## Data Availability

Raw reads for the genome assembly have been deposited under BioProject accession number PRJNA819081. Illumina library raw reads (namely, 10X, Chicago, and Hi-C data) have been deposited in the Sequence Read Archive (SRA) under study accession numbers SRR18458736, SRR18471564, and SRR18471563. RNA-seq data have been deposited under SRA accession number SRR18471562. Genome assembly of *B. decipeins* is deposited in the NCBI genome database under the accession JALGXP000000000. All supporting data are available via the *GigaScience* repository, GigaDB [142].
