# Supplementary material for: Genome assembly of an Australian native grass species reveals a recent whole-genome duplication and biased gene retention of genes involved in stress response
Source: Gigascience. 2023 May 12;12:giad034. doi: 10.1093/gigascience/giad034 (PMC10176504; doi:10.1093/gigascience/giad034)
Supplement: giad034_GIGA-D-22-00164_Original_Submission [file giad034_giga-d-22-00164_original_submission.pdf]

GigaScience

Genome assembly of an Australian native grass species reveals a recent whole genome duplication and biased gene retention of genes involved in stress response

--Manuscript Draft--

**Manuscript Number:**	GIGA-D-22-00164
**Full Title:**	Genome assembly of an Australian native grass species reveals a recent whole genome duplication and biased gene retention of genes involved in stress response
**Article Type:**	Research
**Funding Information:**	Hermon Slade Foundation (HSF1703) — Dr Kathryn Hodgins
**Abstract:**	Background<br><br>The adaptive significance of polyploidy has been extensively debated and chromosome level genome assemblies of polyploids can provide insight into this topic. The Australian grass, Bothriochloa decipiens, belongs to the BCD clade, a group with a complex history of hybridization and polyploidy. This is the first genome assembly and annotation of a species that belongs to this fascinating yet complex group.<br><br>Findings<br><br>Using a combination of Illumina short reads, 10X Genomics linked reads and Hi-C sequencing data we assembled a highly contiguous genome of Bothriochloa decipiens , with a total length of 1,218.22 Mb and scaffold N50 of 42.637 Mb. Comparative analysis revealed that the species is a diploidized allotetraploid. We clustered the 20 major scaffolds, representing the 20 chromosomes, into the two sub genomes of the parental species using unique repeat signatures. Found evidence of biased fractionation and differences in the activity of transposable elements between the sub genomes prior to hybridization. Duplicates were enriched for genes involved in transcription and response to external stimuli like drought, supporting a biased retention of duplicated genes following whole genome duplication.<br><br>Conclusions<br><br>Our results support hypotheses explaining the biased retention of duplicated genes following polyploidy and point to differences in repeat activity associated with sub genome dominance. Bothriochloa decipiens is a widespread species with the ability to establish across many soil types, making it useful for ecological restoration of Australian grasslands. This reference genome is a valuable resource for future population genomic research involving Australian grasses which may be helpful in ecological restoration projects.
**Corresponding Author:**	Nissanka Prasangi De Silva, Ph.D<br>Monash University School of Biological Sciences<br>Clayton, Victoria AUSTRALIA
**Corresponding Author Secondary Information:**	
**Corresponding Author's Institution:**	Monash University School of Biological Sciences
**Corresponding Author's Secondary Institution:**	
**First Author:**	Nissanka Prasangi De Silva, B.Sc
**First Author Secondary Information:**	
**Order of Authors:**	Nissanka Prasangi De Silva, B.Sc
	Christopher Lee, Ph.D
	Paul Battlay, Ph.D

Powered by Editorial Manager® and ProduXion Manager® from Aries Systems Corporation

	Alexandre Fournier-Level, Ph.D
	Joslin Moore, Ph. D
	Kathryn Hodgins, Ph.D
**Order of Authors Secondary Information:**	
**Additional Information:**	
**Question**	**Response**
Are you submitting this manuscript to a special series or article collection?	No
**Experimental design and statistics**<br><br>Full details of the experimental design and statistical methods used should be given in the Methods section, as detailed in our Minimum Standards Reporting Checklist. Information essential to interpreting the data presented should be made available in the figure legends.<br><br>Have you included all the information requested in your manuscript?	Yes
**Resources**<br><br>A description of all resources used, including antibodies, cell lines, animals and software tools, with enough information to allow them to be uniquely identified, should be included in the Methods section. Authors are strongly encouraged to cite Research Resource Identifiers (RRIDs) for antibodies, model organisms and tools, where possible.<br><br>Have you included the information requested as detailed in our Minimum Standards Reporting Checklist?	Yes
**Availability of data and materials**<br><br>All datasets and code on which the conclusions of the paper rely must be either included in your submission or deposited in publicly available repositories (where available and ethically	Yes

Powered by Editorial Manager® and ProduXion Manager® from Aries Systems Corporation

appropriate), referencing such data using a unique identifier in the references and in the “Availability of Data and Materials” section of your manuscript.<br><br>Have you have met the above requirement as detailed in our Minimum Standards Reporting Checklist?	

Powered by Editorial Manager® and ProduXion Manager® from Aries Systems Corporation

Manuscript
Click here to access/download;Manuscript;Bdecipiens_genome_gigascienc

Genome assembly of an Australian native grass species reveals a recent whole genome duplication and biased gene retention of genes involved in stress response

Nissanka P. De Silva[1*], Christopher Lee[2], Paul Battlay[3], A. Fournier-Level[4], Joslin L. Moore[5], Kathryn A. Hodgins[6]

[1]School of Biological Sciences, Monash University, Clayton, Victoria, Australia, 3800. Email: nissanka.desilva@monash.edu

[2]School of Biological Sciences, Monash University, Clayton, Victoria, Australia, 3800. Email: Chris.Lee@monash.edu

[3]School of Biological Sciences, Monash University, Clayton, Victoria, Australia, 3800. Email: Paul.Battlay@monash.edu

[4]School of BioSciences, University of Melbourne, Melbourne, Victoria, Australia, 3010. Email: alexandre.fournier@unimelb.edu.au

[5]Arthur Rylah Institute for Environment Research, Heidelberg, Victoria, Australia, 3084. Email: joslin.moore@delwp.vic.gov.au

[6] School of Biological Sciences, Monash University, Clayton, Victoria, Australia, 3800. Email: Kathryn.hodgins@monash.edu

[*]Corresponding author

Abstract

Background

The adaptive significance of polyploidy has been extensively debated and chromosome level genome assemblies of polyploids can provide insight into this topic. The Australian grass, *Bothriochloa decipiens,* belongs to the BCD clade, a group with a complex history of hybridization and polyploidy. This is the first genome assembly and annotation of a species that belongs to this fascinating yet complex group.

Findings

Using a combination of Illumina short reads, 10X Genomics linked reads and Hi-C sequencing data we assembled a highly contiguous genome of *Bothriochloa decipiens,* with a total length of 1,218.22 Mb and scaffold N50 of 42.637 Mb. Comparative analysis revealed that the species is a diploidized allotetraploid. We clustered the 20 major scaffolds, representing the 20 chromosomes, into the two sub genomes of the parental species using unique repeat signatures. Found evidence of biased fractionation and differences in the activity of transposable elements between the sub genomes prior to hybridization. Duplicates were enriched for genes involved in transcription and response to external stimuli like drought, supporting a biased retention of duplicated genes following whole genome duplication.

Conclusions

Our results support hypotheses explaining the biased retention of duplicated genes following polyploidy and point to differences in repeat activity associated with sub genome dominance. *Bothriochloa decipiens* is a widespread species with the ability to establish across many soil types, making it useful for ecological restoration of Australian grasslands. This reference genome is a valuable resource for future population genomic research involving Australian grasses which may be helpful in ecological restoration projects.

Key words - genome assembly, annotation, paleo- allopolyploidization, whole genome duplication (WGD), biased fractionation

Background

Whole genome duplication (WGD), or polyploidy, occurs via the doubling of chromosomal material either involving one species (autopolyploidy) or by hybridization of two species (allopolyploidy). The polyploid origins of many plant species has long been recognized [1,2], but while polyploidy is commonly observed in angiosperms, its evolutionary importance has been controversial. Some studies support the hypothesis that polyploidy can drive rapid adaptive evolution [3,4], while others have argued that polyploidy has played a minimal role in evolution and contributed little to adaptation [5]. However, there is growing evidence that ancestral WGD events caused key changes in major angiosperm clades that led to their successful diversification [6].

After a WGD event various molecular changes occur to restore the diploid state, known as diploidization [7–11] via genome rearrangement, gene loss and epigenetic change [7,12]. These molecular processes are collectively known as fractionation. Genes that encode DNA repair mechanisms and organelles tend to revert to single copy status following WGD events [13,14]. However, other duplicated genes may be retained for a long time and escape deletion or pseudogenization [15]. Several theories explain the patterns of duplicated gene retention and their evolutionary fate. For instance, the gene balance hypothesis states that genes coding for products that are dose-sensitive are protected from fractionation because if fractionated, the stoichiometry of the products and other gene products that they interact with will be

affected and may bring about negative or lethal effects to the organism [16]. These principles are thought to apply to genes responsible for controlling functions related to gene regulation such as transcription factors or kinases acting as hubs with the potential to control entire gene networks [17,18].

WGD can also give rise to functionally distinct sub genomes. Biased fractionation through the preferential loss of the duplicated genes from the same sub-genome has been observed in many polyploids [19–22]. Gene expression also tends to be biased between retained homeologs, with greater mRNA abundance observed in regions of the genome where gene loss is less common than in corresponding regions (homeologs) with more gene loss [19,20,23,24]. The less fractionated and more highly expressed sub genome is referred to as the dominant sub genome, and this asymmetry frequently occurs in hybrids with divergent parental genomes [25].

Arguably the most successful plant family in terms of occurrence, ecological dominance and species richness is Poaceae (grasses) [26] and approximately 80% of this family are polyploids [27]. Our study species, *Bothriochloa decipiens*, belongs to the tribe Andropogoneae (subfamily Panicoideae). This tribe contains species that have ecological as well as economic importance, and independent allopolyploid events have occurred in exceptionally high numbers in this group [28]. *Bothriochloa decipiens* is part of a cosmopolitan grass genus [29] closely related to *Capillipedium* and *Dichanthium* (together referred to as BCD). These three genera have the ability to interbreed despite their morphological differences, and the term compilospecies was coined to describe this hybrid complex [30,31]. *Bothriochloa decipiens* is a diploid member of the group and may be a parental diploid species to some of the present-day allopolyploids in the clade [32].

Here we report a chromosome-level genome assembly, annotation and comparative analysis of a species in the BCD clade, *Bothriochloa decipiens*. This is the first genome assembly and annotation of a species that belongs to this fascinating yet complex group. Our highly contiguous *B. decipiens* genome assembly showed clear evidence of recent paleo-polyploidy. Using repeat signatures diverged between putative homeologous chromosomes we were able to organise chromosomes into sub genomes, allowing estimation of the timing of the speciation event prior to the most recent allopolyploidy event in this species. We further describe signatures of biased fractionation between sub genomes, as well as biases in functional annotations of genes retained as duplicated or single copy. This genome reference will act as an important resource for population-genomic analysis of the group and will aid our understanding of the rich history of allopolyploidy in the BCD clade and its evolutionary significance.

Analyses

Genome size estimation, genome assembly and transcriptome assembly

Using flow cytometry (FCM) (see methods) the haploid genome size of the accession COB1-7 was estimated to be 1.25 Gb (Giga base pairs). The genome assembly was sequenced using a method that combines assemblies from linked read sequencing (10X) with HiRise^{TM} scaffolding achieved through Chicago and Hi-C libraries constructed by Dovetail Genomics [33] (Table 1). As the final assembly had a L90 represented in 20 scaffolds (Table 1), we assumed that these scaffolds represented the haploid chromosomes of *B. decipiens* [34,35].

Table 1. Statistics of the *Bothriochloa decipiens* genome assembly

**Assembly statistics**	**10X**	**10X+Chicago**	**10X+Chicago+Hi-C**
**N50 (size/number)**	125 scaffolds / 2.733 Mb (mega base pairs)	116 scaffolds / 3.080 Mb	10 scaffolds / 53.95 Mb
**N90 (size(bp)/number)**	1,808 scaffolds / 0.023 Mb	616 scaffolds / 0.090 Mb	20 scaffolds / 42.637 Mb
**Largest scaffold size**	14.847 Mb	14.607 Mb	95.095 Mb
**Total number**	25,759	19,068	15,895
**Total genome size**	1,217.63 Mb	1,218.36 Mb	1,218.22 Mb
**% gaps**	6.78	6.84	6.86
**BUSCO[†] (*n*)**	39:200:13:3	43:200:9:3	45:200:8:2
**BUSCO[†] (%)**	15:78:5:1	16:78:3:1	17:78:3:0.8

[†]Number of BUSCO (Benchmarking Universal Single-Copy Orthologs) genes found in the assembly using the eukaryota odb9 dataset. Genes are split into four categories: complete and single-copy, complete and duplicated, fragmented, and missing, and reported respectively.

RNA (ribonucleic acid)- sequencing of two tissues (leaf and stem) was used to assemble the transcriptome of *B. decipiens* using Trinity v.2.8.5 (Trinity, RRID:SCR_013048) [36]. The final transcriptome assembly contained 197,655 transcripts, with 104,784 Trinity annotated genes, with an average length of 1,062 bp and a N50 length of 1,677.

Genome annotation, functional annotation, quality validation and repeat identification

We identified 60,652 putative protein coding genes (Supplementary Table S1) after running iterative runs of MAKER v.3.01.03 (MAKER, RRID:SCR_005309) [37] genome annotation pipeline that trained gene predictors AUGUSTUS v.3.3.3 (AUGUSTUS, RRID:SCR_008417) [38] and SNAP v.2013-11-29 [39]. Functional annotations for the predicted gene annotations were done by searching against several databases (see methods) (Table 2). We identified 94.1% of the core eukaryotic genes amongst our annotated genes, 22.7% being single copy, 71.4% being duplicated and 2.4% fragmented compared to BUSCO markers present in the library "eukaryota_odb10.2020-09.10". Of the total assembly length, 52.86% of the genome corresponded to repetitive elements (Supplementary Table S2) based on our custom repeat library constructed following recommendations of the MAKERP pipeline for advanced repeat construction [40] (see methods). The majority of the repeated elements were retrotransposons (36.09%) while only 3.34 % were DNA (Deoxyribonucleic Acid) transposons. The two most abundant retrotransposon families were Gypsy and Copia, representing 19.47% and 8.65% of the TEs (transposable elements) identified, respectively (Supplementary Table S2). Illustration of the *B. decipiens* genome landscape depicted how gene density was low towards the centre of each scaffold, where repeat density was high (Figure 1).

Table 2. Summary of functional annotation for protein coding genes.

**Database**	**Number of gene models with annotations**	**Percentage of gene models with annotations (%)**
UniProtKB/Swiss-Prot	42,417	69.93
Tair10	49,099	80.95
Pfam	43,444	71.62
KEGG (Kyoto Encyclopedia of Genes and Genomes) orthology	17,465	28.79
KEGG orthology - E.C (Enzyme Commission) number annotations	8302	13.68

Figure 1. The *Bothriochloa decipiens* genome landscape. Location across the 20 chromosomes (track A) and distribution, in 1Mb windows of gene density (track B), repeat density (track C), DNA-TE density (track D), LTR (long terminal repeats) -TE density (track E) and GC (guanine-cytosine) content (track F).

Genome synteny and whole genome duplication

In order to determine if *B. decipiens* had undergone a whole genome duplication, a reciprocal BLASTP (Basic Local Alignment Search Tool Program) (BLASTP, RRID:SCR_001010) [41] was conducted using *B. decipiens* protein sequences as the query against themselves and

homeologous scaffolds were identified using the collinear blocks obtained via MCScanX [42]. This was also corroborated by an alignment of the 20 largest scaffolds (> 40Mb; representing 1.11 Gb of the 1.22 Gb genome) against themselves using Minimap2 v.2.1.8 (Minimap2, RRID:SCR_018550) [43] (Supplementary Figure S1) . Of these 20 scaffolds, ten pairs (with more than 50% matching across both scaffolds) were identified as the pairs of homeologous scaffolds (Supplementary Figure S1). Similarly, we identified collinear blocks between the two putative sub genomes of *B. decipiens* and *Sorghum bicolor* (the closest sequenced diploid relative) by conducting a reciprocal BLASTP BLASTP, RRID:SCR_001010) [41] comparing protein sequences from each species using MCScanX [42]. We identified 33,146 *B. decipiens* genes that were orthologous to 19,611 *S. bicolor* genes across syntenic blocks. A relatively recent paleo polyploidization event was evident as each chromosome from *S. bicolor* almost completely aligned to a pair of *B. decipiens* scaffolds (Figure 2B). Further, these pairs of *B. decipiens* scaffolds show large syntenic blocks of duplicated genes (Figure 2A). There was also evidence of rearrangements between the sub genomes: for example, a translocation from scaffold 18 (homeologous to scaffold 10) to scaffold 8 which appears to have regions from both sub genomes as a result (Figure 2). Therefore, this translocation likely occurred after the allopolyploidization event. Translocations are apparent in the *B. decipiens* genome alignment against itself (Supplementary Figure S1) and also in the syntenic relationship between the *B. decipiens* chromosomes when all 20 of them are aligned against themselves (Supplementary Figure S2). Other structural changes can be observed, including several inversions, clearly identifiable on scaffold 13 when compared to scaffold 15 or to chromosome 6 in *S. bicolor* (Figure 2 (B)).

Figure 2 (A) The syntenic relationship between the pairs of homeologous chromosomes in *B. decipiens*. (B) The syntenic relationship between the *B. decipiens* and *S. bicolor* orthologous genes. Each *S. bicolor* chromosome (so) shares synteny with a pair of *B. decipiens* chromosomes (bd) suggesting that the allotetraploid *B. decipiens* genome was formed by the hybridization of two *Sorghum*-like ancestors.

Sub genome and homeologous exchange identification

The difference in the distribution of repetitive elements between pairs of homeologous chromosomes can provide evidence of sub genome ancestry [44]. Diagnostic repeat signatures are found on one of each homeologous pair of chromosomes, and they represent the remains of mobile elements with different activity in the diploid ancestors before the merging of the two genomes [44]. As there is no genomic data from any close diploid relatives that do not share the most recent allopolyploidization event, we clustered the putative homeologous chromosomes based on repeat abundance using kmer distributions. We partitioned the *B. decipiens* genome into sub genomes A and B by modifying the methods described in [45] (see methods). We found 919 13-mers (13-bp sequences) occurring at least 100 times across the whole genome and also were at least threefold-enriched in one of the homeologous pair relative to the other. Based on the consistent enrichment for these 919 13-mers along the putative homeologous chromosomes, each scaffold of a pair was assigned to a sub genome (Figure 3). The A group was defined based on the enhanced abundance of 773 13-mers, and the B group based on the enhanced abundance of the other 146 13-mers (Figure 3). We then computed the densities of A- and B-preferred 13-mers across the scaffolds (Supplementary Figure S3A&B) and identified potential homeologous exchange between sub genomes. Scaffold 8 from the B sub genome had a high density of sub genome A-preferred kmers at one end of the scaffold (Supplementary Figure S3B) consistent with the observation

of a translocation from the dot plots (Figure 2). We tested for homeologous exchange among the sub genomes using a Hidden Markov Model implemented (HMM) in the R\HMM package [46]. However, we did not find evidence of reciprocal homeologous exchange. Instances of assignment to the alternate sub genome by the HMM occurred in three regions (scaffold 9, 12, and 16), but these were regions of low kmer density, and therefore challenging to assign to sub genomes using this kmer based approach. Also, these instances did not reflect reciprocal exchanges between the sub genomes. Impacts of these ambiguities in sub genome assignment were examined by including and excluding these regions in downstream analyses involving sub genome identification (i.e., biased fractionation). We examined sub genome enriched LTRs (long terminal repeats), as differences in LTR activity in parental species can help differentiate sub genomes and can be used to assess the timing of allopolyploidy. We identified 255 LTR repeats that belonged to nine LTR families that were at least three times more common in one sub genome. These repeats also overlapped with A- or B-preferred kmers. There were eight LTR subfamilies (Grande1_ZM_pol/Gypsy, RIRE2_pol/Gypsy, Copia-11_SB/Copia, Copia-73_Mad/Copia, Copia-13_SB/Copia, SZ-7_pol/Gypsy, CRM/Gypsy, Atlantys_OS_polGypsy) identified in sub genome A and only one (Copia-9_SB/Copia) identified in sub genome B. Their genomic locations are shown in Supplementary Figure S3C&D.

Figure 3. The differentiation of homeologous pairs of chromosomes into sub genome A (blue) and sub genome B (red) based on the hierarchical clustering of Euclidean distances among scaffolds using counts of 13-mers.

The timeline of paleo tetraploidy

We used 1:1 orthologs identified using OrthoFinder v.2.3.8 (OrthoFinder, RRID:SCR_017118) [47] across members of the Andropogoneae tribe using *Panicum hallii* and *Setaria italica* as outgroups to identify the likely timing of the sub genome divergence in *B. decipiens* (see methods) (Figure 4A). We found that the diploid progenitors of the allopolyploid ancestor of *Bothriochloa decipiens* speciated approximately 5.8 MYA (Million years ago). Our tree also dated the divergence of the progenitors of *M. sinensis* to around 6 MYA.

Timing of sub genome specific LTR expansion

The greater abundance of LTR subfamilies specific to one sub genome is a signal of mobile element activity unique to one of the diploid ancestors of the allopolyploid [44]. Therefore, dating the insertion events of the sub genome specific LTRs (see methods) can provide a rough estimate of the time that the diploid ancestors of the allotetraploid *B. decipiens* existed independently before the hybridization event. We found that the sub genome specific LTR activity began 2-3 MYA and peaked around 3.5 MYA (Figure 4B). Activity declined around 6 MYA (Figure 4B), and this coincides with the timing of the allotetraploid speciation inferred through the phylogenetic analysis (Figure 4A). Overall, this suggests that the diploid ancestors may have evolved independently for about 3-4 MYA before the hybridization event.

Figure 4. (A) Phylogenetic tree (MYA) of the Andropogeneae showing the time (MYA) of divergence between the diploid ancestors of the allotetraploid *B. decipiens*. (B) Density

distribution of divergence the time estimate for all LTR families with sub genome-specific expansion activity.

Biases in gene retention between sub genomes

We analysed the collinear blocks between *S. bicolor* and *B. decipiens* to assess differences in retention of duplicated genes between sub genomes. Sub genome-specific retention was inferred as the number of genes retained in a given sub genome divided by the number of inferred ancestral (i.e., pre duplication) gene numbers. Collinear blocks obtained from the two independent scanning methods McScanX [42] and OrthoFinder v.2.3.8 (OrthoFinder, RRID:SCR_017118) [47] were used in two independent analyses to confirm any patterns (see methods). We then used a two-sided Fisher's exact test to determine if there was a significant difference in retention of genes between the sub genomes under the null hypothesis that gene loss between sub genomes was random. The percentage of genes retained was higher in sub genome A compared to sub genome B (McScanX, Fisher's exact test, *P*-value = 2.2 x 10^{-16} and OrthoFinder, Fisher's exact test, *P*-value = 1.63 x 10^{-10} (Table 3).

Table 3. Sub genome specific gene retention as observed in analysis with McScanX and OrthoFinder

**Clustering Method**	**Ancestral genes (retained +**	**Ancestral genes retain**	**Ancestral genes retain**	**Ancestral genes retain**	**Percent retained**	**Percent retained**	**Percent retained on**

	**single)**	**ed on A**	**ed on B**	**ed on A+B**	**on A (%)**	**on B (%)**	**A+B (%)**
McScanX	19,523	16,666	15,464	12,195	85	79	62
OrthoFinder	15,262	12,716	12,278	9,620	83	80	53

Differences in the function of duplicated genes retained after the WGD compared to those returning to single copy was tested through gene enrichment analysis using the R\topGo package (topGO, RRID:SCR_014798) [48]. All single-copy genes in A or B, or duplicated genes in both A and B were used as foreground genes and the remaining ancestral genes (retained duplicated or single copy) as background genes. GO (Gene ontology) terms related to organelle functions such as chloroplast organisation, chloroplast RNA modification and regulation of mitochondrion organisation were among the top 10 most significant terms associated with genes returning to single copy (Figure 5A, Supplementary Table S3). Among the ten most significant terms for genes maintained as duplicates were those relating to transcription, including positive and negative regulation of transcription, and terms related to external stress response such as response to water deprivation and salt stress (Figure 5B and Supplementary Table S4).

Figure 5. Top 30 GO terms over-represented in (A) genes retained as single-copy and (B) genes retained as duplicates following the paleo tetraploidy event.

Discussion

Here we report the chromosome-level genome assembly of *Bothriochloa decipiens*, a native Australian grass species important in grassland rehabilitation. Our comparative analysis revealed that this species is a diploidized allotetraploid, consistent with previous phylogenetic analysis of the group [28]. Our assembly and comparative analysis revealed a relatively recent whole genome duplication. Although the diploid progenitors are unknown, our clustering based on unique repeat signatures grouped the chromosomes into sub genomes. Phylogenetic analysis revealed how the diploid progenitors of the allopolyploid ancestor of *Bothriochloa decipiens* speciated approximately 5.8 MYA. Additionally, we showed evidence of biased fractionation with significantly higher gene retention from one of the sub genomes. This sub genome also appeared to have more active LTRs just prior to the allopolyploidy event. Consistent with hypotheses, genes that were retained as duplicated following the WGD event were enriched for functions involving transcription and stress response.

Patterns of gene loss and retention following allopolyploidy

The two sub-genomes show asymmetric gene loss where sub genome A retained more genes than sub genome B (Table 3). This ‘biased fractionation’ commonly occurs after allopolyploidization [49] and has been observed in *Arabidopsis* [21], maize [22,50], and *Brassica* [19], although it is not always observed [51]. Biased fractionation can be a result of genome dominance, where gene expression tends to be higher in one sub genome compared

to the other, leading to greater gene loss in the sub genome with reduced expression [19,20,23]. Interestingly, sub genome A also appears to have had more active LTRs at the time of the most recent allopolyploidy event, as evidenced by the greater number of diagnostic kmers and LTR families associated with sub genome A (Supplementary Figure S3). DNA methylation is known for its role in epigenetic gene silencing [52–54] and in restricting TE activity [55]. It is possible that higher LTR activity prior to the WGD (Figure 4B) and the dominance of sub genome A (Table 3) are related, and in fact caused by greater genome-wide silencing in sub genome B. In support of this theory, more retained genes and more active TEs have also been observed in the dominant sub genome of *Miscanthus* [45], but further investigation is warranted.

Functions overrepresented among the genes retained as duplicates, and the genes retained as single copy in the two sub genomes are aligned with theories as well as a large body of empirical work [56–60]. Genes containing domains responsible for functions such as RNA modifications and transmembrane activity tend to revert to singletons in plants [59]. Congruently, we identified genes related to RNA modification and transmembrane activity in our GO enrichment analysis of single copy genes (Figure 5A). We also identified enriched GO terms related to organelles such as chloroplast, mitochondria and endoplasmic reticulum in the single-copy gene set. Genes encoding functions related to organelles are commonly retained as single copies [57]. An alteration of the dosage balance could explain the retention of single copy genes responsible for organelle-mediated processes such as photosynthesis in chloroplasts and respiration in mitochondria. These functions involve proteins from both the organelle and nuclear genome. The interactions between the two genomes are tightly regulated to maintain the balance of the protein products created from the separate genomes [61,62]. During a WGD, this balance could get affected as only the nuclear genome is

duplicated, not the organelle's [14]. Alternatively, biased fractionation may reduce mixing of genes from the two diploid progenitors [63]. Nuclear encoded genes performing organelle functions and organelle genes have coevolved in each ancestral genome separately, and biased gene loss and reversion to single copy might maintain coadapted gene complexes and prevent negative interactions of genes between the sub genomes [63].

Alongside genes reverting to single copies, many genes were retained as duplicates. Genes coding for subunits of multimeric proteins or complexes, transcription factors and signal transduction mechanisms are biased to avoid fractionation [56,58,60]. GO terms related to transcription, signal transduction and protein biosynthesis were over-represented amongst retained duplicate genes (Figure 5B). The gene dosage hypothesis explains these patterns of biased retention [16]: if either gene copy that codes for a dosage-sensitive gene product that interacts with other gene products is lost, the dosage imbalance may be deleterious to the organism. We also found that retained duplicated genes had an overrepresentation of GO terms related to response to external stressors like water deprivation, salt stress and response to abscisic acid signalling pathways (Figure 5B). Genes that give plants the ability to respond to various environmental stresses are frequently retained as duplicates after WGD [64]. As neofunctionalization and specialisation can be possible fates of genes retained as duplicates, retention of such genes may promote adaptive evolution to abiotic stresses [65–68]. Multiple paleo polyploidization events that occurred independently throughout the history of evolution of angiosperms could have promoted the diversification of angiosperms across a wide range of environmental conditions by contributing to adaptation to new environments and stress [69].

The evolutionary history of the Andropogenae

Species in the Andropogenae clade, which *B. decipiens* belongs to, are dominant in modern day C4 grasslands. Most allopolyploid events in the Andropogenae occurred recently, in the late Miocene period [28], which coincides with the expansion of C4 grasslands [70]. Our phylogenetic analysis suggests the speciation event leading to the diploid ancestral genomes occurred at the end of the Miocene, approximately 5.8 MYA, which corresponds to other estimates reported for *Bothriochloa spp*. [28]. It also appears to have occurred at a similar time as the speciation event leading to the *Miscanthus* A and B sub genomes [45]. For *B. decipiens* the insertion times of the sub genome-specific LTRs suggest that these species were diverged for up to 4 million years before the hybridization event, although the date estimates are prone to error due to substitution rate variation among LTR families [71].

The genus *Bothriochloa* belongs to a group known as the BCD clade along with the two other genera, *Capillipedium* and *Dichanthium [34]*. Species in this clade are able to interbreed even though they are morphologically diverged. For instance, *Bothriochloa bladhii* has been identified as a compliospecies, able to absorb genomes from different species in the BCD complex [28,34]. Interestingly, a phylogenetic study of species in the BCD clade in Australia suggested that *B. decipiens* may be an ancestral diploid species of the BCD clade, making *B. decipiens* a key species to further our understanding of the evolution of this group [32]. Our high-quality reference genome should spur future comparative genomic studies of allopolyploidy and hybridization in this clade and in grasses more generally.

Most allopolyploidization events in the Andropogenae are recent, such as the one we report in *B. decipiens*. These events are concurrent with the time of the expansion of C4 grasslands, and some studies argued that the allopolyploidy gave these grasses the ability to adapt to new environments, thereby enabling successful expansion and establishment [72,73]. The biased

retention of duplicated genes related to stress response provides support for this hypothesis, yet the correlation between C4 grassland expansion and recent allopolyploidization may or may be causal and requires further study. Future analysis examining the adaptive significance of retained duplicates using both comparative and population genomic approaches across a greater number of taxa, including groups with frequent WGD such as the Andropogenae, will further our understanding of the adaptive significance of allopolyploidy and its potential role in niche expansion in C4 grasses.

Potential implications

This genome will be an important resource for population genomic studies involving native grasses in this genus. Such analyses will shed light on the adaptive genetic landscape of these important foundation species, which could play a critical role in the development of climate change resilient grassland restoration practices in Australia and elsewhere [74,75]. Further this genome will be important in broader comparative analyses of the Andropogenae which should provide greater insight into the evolutionary significance of allopolyploidy.

Methods

Species description

Bothriochloa decipiens (blue pitted grass) is a warm season, perennial, tufted grass that can grow up to 1m in height [76]. Due to its ability to establish well from direct seeding on many soil types, and the ability to withstand pressure caused by overgrazing, it has become an important species for rehabilitation. It is widespread in subtropical New South Wales (NSW) and Queensland as well as tropical Queensland [77]. It is a close relative, and phenotypically similar to, the polyploid *Bothriochloa macra,* which is a widespread native grass species in

south-eastern Australia. The diploid chromosome number of *B. decipiens* is reported to be 2n=40 [35].

Sample collection

The seeds used to grow the diploid *B. decipiens* accession COB1-7 used in this study were collected from Cobbitty, NSW (34°03’N, 150°68’E). Using these seeds, a plant was grown and maintained at Monash University, Clayton to obtain leaf and inflorescence tissue samples for DNA and RNA extractions for the study.

Flow cytometry

We used FCM to estimate the genome size and predict the relative ploidy of 24 populations of *Bothriochloa macra* and *Bothriochloa decipiens*, and also sought evidence for within-population variation in ploidy. We estimated the ploidy of at least 5 plants from each population following a modified plant FCM protocol [78]. Leaf samples from each population were collected from greenhouse grown plants and immediately placed on ice for same-day cytometric analysis. Three DNA genome size standards were selected, *Solanum lycopersicum* (2C = 1.96) and *Pisum sativum* (2C = 9.09), and grown from seed. Approximately 40 mg (milligram) of fresh leaf material was used for each sample, and placed into a 2.0 mL (millilitre) tube with a single 3 mm (millimetre) tungsten carbide bead and 300 µL (microliters) of an ice-cold nuclei suspension buffer modified from de Laats buffer (1984): 15mM (millimolar) HEPES, 1mM EDTA, 0.2% (v/v) Triton X-100, 80mM KCl, 20mM NaCl, 300 mM sucrose, 0.5 mM spermine, 15mM β-mercaptoethanol, 0.25 mM PVP-40. Adjusted to pH7. Samples were placed in a Qiagen Tissuelyser II and ground for 24

seconds at 25 hertz, and then the sample rack was reversed and ground again. The homogenate was filtered through two layers of Millipore Miracloth (22-25 µm (micrometre) pore size) suspended in a 3-piece nozzle. One µL of 10 µg (microgram)/µL RNAse was added for every 100 µL of filtrate and incubated at 37℃ for 20 minutes. Fifteen µL of 0.1 µg/ µL of Propodium Iodide station solution was added to the filtrate and samples were run on the BD Accuri™ C6 Cytometer using the settings outlined in [79]. Internal standards were run on the cytometer at the beginning and end of the session – no change in dye fluorescence was recorded over that period of time.

A total of 38 samples produced an observable signal in the FCM run. All samples, excluding standards, were run in a blind fashion so that prior knowledge of expected ploidy did not bias the identification of nuclei peaks. The 2C values were determined for all *Bothriochloa* samples by comparing the FL2-A value of the sample to the internal standards, *Solanum* and *Pisum*, which have a known 2C value of 1.96 and 9.09pg respectively [78]. The average 2C genome size of diploid and polyploid plants was 2.80 pg (range 2.56 –2.99 pg) and 5.38 pg (range 4.94 – 5.91 pg), respectively (Supplementary Figure S4). Only one population (COB1) consisted of diploid individuals, and all individuals from this population were tested to confirm our findings. The COB1-7 accession was 2.56 pg which leads to a haploid genome size estimate of 1.25 Gb (Supplementary Figure S5). The polyploid samples were likely closely related and phenotypically similar *B. macra*.

DNA extractions

For DNA extraction, fresh leaf tissue was collected from diploid individual COB1-7, flash frozen in liquid nitrogen and stored at -80 ℃. The tissue was then shipped to Dovetail Genomics who completed the DNA extractions. To obtain high molecular weight DNA for

10X Genomics linked read sequencing, 1.8g of leaf material was ground with mortar and pestle to a fine powder to which 200mL of prewarmed CTAB and 100µL BME was added. This was incubated at 68℃ for 15 minutes. Once incubated, a mixture of 2x phenol chloroform, 1x isoamyl and 0.7x isopropanol was added and centrifuged to form a pellet. The pellet was combined with 9.5 mL of G2, 200µL protease and 19µL RNase. Again the mixture was incubated at 50℃ for 1 hour. The precipitated genomic DNA was used in library constructions.

10X Library preparation sequencing and 10X assembly

Genomic DNA (gDNA) with an adjusted concentration between 1.0 -1.25 ng/µL was used to prepare the whole genome sequencing libraries using the Chromium Genome Library and Gel Bead Kit v.2, Chromium Genome Chip Kit v.2, Chromium i7 Multiplex Kit and Chromium controller according to manufacturer's instructions (10X Genomics). Genomic DNA was combined with Master Mix, a library of Genome Gel Beads, and partitioning oil to create Gel Bead-in-Emulsions (GEMs) on a Chromium Genome Chip. The GEMs were isothermally amplified with primers containing an Illumina Read 1 sequencing primer, a unique 16bp (base pairs) 10X barcode and a 6bp random primer sequence. Bar-coded DNA fragments were recovered for Illumina library construction. The amount and fragment size of post-GEM DNA was quantified prior using a Bioanalyzer 2100 with an Agilent High sensitivity DNA kit. Prior to Illumina library construction, the GEM amplification product was sheared on an E220 Focused Ultrasonicator (Covaris, Woburn, MA) to approximately 350bp. Then, the sheared GEMs were converted to a sequencing library following the 10X standard operating procedure. The library was quantified by qPCR (quantitative polymerase chain reaction) with a Kapa Library Quant kit (Kapa Biosystems-Roche) and sequenced on a NovaSeq6000 sequencer (Illumina, San Diego, CA) with paired-end 150bp reads.

Chicago library preparation and sequencing

A Chicago library was prepared as described in [33]. Briefly, ~500ng of high molecular weight gDNA was reconstituted into chromatin *in vitro* and fixed with formaldehyde. Fixed chromatin was digested with DpnII, the 5’ overhangs filled in with biotinylated nucleotides, and then free blunt ends were ligated. After ligation, crosslinks were reversed and the DNA purified from protein. Purified DNA was treated to remove biotin that was not internal to ligated fragments. The DNA was then sheared to ~350bp fragments and sequencing libraries were generated using NEBNext Ultra II kit with Illumina-compatible indices. Biotin-containing fragments were isolated using streptavidin beads before PCR enrichment of each library. The libraries were sequenced on an Illumina HiSeq X to produce 467 million 2x150bp paired end reads.

Dovetail HiC library preparation and sequencing

A Dovetail HiC library was prepared as described in [80]. Briefly, for each library, formaldehyde was used to fix chromatin in the nucleus in place. Fixed chromatin was digested with DpnII, the 5’ overhangs filled in with biotinylated nucleotides, and then free blunt ends were ligated. After ligation, crosslinks were reversed and the DNA purified. Purified DNA was treated to remove biotin that was not internal to ligated fragments. The DNA was then sheared to ~350bp mean fragment size and sequencing libraries were generated using NEBNext Ultra enzymes and Illumina-compatible adapters. Biotin-containing fragments were isolated using streptavidin beads before PCR enrichment of each library. The libraries were sequenced on an Illumina HiSeq X to produce 400 million 2x150bp paired end reads.

Genome assembly

The 10X sequence data were assembled *de novo* with Supernova (Supernova assembler, RRID:SCR_016756) [[81]. This *de novo* assembly, along with Chicago library reads and Dovetail HiC library reads, was used as input data for HiRise, a software pipeline designed specifically for using proximity ligation data to scaffold genome assemblies [33]. An iterative analysis was conducted. First, Chicago library sequences were aligned to the draft *de novo* assembly from Supernova using SNAP [82]. The separations of Chicago read pairs mapped to the draft scaffolds were analyzed by HiRise to estimate the genomic distance between read pairs, and the model was used to identify and break putative misjoins, score prospective joins, and make joins above a threshold. After aligning and scaffolding Chicago data, Dovetail HiC library sequences were aligned and scaffolded following the same method. After scaffolding using Chicago and HiC library data, visual inspection of the contact maps identified two misjoin events in the largest scaffold and one other scaffold (black circles in Supplementary Figure S6). Manual corrections were performed using link density plots in Juicebox (Juicebox, RRID:SCR_021172) [83] to make breaks within those two scaffolds and produce the link density plot for the final assembly (Supplementary Figure S7).

mRNA-seq library preparation

RNA was extracted separately from young (a few weeks) and old (a year) tissue (leaf and stem) from one individual using the Qiagen RNeasy kit. RNA was pooled and a library was synthesised and sequenced by Genewiz^{TM} on an Illumina Novaseq 6000 platform in 2x150bp mode, resulting in 64,756,621 reads.

Transcriptome assembly

Raw RNA-seq reads were first cleaned by trimming the adapters using Trimmomatic v. 0.38 (Trimmomatic, RRID:SCR_011848) with the parameter "ILLUMINACLIP:TruSeq3-PE.fa:2:30:10:2:keepBothReads LEADING:3 TRAILING:3 MINLEN:36" [84]. The trimmed reads were used to assemble the transcriptome using Trinity v.2.8.5 (Trinity, RRID:SCR_013048) [36].

Annotation of repetitive sequences

A custom repeat library was constructed following recommendations of the MAKERP pipeline for advanced repeat construction [40]. Both structure-based and homology-based approaches were used to increase the power to detect repeats. Sequences of miniature inverted repeat transposable elements (MITEs) were collected using MITE-Hunter (Mite-Hunter, RRID:SCR_020946) [85] using all the default parameters. Long terminal repeat retrotransposons (LTRs) were collected using LTRharvest (LTRharvest, RRID:SCR_018970) and LTR-digest [86,87]. The candidates were filtered for false positives caused by other tandem repeats such as centromeres, tandem gene duplications or other transposable elements by identifying those sequences whose alignments extend beyond the LTR boundary. Representative sequences (exemplars) were chosen as described previously [50] to reduce the redundancy of the LTR. Then other repetitive elements were collected by first masking the genome sequence with the previously obtained MITE and LTR sequences. Then unmasked sequences were extracted and processed by RepeatModeler v.2.0.3 (RepeatModeler, RRID:SCR_015027) [88] to identify additional repeats. As many repeats carry gene fragments, all the collected repetitive elements were searched against a plant protein database that contains those from swissprot plant protein and NCBI (National Centre for Biotechnology Information) Refseq plants [89] with transposon proteins excluded. Elements with significant hits to genes were removed along with 50bp upstream and downstream of the

hit. If the remaining sequence was less than 50bp then it was completely excluded. Sequences matching the plant proteins as well as 50bp of flanking sequences were removed using the package ProtExcluder [40]. After this if the remaining portion of the sequence was shorter than 50bp, the entire sequence was excluded. Sequences of all the identified repetitive elements were joined together to form a final custom repeat library to be used to mask the repetitive elements of the genome in the Maker genome annotation protocol [37].To identify the type of repeat (including the repeat family), the unidentified repeats from RepeatModeler, as well as the LTRs and MITES from the custom library were searched against two transposase databases. The first was Tpases020812 [90]. This database is composed of transposase protein sequences from the RepeatMasker v.4.1.1 (RepeatMasker, RRID:SCR_012954) [91], and from two other sources [92,93], and was searched using BLASTX (BLASTX, RRID:SCR_001653) [94]. The second was the publicly available Dfam-curated library of repeats [95], which was searched using Hmmer v.3.3.1 (Hmmer, RRID:SCR_005305) [96] implemented through [97]. The sequences that matched the database were classified according to their top hits. Using this custom repeat library, RepeatMasker version 4.1.1 (RepeatMasker, RRID:SCR_012954) [91] was used to mask the genome, and identify the distribution of repeat types.

Genome annotation

MAKER v.3.01.03 (MAKER, RRID:SCR_005309) [37] genome annotation pipeline was used to annotate the genome. The input files provided for the first run were the genome assembly fasta file (Genbank accession JALGXP000000000), the reference transcriptome assembly fasta file (obtained from Trinity), and the protein homology evidence from a plant protein database [89] which combines the Swissprot plant protein database and NCBI Refseq for plants excluding transposable elements. Repetitive regions were masked using our custom

repeat library. Additional regions with low complexity were soft masked using RepeatMasker v.4.1.1 (RepeatMasker, RRID:SCR_012954) [91]. Iterative runs of MAKER v.3.01.03 (MAKER, RRID:SCR_005309) [37] were undertaken in order to train the gene predictors SNAP v.2013-11-29 [39] and AUGUSTUS v.3.3.3 (AUGUSTUS, RRID:SCR_008417) [38] as recommended by [37]. The first round of annotation was based on alignments of the transcriptome to the genome. For the first round the est2genome option in the Maker control file was set to 1 to allow Maker to infer gene models directly from the RNA-seq evidence in the transcriptome. After the completion of the first round of annotations, gene models with an AED (Annotation Edit Distance) score of 0.25 or greater and a length of 50 or more amino acids were retained and used to train SNAP v.2013-11-29 [39] to obtain a SNAP hmm file. We then trained AUGUSTUS v.3.3.3 (AUGUSTUS, RRID:SCR_008417) [38] using BUSCO v.3.0.2 (BUSCO, RRID:SCR_015008) [98]. First, training sequences were identified using the gene models predicted by Maker from the first run by excising regions with mRNA annotations and 1000 bp on either side. These were used to run BUSCO using the embryophyte set of conserved genes and an initial hmm model from rice. After training both SNAP and Augustus, Maker was run again, with SNAP hmm and Augustus files. A total of three rounds of training for each gene predictor were run. We used the script genestats [99] to calculate the numbers and lengths of genes, exons, introns and UTR (untranslated region) sequences present in the predicted gene models by the final Maker run (Supplementary Table S1). We ran BUSCO v.5.1.3 (BUSCO, RRID:SCR_015008) [100] with the eukaryota_odb10 lineage data set on the predicted transcript fasta file by Maker to assess the quality and the completeness of the annotated genome.

Sub genome and homeologous exchange identification

First, we identified the 20 largest scaffolds (> 40Mb; representing 1.11 Gb of the 1.22 Gb genome). These scaffolds were then aligned against themselves using Minimap2 v.2.1.8 (Minimap2, RRID:SCR_018550) [43] to identify scaffolds that shared homology and synteny that would indicate putative homeologous chromosomes. The alignments were plotted using the R\pafr package v.0.0.2 [101] . Of these 20 scaffolds, ten pairs (with more than 50% matching across both scaffolds) were identified as the pairs of homeologous scaffolds (Supplementary Figure S1). As there is no genomic data from any close diploid relatives that do not share the most recent allopolyploidization event, we clustered the putative homeologous chromosomes based on repeat abundance using kmer distributions. We partitioned the *B. decipiens* genome into sub genomes A and B by modifying the methods described in [45]. Specifically, we first identified 13 base pair sequences (13-mers) using Jellyfish v. 2.3.0 (Jellyfish, RRID:SCR_005491) [102] and retained kmers at high abundance in the assembly (100 x or above). For each pair of scaffolds, we compared the counts of these 13-mers, identifying those that differed in abundance by three-fold or more between scaffolds. To control for any differences in scaffold length impacting this assessment, we further reduced the set of diagnostic 13-mers to those that retained a three-fold difference after standardising kmer count for the scaffold length, while keeping only those diverging in the same direction as the absolute kmer count. Hierarchical clustering of scaffolds based on difference in 13-mer counts was used to identify putative sub genomes as implemented in the R\ComplexHeatmaps package (ComplexHeatmaps, RRID:SCR_017270) [103].

We tested for homeologous exchange among the sub genomes using a Hidden Markov Model implemented in the R\HMM package [46] . We used the most common kmer type (A or B) in 1Mbp windows as the observed states and the sub genome type for each of the 1,121 windows. The initial HMM used equal starting probabilities and transition probabilities of

0.01. We trained the HMM emission probabilities (viterbiTraining) using Scaffold 5 and Scaffold 15 as they appeared not to be subject to any sub genome exchange based on the A and B kmer density plots (Supplementary Figure S3).

We examined sub genome enriched LTRs, as differences in LTR activity in parental species can help differentiate sub genomes and can be used to assess the timing of allopolyploidy. LTRs were used for this because they are rapidly evolving, making it easy to differentiate between related sub families. The timing of insertions can be calculated by examining the substitution rates for members of the same subfamily using the 5’ and 3’ regions [45]. Specifically, intact retrotransposons in the genome were identified using LTR-HARVEST [86]. The ‘best’ option was used for pairing overlapping LTR sequences, allowing the inner sequences of the retrotransposons to contain gaps. We performed an all-versus-all BLAST (BLAST, RRID:SCR_004870) [41] on the long terminal repeat segments of the identified LTRs with an e-value cut off of 1e-2. Hits with the percentage of alignment between query and subject equal or greater than 90% over their entire length were selected. We then used MCL algorithm [104] to cluster the filtered blast alignments into retrotransposon subfamilies using an inflation parameter (-I) of 3. We counted the occurrence and the total base pairs that each LTR subfamily obtained from above clustering in the putative A and B sub genomes identified above. We identified LTR subfamilies that were three times more common in one of the sub genomes using both occurrence and bp count. Then we determined if these repeats overlapped with multiple A or B genome preferred kmers to confirm that kmers were representing longer repetitive sequences and to confirm that these kmers were marking repeat expansion that occurred just before the allopolyploidy event.

Gene function prediction

The predicted protein sequences obtained from the final run of MAKER were aligned to the UniProtKB/Swiss-Prot [105] and TAIR10 [106] protein databases using BLASTP (BLASTP, RRID:SCR_001010) [41] with an e-value cut-off of 1.0e-5. The GO term associated with the best hit for each BLASTP search was identified in each of the three databases above and assigned to the *B. decipiens* query. InterProScan v. 5.51-85.0 (InterProScan, RRID:SCR_005829) [107] was used to search the query protein fasta against the Pfam [108] protein family database and identify functional protein domains. Pfam accessions and GO terms were retrieved for the *B. decipiens* query sequences. The query protein sequences were BLAST (Basic Local Alignment Search Tool) searched against the KEGG database [109–111] using the online tool KofamKOALA - KEGG orthology search [112] with an e-value cut-off of 1.0e-5. KEGG orthology terms and enzyme codes were retrieved for each hit.

Genome synteny and whole genome duplication

To determine if the *B. decipiens* genome had undergone whole genome duplication, a reciprocal BLASTP (BLASTP, RRID:SCR_001010) was conducted using *B. decipiens* protein sequences as the query against themselves with a minimum e-value greater than le-5. Then MCScanX [42] was used to identify syntenic blocks within the genome. The collinear blocks obtained via MCScanx between the putative A and the B sub genome of *B. decipiens* were visualised using Synvisio [113]. Similarly, we identified collinear blocks between the two putative sub genomes of *B. decipiens* and *S. bicolor* by conducting a reciprocal BLASTP (BLASTP, RRID:SCR_001010) comparing protein sequences from each species using MCScanX and plotted the results using Synvisio.

Estimating the timeline of paleo tetraploidy

We estimated the timing of speciation events in the Andropogoneae using *Panicum hallii* and *Setaria italica* as outgroups. The reference gene sets for *Sorghum bicolor* v3.1.1, *Panicum hallii* v2.2, *Setaria italica* v2.1, *M. sinensis* v7.1 and *Zea mays* (B73 RefGen_v4) were downloaded from Phytozome v12.1 (Phytozome, RRID:SCR_006507) [114]. The *Saccharum spontaneum* reference gene set [115] was also downloaded. We separated the A and B sub genomes of *M. sinensis* (as identified in [45]), as well as those of *B. decipiens*, to compare the timeline of the paleo allopolyploidy events between *Miscanthus* and *B. decipiens* species. We identified 1:1 orthologs between all species (or sub genomes) using OrthoFinder v.2.3.8 (OrthoFinder, RRID:SCR_017118) [47]. A core set of 392 single-copy genes were retained and multiple sequence alignments were performed for each orthologous cluster using Genodup [116] and Mafft (Mafft, RRID:SCR_011811) [117]. Poorly aligned regions were removed using Gblocks 0.91b (Gblocks, RRID:SCR_015945) [118] and the final alignments were concatenated into a single alignment.

Phylogenomic analysis

The best model of evolution was inferred using jModelTest2 [119,120]. A phylogenetic tree was constructed using RAxML (RAxML, RRID:SCR_006086) [121] with the GTRGAMMAI model of evolution and 1000 bootstrap replicates. *Setaria italica* and *Panicum hallii* were designated as outgroups.

Divergence date estimation

Divergence among lineages in the phylogeny were estimated from the concatenated alignment using BEAST v.2.5 (BEAST, RRID:SCR_010228) [122,123] after using bModelTest [124] to infer the best substitution model. Parameters included the GTR (general time reversible) substitution model with unequal frequencies, four gamma categories,

estimated shape and invariant sites. We chose a relaxed log normal clock with estimated clock rates. We set priors to constrain the estimated dates at the *Setaria-Panicum* (12.8-20 MYA), and the Andropogeoneae (13-21.2 MYA) nodes, using a uniform distribution between the minimum age and maximum ages of divergence times obtained from the TimeTree database [125]. BEAST2 (BEAST2, RRID:SCR_017307) [122] analysis was conducted for 50 million generations, and logging at every 5000 trees. Convergence between runs was assessed with Tracer v.1.6 [126]. Trees were summarised with TreeAnnotator [123] using a burn-in value of 20%.

Timing of sub genome specific LTR expansions

We aligned the long terminal repeats of each LTR family cluster using Mafft (Mafft, RRID:SCR_011811) [117]. We computed Jukes-Cantor distance matrices using the R\ape package [127]. We estimated the divergence times of each LTR family as k/2r (k=divergence, r=substitution rate) [128]. We used 1.3 x 10^{-8} as the substitution rate per site per year [129].

Determination of biases in sub genome gene retention

We analysed the collinear blocks between *S. bicolor* and *B. decipiens* to assess differences in retention of duplicated genes between sub genomes. Sub genome-specific retention was inferred as the number of genes retained in a given sub genome divided by the number of inferred ancestral (i.e., pre duplication) gene numbers. Consequently, we calculated the number of ancestral (pre duplication) genes as those orthologous genes present in *S. bicolor* and in one or both of the two subgenomes. We then compared this number to the total number of genes present only in sub genome A, only in sub genome B or in both subgenomes. We then used a two-sided Fisher's exact test to determine if there was a

significant difference in retention of genes between the subgenomes under the null hypothesis that gene loss between subgenomes was random. We also used the results from OrthoFinder (OrthoFinder, RRID:SCR_017118) to confirm this pattern. Specifically, we identified 1:1 and 1:2 orthologs between *S. bicolor* and *B. decipiens*, only retaining 1:2 orthologs that were mapped to chromosomes on both *B. decipiens* subgenomes.

Differences in the function of duplicated genes retained after the WGD compared to those returning to single copy was tested through gene enrichment analysis using R\topGo (topGO, RRID:SCR_014798) [48]. All single-copy genes in A or B, or duplicated genes in both A and B were used as foreground genes and the remaining ancestral genes (retained duplicated or single copy) as background genes.

Availability of source code

R scripts used in this study can be found in the public github repository [130].

Data Availability

Raw reads for the genome assembly have been deposited under BioProject accession number PRJNA819081. Illumina library raw reads namely 10x, Chicago and HiC data have been deposited in the Sequence Read Archive (SRA) under study accession numbers SRR18458736, SRR18471564 and SRR18471563. RNAseq data have been deposited under SRA accession number SRR18471562. Genome assembly of *B. decipeins* is deposited in the NCBI genome database under the accession JALGXP000000000.

Abbreviations

℃: degree Celsius, BCD : *Bothriochloa Capillipedium* and *Dichanthium*, BLAST : Basic Local Alignment Search Tool, BLASTP :Basic Local Alignment Search Tool Program, bp : base pairs, BUSCO : Benchmarking Universal Single-Copy Orthologs, DNA: Deoxyribonucleic Acid, FCM :flow cytometry, Gb : Giga bases, gDNA :genomic DNA, GO : Gene ontology, HMM :Hidden Markov Model, KEGG : Kyoto Encyclopedia of Genes and Genomes, LTR : long terminal repeats, Mb : mega base pairs, mg :milligram, MITE : miniature inverted repeat transposable element, mL :millilitre, mM : millimolar, mm : Millimetre, mRNA : Messenger RNA, MYA : Million Years Ago, NCBI : National Centre for Biotechnology Information, ng : nanogram, NSW : New South Wales, PCR : polymerase chain reaction, RNA : ribonucleic acid, Seq :sequencing, TE : transposable elements, UTR : untranslated region, WGD : Whole genome duplication, µg : microgram, µL : microliters, µm : micrometre

Competing Interests

The authors declare that they have no competing interests.

Funding

This study was supported by Hermon Slade Foundation - Grant number HSF1703, Monash Graduate Scholarship, Monash University, Monash International Tuition Scholarship, Monash University and Denis and Maisie Carr Award and Travel grant 2020, School of Biological Sciences, Monash University.

Author’s Contributions

K. A. H. - conceived and designed the study. K.A.H., J.L.M. and A.F.L. - supervised the research, C.L. conducted the FCM, RNA extractions and phylogenetic analyses, K.A.H. and N.P.D. - annotated the genome, carried out the sub genome and homeologous exchange identification, genome synteny and whole genome duplication analysis and timing of sub genome-specific LTR expansion, N.P.D. carried out repeat library construction, transcriptome assembly, N.P.D. and P.B. carried out functional predictions, determination of biases in sub genome gene retention. N.P.D and K.A.H. wrote the first draft of the manuscript. All authors approved the final version of the manuscript.

Acknowledgements

We thank Brendon Levot for collecting the seeds from which the individual specimen used in this study was grown from. Extraction of genomic DNA, preparation and sequencing of libraries and genome assembly was done by Dovetail Genomics. The preparation and sequencing of RNA-seq libraries was done by Genewiz. Permission to collect plant material for the study was granted by NSW National Park and Wildlife Service - Licence number- SL 102026.

References

1. Grant V. Plant Speciation. 1981.

2. Ledyard Stebbins G. Variation and evolution in plants. Columbia University Press.1950.

3. Edger PP, Heidel-Fischer HM, Bekaert M, Rota J, Glöckner G, Platts AE, et al.. The butterfly plant arms-race escalated by gene and genome duplications. *Proc Natl Acad Sci U S A*. 2015;112:8362–8366.

4. McCarthy EW, Chase MW, Knapp S, Litt A, Leitch AR, Le Comber SC. Transgressive phenotypes and generalist pollination in the floral evolution of Nicotiana polyploids. *Nat Plants*. 2016; 2:16119.

5. Stebbins GL. Processes of organic evolution. Second Edition. Prentice-Hall.1971.

6. Soltis PS, Soltis DE. Ancient WGD events as drivers of key innovations in angiosperms. *Curr Opin Plant Biol*. 2016;30:159–165.

7. Doyle JJ, Flagel LE, Paterson AH, Rapp RA, Soltis DE, Soltis PS, et al.. Evolutionary genetics of genome merger and doubling in plants. *Annu Rev Genet*. 2008; 42:443–461.

8. Freeling M. Bias in plant gene content following different sorts of duplication: tandem, whole-genome, segmental, or by transposition. *Annu Rev Plant Biol*. 2009; 60:433–453.

9. Lim KY, Soltis DE, Soltis PS, Tate J, Matyasek R, Srubarova H, et al.. Rapid chromosome evolution in recently formed polyploids in Tragopogon (Asteraceae). *PLoS One*. 2008; 3:e3353.

10. Mandáková T, Joly S, Krzywinski M, Mummenhoff K, Lysak MA. Fast diploidization in close mesopolyploid relatives of *Arabidopsis*. The Plant Cell. 2010;*22*(7), 2277–2290.

11. Tayalé A, Parisod C. Natural pathways to polyploidy in plants and consequences for genome re organization. *Cytogenet Genome Res*. 2013;140:79–96.

12. Sémon M, Wolfe KH. Consequences of genome duplication. *Curr Opin Genet Dev*. 2007;17:505–512.

13. Conant GC. Comparative genomics as a time machine: how relative gene dosage and metabolic requirements shaped the time-dependent resolution of yeast polyploidy. *Mol Biol Evol*. 2014; 31:3184–3193.

14. De Smet R, Adams KL, Vandepoele K, Van Montagu MCE, Maere S, Van de Peer Y. Convergent gene loss following gene and genome duplications creates single-copy families in flowering plants. *Proc Natl Acad Sci U S A*. 2013;110:2898–2903.

15. Freeling M, Woodhouse MR, Subramaniam S, Turco G, Lisch D, Schnable JC. Fractionation mutagenesis and similar consequences of mechanisms removing dispensable or less-expressed DNA in plants. *Curr Opin Plant Biol*. 2012;15:131–139.

16. Birchler JA, Veitia RA. The gene balance hypothesis: implications for gene regulation, quantitative traits and evolution. *New Phytol*. 2010;186:54–62.

17. Rody HVS, Baute GJ, Rieseberg LH, Oliveira LO. Both mechanism and age of duplications contribute to biased gene retention patterns in plants. *BMC Genomics*. 2017;18:46.

18. Tasdighian S, Van Bel M, Li Z, Van de Peer Y, Carretero-Paulet L, Maere S. Reciprocally retained genes in the angiosperm lineage show the hallmarks of dosage balance

sensitivity. *Plant Cell*. 2017; 29:2766–2785.

19. Cheng F, Wu J, Fang L, Sun S, Liu B, Lin K, et al.. Biased gene fractionation and dominant gene expression among the subgenomes of *Brassica rapa*. *PLoS One*. 2012;7:e36442.

20. Renny-Byfield S, Rodgers-Melnick E, Ross-Ibarra J. Gene fractionation and function in the ancient subgenomes of Maize. *Mol Biol Evol*. 2017;34:1825–1832.

21. Thomas BC, Pedersen B, Freeling M. Following tetraploidy in an *Arabidopsis* ancestor, genes were removed preferentially from one homeolog leaving clusters enriched in dose-sensitive genes. *Genome Res*. 2006;16:934–946.

22. Woodhouse MR, Schnable JC, Pedersen BS, Lyons E, Lisch D, Subramaniam S, et al.. Following tetraploidy in Maize, a short deletion mechanism removed genes preferentially from one of the two homeologs. *PLoS Biol*. Public Library of Science. 2010;8:e1000409.

23. Garsmeur O, Schnable JC, Almeida A, Jourda C, D’Hont A, Freeling M. Two evolutionarily distinct classes of paleopolyploidy. *Mol Biol Evol*. 2014;31:448–454.

24. Schnable JC, Springer NM, Freeling M. Differentiation of the maize subgenomes by genome dominance and both ancient and ongoing gene loss. *Proc Natl Acad Sci U S A*. 2011;108:4069–4074.

25. Edger PP, Smith R, McKain MR, Cooley AM, Vallejo-Marin M, Yuan Y, et al.. Subgenome dominance in an interspecific hybrid, synthetic allopolyploid, and a 140-year-old naturally established neo-allopolyploid monkeyflower. *Plant Cell*. 2017;29:2150–2167.

26. Linder HP, Lehmann CER, Archibald S, Osborne CP, Richardson DM. Global grass (Poaceae) success underpinned by traits facilitating colonization, persistence and habitat

transformation. *Biological Reviews of the Cambridge Philosophical Society*. 2018;*93*(2); 1125–1144.

27. Stebbins GL, Ledyard Stebbins G. Polyploidy, hybridization, and the invasion of new habitats. Annals of the Missouri Botanical Garden. 1985;*72*(4):824–832.

28. Estep MC, McKain MR, Vela Diaz D, Zhong J, Hodge JG, Hodkinson TR, et al.. Allopolyploidy, diversification, and the Miocene grassland expansion. *Proc Natl Acad Sci U S A*. 2014111:15149–15154.

29. Watson L, Macfarlane TD, Dallwitz MJ. The grass genera of the world: descriptions, illustrations, identification, and information retrieval; including synonyms, morphology, anatomy, physiology, phytochemistry, cytology, classification, pathogens, world and local distribution and references. 1992.

30. Harlan JR, de Wet JMJ. The compilospecies concept. *Evolution; International Journal of Organic Evolution*. 1963;17(4):497–501.

31. Wet JMJ, Harlan JR. *Bothriochloa intermedia* — a taxonomic dilemma. *Taxon*. 1970; 19:339–340.

32. Sumadijaya A. Morphology, molecular phylogeny and genome content of *Bothriochloa* focusing on Australian taxa. Virginia Tech. 2015.

33. Putnam NH, O'Connell BL, Stites JC, Rice BJ, Blanchette M, Calef R, et al.. Chromosome-scale shotgun assembly using an in vitro method for long-range linkage. *Genome Res*. 2016; 26:342–350.

34. De Wet JMJ, Harlan JR. Morphology of the compilospecies *Bothriochloa intermedia*. *Am J Bot*. 1966;53:94–98.

35. De Wet JMJ, Higgins ML. Species relationships within the *Bothriochloa pertusa* complex. *Phyton, Vicente Lopez*. 1963.

36. Grabherr MG, Haas BJ, Yassour M, Levin JZ, Thompson DA, Amit I, et al.. Full-length transcriptome assembly from RNA-Seq data without a reference genome. *Nature Biotechnology*. 2011; 29:644–652.

37. Cantarel BL, Korf I, Robb SMC, Parra G, Ross E, Moore B, et al.. MAKER: an easy-to-use annotation pipeline designed for emerging model organism genomes. *Genome Res*. 2008;18:188–196

38. Stanke M, Schöffmann O, Morgenstern B, Waack S. Gene prediction in eukaryotes with a generalized hidden Markov model that uses hints from external sources. *BMC Bioinformatics*. 2006;7:62.

39. Korf I. Gene finding in novel genomes. *BMC Bioinformatics*. 2004;5:59.

40. Campbell MS, Law M, Holt C, Stein JC, Moghe GD, Hufnagel DE, et al.. MAKER-P: a tool kit for the rapid creation, management, and quality control of plant genome annotations. *Plant Physiol*. 2014;164:513–524.

41. Camacho C, Coulouris G, Avagyan V, Ma N, Papadopoulos J, Bealer K, et al.. BLAST+: architecture and applications. *BMC Bioinformatics*. 2009;10:421.

42. Wang Y, Tang H, Debarry JD, Tan X, Li J, Wang X, et al.. MCScanX: a toolkit for detection and evolutionary analysis of gene synteny and collinearity. *Nucleic Acids Res*. 2012;40:e49.

43. Li H. Minimap2: pairwise alignment for nucleotide sequences. *Bioinformatics*. 2018;34:3094–3100.

44. Session AM, Uno Y, Kwon T, Chapman JA, Toyoda A, Takahashi S, et al.. Genome evolution in the allotetraploid frog *Xenopus laevis*. *Nature*. 2016;538:336–343.

45. Mitros T, Session AM, James BT, Wu GA, Belaffif MB, Clark LV, et al.. Genome biology of the paleotetraploid perennial biomass crop *Miscanthus*. *Nat Commun*. 2020;11:5442.

46. Himmelmann ML: Package "HMM." https://cran.opencpu.org/web/packages/HMM/HMM.pdf (2022). Accessed 2022 Mar 29.

47. Emms DM, Kelly S. OrthoFinder: phylogenetic orthology inference for comparative genomics. *Genome Biol*. 2019; 20:238.

48. Alexa A, Rahnenfuhrer J. TopGO: enrichment analysis for gene ontology. R package version. 2006.

49. Sankoff D, Zheng C, Zhu Q. The collapse of gene complement following whole genome duplication. *BMC Genomics*. 2010;11:313.

50. Schnable PS, Ware D, Fulton RS, Stein JC, Wei F, Pasternak S, et al.. The B73 maize genome: complexity, diversity, and dynamics. *Science*. 2009;*326*(5956):1112–1115.

51. Griffiths AG, Moraga R, Tausen M, Gupta V, Bilton TP, Campbell MA, et al.. Breaking free: The genomics of allopolyploidy-facilitated niche expansion in white clover. *Plant Cell*. 2019;31:1466–1487.

52. Hirsch CD, Springer NM. Transposable element influences on gene expression in plants. *Biochim Biophys Acta Gene Regul Mech*. 2017;1860:157–165.

53. Stroud H, Greenberg MVC, Feng S, Bernatavichute YV, Jacobsen SE. Comprehensive

analysis of silencing mutants reveals complex regulation of the *Arabidopsis* methylome. *Cell*. 2013;152:352–364.

54. Weber M, Hellmann I, Stadler MB, Ramos L, Pääbo S, Rebhan M, et al.. Distribution, silencing potential and evolutionary impact of promoter DNA methylation in the human genome. *Nat Genet*. 2007; 39:457–466.

55. Martienssen RA, Colot V. DNA methylation and epigenetic inheritance in plants and filamentous fungi. *Science*. 2001; 293:1070–1074.

56. Blanc G, Wolfe KH. Functional divergence of duplicated genes formed by polyploidy during *Arabidopsis* evolution. *Plant Cell*. 2004;16:1679–1691.

57. Duarte JM, Wall PK, Edger PP, Landherr LL, Ma H, Pires JC, et al.. Identification of shared single copy nuclear genes in *Arabidopsis*, *Populus*, *Vitis* and *Oryza* and their phylogenetic utility across various taxonomic levels. *BMC Evol Biol*. 2010;10:61.

58. Maere S, De Bodt S, Raes J, Casneuf T, Van Montagu M, Kuiper M, et al.. Modeling gene and genome duplications in eukaryotes. *Proc Natl Acad Sci U S A*. 2005; 102:5454–5459.

59. Paterson AH, Chapman BA, Kissinger JC, Bowers JE, Feltus FA, Estill JC. Many gene and domain families have convergent fates following independent whole-genome duplication events in *Arabidopsis*, *Oryza*, *Saccharomyces* and *Tetraodon*. *Trends Genet*. 2006;22:597–602.

60. Seoighe C, Gehring C. Genome duplication led to highly selective expansion of the *Arabidopsis thaliana* proteome. *Trends Genet*. 2004;20:461–464.

61. Haig D. From Darwin to Derrida: Selfish genes, social selves, and the meanings of life.

MIT Press.2020.

62. Kleine T, Voigt C, Leister D. Plastid signalling to the nucleus: messengers still lost in the mists? *Trends Genet*. 2009; 25:185–192.

63. Emery M, Willis MMS, Hao Y, Barry K, Oakgrove K, Peng Y, et al.. Preferential retention of genes from one parental genome after polyploidy illustrates the nature and scope of the genomic conflicts induced by hybridization. *PLoS Genet*. 2018;14:e1007267

64. Wu S, Han B, Jiao Y. Genetic contribution of paleopolyploidy to adaptive evolution in angiosperms. *Mol Plant*. 2020;13:59–71.

65. Cheng F, Wu J, Cai X, Liang J, Freeling M, Wang X. Gene retention, fractionation and subgenome differences in polyploid plants. *Nat Plants*. 2019;4:258–268.

66. Defoort J, Van de Peer Y, Carretero-Paulet L. The Evolution of gene duplicates in angiosperms and the impact of protein–protein interactions and the mechanism of duplication. *Genome Biol Evol*. Oxford Academic. 2019;11:2292–2305.

67. Jiao H, Wang Y, Zhang L, Jiang P, Zhao H. Lineage-specific duplication and adaptive evolution of bitter taste receptor genes in bats. *Mol Ecol*. 2018; 27:4475–4488.

68. Panchy N, Lehti-Shiu M, Shiu S-H. Evolution of gene duplication in plants. *Plant Physiol*. 2016;171:2294–2316.

69. Zhang L, Wu S, Chang X, Wang X, Zhao Y, Xia Y, et al.. The ancient wave of polyploidization events in flowering plants and their facilitated adaptation to environmental stress. *Plant Cell Environ*. 2020; 43:2847–2856.

70. Edwards EJ, Osborne CP, Strömberg CAE, Smith SA, C4 Grasses Consortium, Bond WJ,

et al.. The origins of C4 grasslands: integrating evolutionary and ecosystem science. *Science*. 2010;328:587–591.

71. Wicker T, Keller B. Genome-wide comparative analysis of copia retrotransposons in Triticeae, rice, and *Arabidopsis* reveals conserved ancient evolutionary lineages and distinct dynamics of individual copia families. *Genome Res*. 2007;17:1072–1081.

72. Godfree RC, Marshall DJ, Young AG, Miller CH, Mathews S. Empirical evidence of fixed and homeostatic patterns of polyploid advantage in a keystone grass exposed to drought and heat stress. *R Soc Open Sci*. 2017; 4:170934.

73. Linder HP, Barker NP. Does polyploidy facilitate long-distance dispersal? *Ann Bot*. 2014;113:1175–1183.

74. Breed MF, Stead MG, Ottewell KM, Gardner MG, Lowe AJ. Which provenance and where? Seed sourcing strategies for revegetation in a changing environment. *Conserv Genet*.2013; 14:1–10.

75. Aitken SN, Whitlock MC. Assisted gene flow to facilitate local adaptation to climate change. *Annu Rev Ecol Evol Syst*. Annual Reviews.2013; 44:367–388.

76. Stanley TD, Ross EM. Flora of south-eastern Queensland. Queensland Dept. of Primary Industries.1983.

77. Simon BK, Alfonso Y. AusGrass2. Brisbane, Queensland, Australia.2011.

78. Dolezel J, Greilhuber J, Suda J. Estimation of nuclear DNA content in plants using flow cytometry. *Nat Protoc*. 2007;2:2233–2244.

79. Galbraith DW, Lambert GM. High-throughput monitoring of plant nuclear DNA contents

via flow cytometry. *Methods Mol Biol*. 2012; 918:311–325.

80. Lieberman-Aiden E, van Berkum NL, Williams L, Imakaev M, Ragoczy T, Telling A, et al.. Comprehensive mapping of long-range interactions reveals folding principles of the human genome. *Science*. 2009; 326:289–293.

81. Weisenfeld NI, Kumar V, Shah P, Church DM, Jaffe DB. Direct determination of diploid genome sequences. *Genome Res*. 2017;27:757–767.

82. Zaharia M, Bolosky WJ, Curtis K, Fox A, Patterson D, Shenker S, et al.. Faster and more accurate sequence alignment with SNAP. 2011. arXiv. http://arxiv.org/abs/1111.5572.

83. Robinson JT, Turner D, Durand NC, Thorvaldsdóttir H, Mesirov JP, Aiden EL. Juicebox.js provides a cloud-based visualization system for Hi-C Data. *Cell Syst*. 2018;*6*(2):256-258.

84. Bolger AM, Lohse M, Usadel B. Trimmomatic: a flexible trimmer for Illumina sequence data. *Bioinformatics*. 2014;30:2114–2120.

85. Han Y, Wessler SR. MITE-Hunter: a program for discovering miniature inverted-repeat transposable elements from genomic sequences. *Nucleic Acids Res*. 2010;38:e199.

86. Ellinghaus D, Kurtz S, Willhoeft U. LTRharvest, an efficient and flexible software for de novo detection of LTR retrotransposons. *BMC Bioinformatics*. 2008; 9:18.

87. Steinbiss S, Willhoeft U, Gremme G, Kurtz S. Fine-grained annotation and classification of de novo predicted LTR retrotransposons. *Nucleic Acids Res*. 2009;37:7002–7013.

88. Smit AFA, Hubley R. RepeatModeler (v.2.0.3). 2015. http://www.repeatmasker.org. Accessed 10 November 2020.

89. Plant Protein Database. http://www.hrt.msu.edu/uploads/535/78637/alluniRefprexp070416.gz. Accessed 20 January 2020.

90. All Transposase Protein Database. http://www.hrt.msu.edu/uploads/535/78637/Tpases020812.gz. Accessed 2 February 2020.

91. Smit AFA, Hubley R, Green P. *RepeatMasker Open-4.0*. http://repeatmasker.org. 2015.

92. Kennedy RC, Unger MF, Christley S, Collins FH, Madey GR. An automated homology-based approach for identifying transposable elements. *BMC Bioinformatics*. 2011;12:130.

93. Jiang N, Bao Z, Zhang X, Hirochika H, Eddy SR, McCouch SR, et al.. An active DNA transposon family in rice. *Nature*. 2003;421:163–167.

94. Altschul SF, Gish W, Miller W, Myers EW, Lipman DJ. Basic local alignment search tool. *J Mol Biol*. 1990; 215:403–410.

95. Storer J, Hubley R, Rosen J, Wheeler TJ, Smit AF. The Dfam community resource of transposable element families, sequence models, and genome annotations. *Mob DNA*.2021; 12:2.

96. Eddy SR. A new generation of homology search tools based on probabilistic inference. *Genome Inform*. 2009; 23:205–211.

97. Dfamscan.pl. https://dfam.org/releases/current/infrastructure/dfamscan.pl.gz. Accessed 11 March 2020.

98. Seppey M, Manni M, Zdobnov EM. BUSCO: Assessing genome assembly and annotation completeness. *Methods Mol Biol*. 2019;1962:227–245.

99. Genestats. https://github.com/darencard/GenomeAnnotation/blob/97fa52d13eb7a8f6b59a0f6e7261e1e08e542126/genestats. Accessed 14 May 2021.

100. Manni M, Berkeley MR, Seppey M, Simão FA, Zdobnov EM. BUSCO update: novel and streamlined workflows along with broader and deeper phylogenetic coverage for scoring of eukaryotic, prokaryotic, and viral genomes. *Mol Biol Evol*. 2021; 38:4647–4654.

101. Winter D, Lee K, Cox M. Pafr: Read, manipulate and visualize “pairwise mapping format” Data. R package version 0.0.2. 2020.

102. Marçais G, Kingsford C. A fast, lock-free approach for efficient parallel counting of occurrences of k-mers. *Bioinformatics*. 2011;27:764–770.

103. Gu Z, Eils R, Schlesner M. Complex heatmaps reveal patterns and correlations in multidimensional genomic data. *Bioinformatics*. 2016;32:2847–2849.

104. van Dongen S, Abreu-Goodger C. Using MCL to extract clusters from networks. *Methods Mol Biol*. 2012;804:281–295.

105. UniProt Consortium. UniProt: the universal protein knowledgebase in 2021. *Nucleic Acids Res*. 2021; 49(D1):D480–D489.

106. Berardini TZ, Reiser L, Li D, Mezheritsky Y, Muller R, Strait E, et al.. The *Arabidopsis* information resource: Making and mining the “gold standard” annotated reference plant genome. *Genesis*. 2015;53:474–485.

107. Quevillon E, Silventoinen V, Pillai S, Harte N, Mulder N, Apweiler R, et al.. InterProScan: protein domains identifier. *Nucleic Acids Res*. 2005; *33*(Web Server issue):W116–W120.

108. Punta M, Coggill PC, Eberhardt RY, Mistry J, Tate J, Boursnell C, et al.. The Pfam protein families database. *Nucleic Acids Res*. 2012; 40:D290–D301.

109. Kanehisa M, Goto S. KEGG: kyoto encyclopedia of genes and genomes. *Nucleic Acids Res*. 2000;28:27–30.

110. Kanehisa M. Toward understanding the origin and evolution of cellular organisms. *Protein Sci*. 2019; 28:1947–1951.

111. Kanehisa M, Furumichi M, Sato Y, Ishiguro-Watanabe M, Tanabe M. KEGG: integrating viruses and cellular organisms. *Nucleic Acids Res*. 2021; 49:D545–D551.

112. Aramaki T, Blanc-Mathieu R, Endo H, Ohkubo K, Kanehisa M, Goto S, et al.. KofamKOALA: KEGG Ortholog assignment based on profile HMM and adaptive score threshold. *Bioinformatics*. 2020;36:2251–2252.

113. Bandi V, Gutwin C. Interactive exploration of genomic conservation. 2020. https://openreview.net/pdf?id=7-C5VJWbnI.

114. Goodstein DM, Shu S, Howson R, Neupane R, Hayes RD, Fazo J, et al.. Phytozome: a comparative platform for green plant genomics. *Nucleic Acids Res*. 2012;40:D1178–1186.

115. Zhang J, Zhang X, Tang H, Zhang Q, Hua X, Ma X, et al.. Allele-defined genome of the autopolyploid sugarcane *Saccharum spontaneum* L. *Nat Genet*. 2018;50:1565–1573.

116. Mao Y. GenoDup Pipeline: a tool to detect genome duplication using the dS-based method. *PeerJ*. 2019;7:e6303.

117. Katoh K, Standley DM. MAFFT multiple sequence alignment software version 7: improvements in performance and usability. *Mol Biol Evol*. 2013;30:772–780.

118. Talavera G, Castresana J. Improvement of phylogenies after removing divergent and ambiguously aligned blocks from protein sequence alignments. *Syst Biol*. 2007;56:564–577.

119. Darriba D, Taboada GL, Doallo R, Posada D. jModelTest 2: more models, new heuristics and parallel computing. *Nat Methods*. 2012; 9:772.

120. Guindon S, Gascuel O. A simple, fast, and accurate algorithm to estimate large phylogenies by maximum likelihood. *Syst Biol*. 2003;52:696–704.

121. Stamatakis A. RAxML version 8: a tool for phylogenetic analysis and post-analysis of large phylogenies. *Bioinformatics*. 2014; 30:1312–1313.

122. Bouckaert R, Vaughan TG, Barido-Sottani J, Duchêne S, Fourment M, Gavryushkina A, et al.. BEAST 2.5: An advanced software platform for Bayesian evolutionary analysis. *PLoS Comput Biol*. 2019;15:e1006650.

123. Suchard MA, Lemey P, Baele G, Ayres DL, Drummond AJ, Rambaut A. Bayesian phylogenetic and phylodynamic data integration using BEAST 1.10. *Virus Evol*. 2018; 4:vey016.

124. Bouckaert RR, Drummond AJ. bModelTest: Bayesian phylogenetic site model averaging and model comparison. *BMC Evol Biol*. 2017;17:42.

125. Kumar S, Stecher G, Suleski M, Hedges SB. TimeTree: A resource for timelines, timetrees, and divergence times. *Mol Biol Evol*. 2017; 34:1812–1819.

126. Rambaut A, Drummond AJ, Xie D, Baele G, Suchard MA. Posterior summarization in Bayesian phylogenetics using Tracer 1.7. *Syst Biol*. 2018; 67:901–904.

127. Paradis E, Schliep K. Ape 5.0: An environment for modern phylogenetics and

evolutionary analyses in R. *Bioinformatics*. Oxford University Press (OUP). 2019;35:526–528.

128. Moniz de Sá M, Drouin G. Phylogeny and substitution rates of angiosperm actin genes. *Mol Biol Evol*. 1996;13:1198–1212.

129. Ma J, Bennetzen JL. Rapid recent growth and divergence of rice nuclear genomes. *Proc Natl Acad Sci U S A*. 2004;101:12404–12410.

130. https://github.com/NissankaPD/B_decipiens_genome.

Figure_1 Click here to access/download;Figure;Figure_1.png

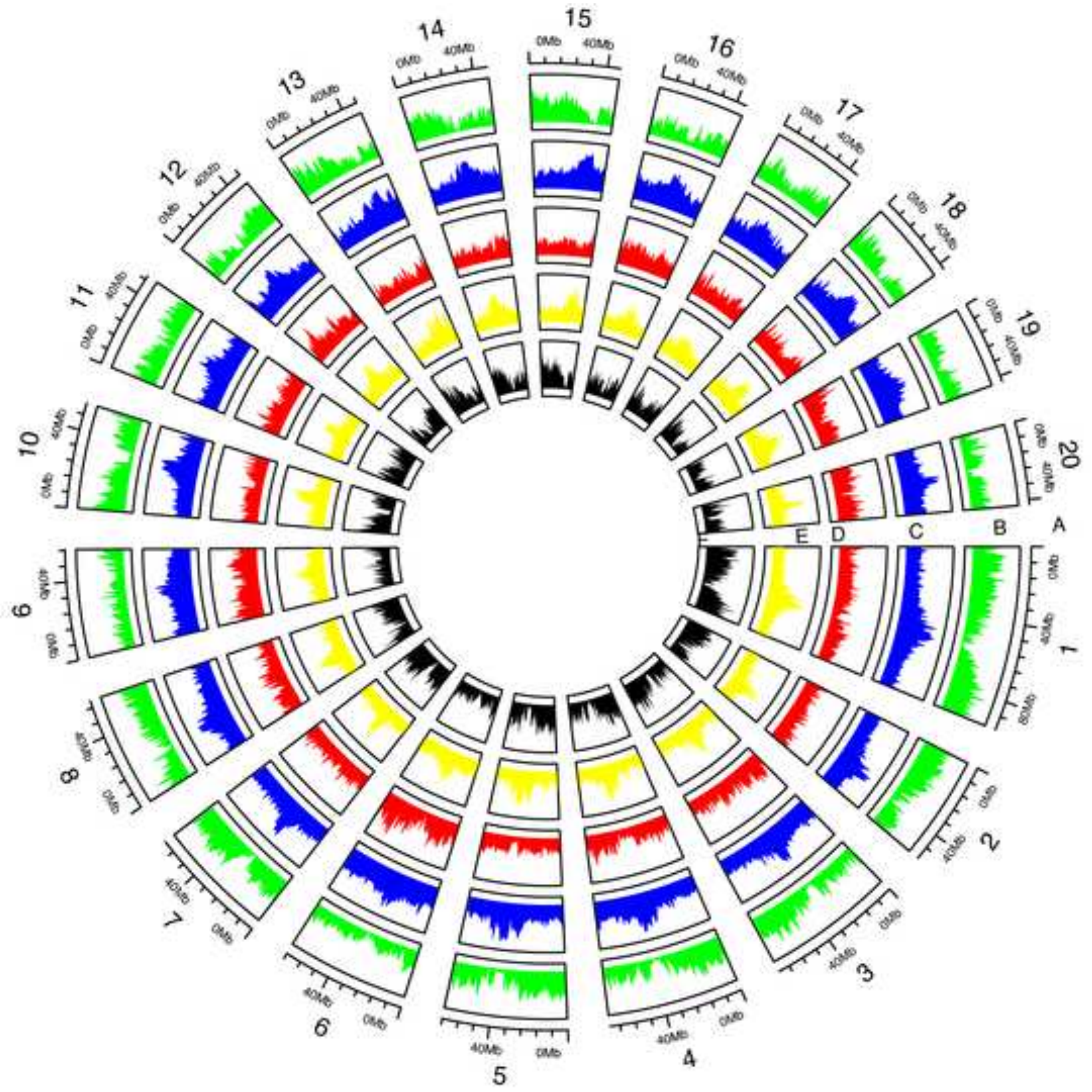

Figure_2

Click here to access/download;Figure;Figure_2.png

A

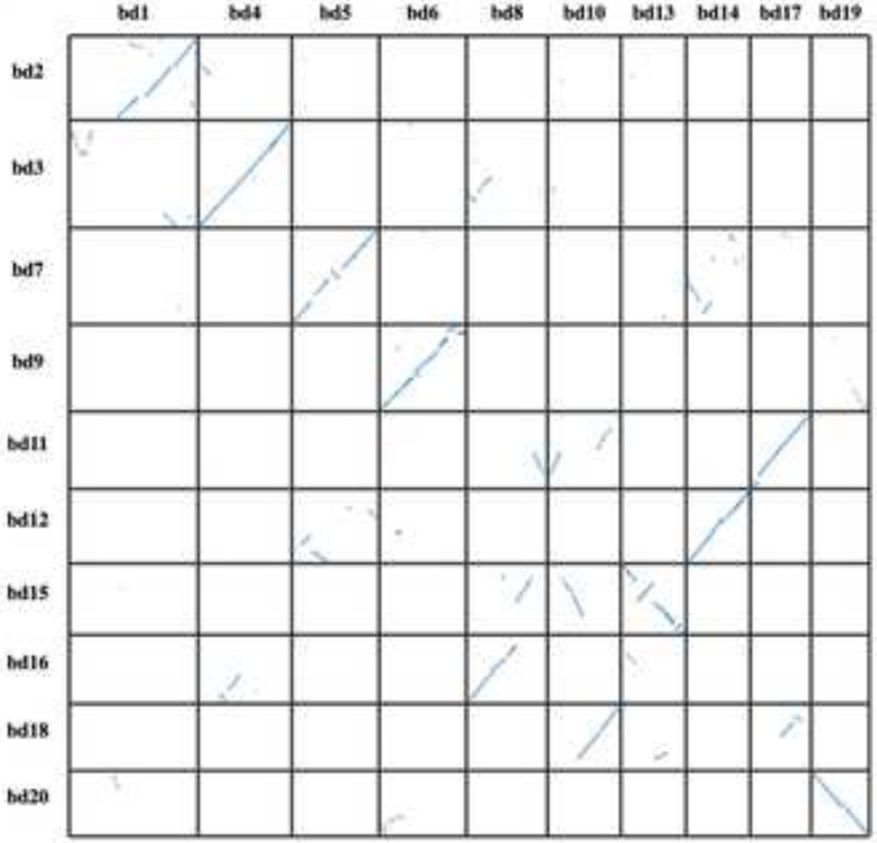

B

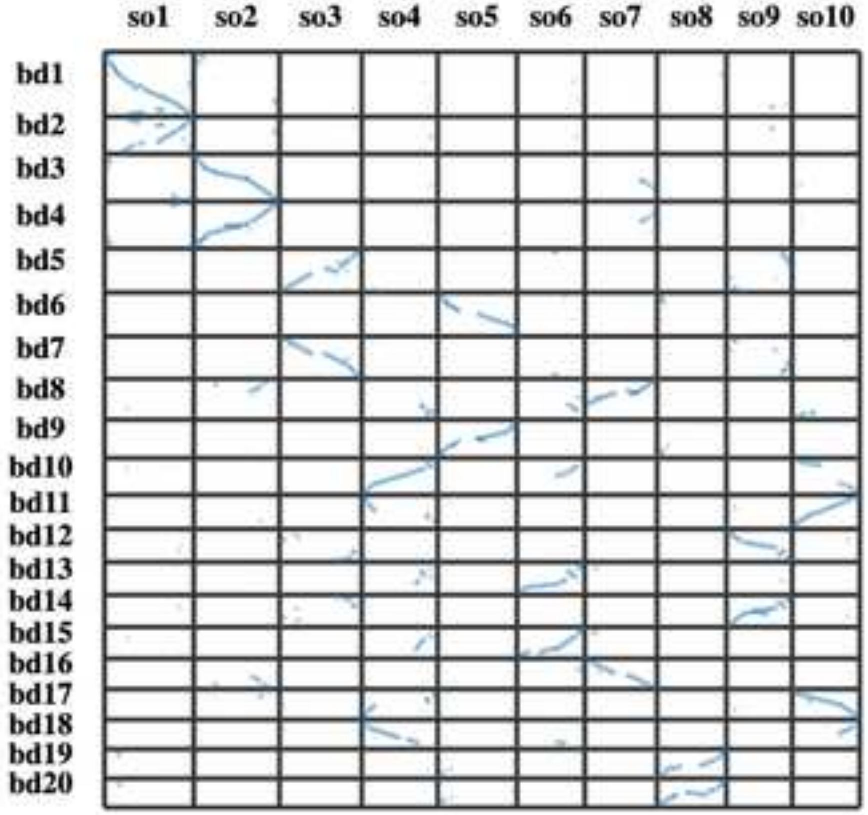

Figure_3

Click here to access/download;Figure;Figure_3.png

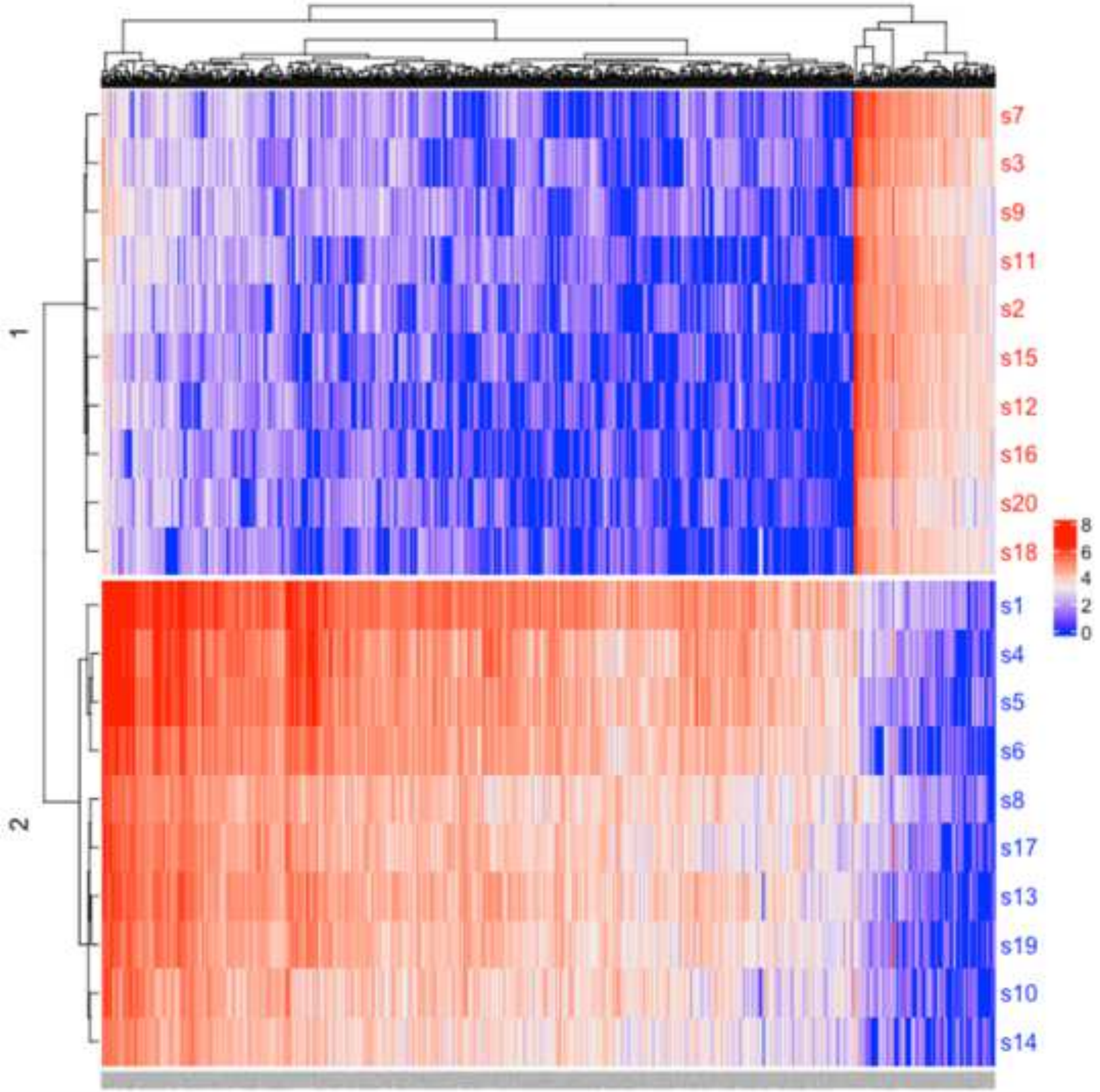

Figure_4

Click here to access/download;Figure;Figure_4.png

A

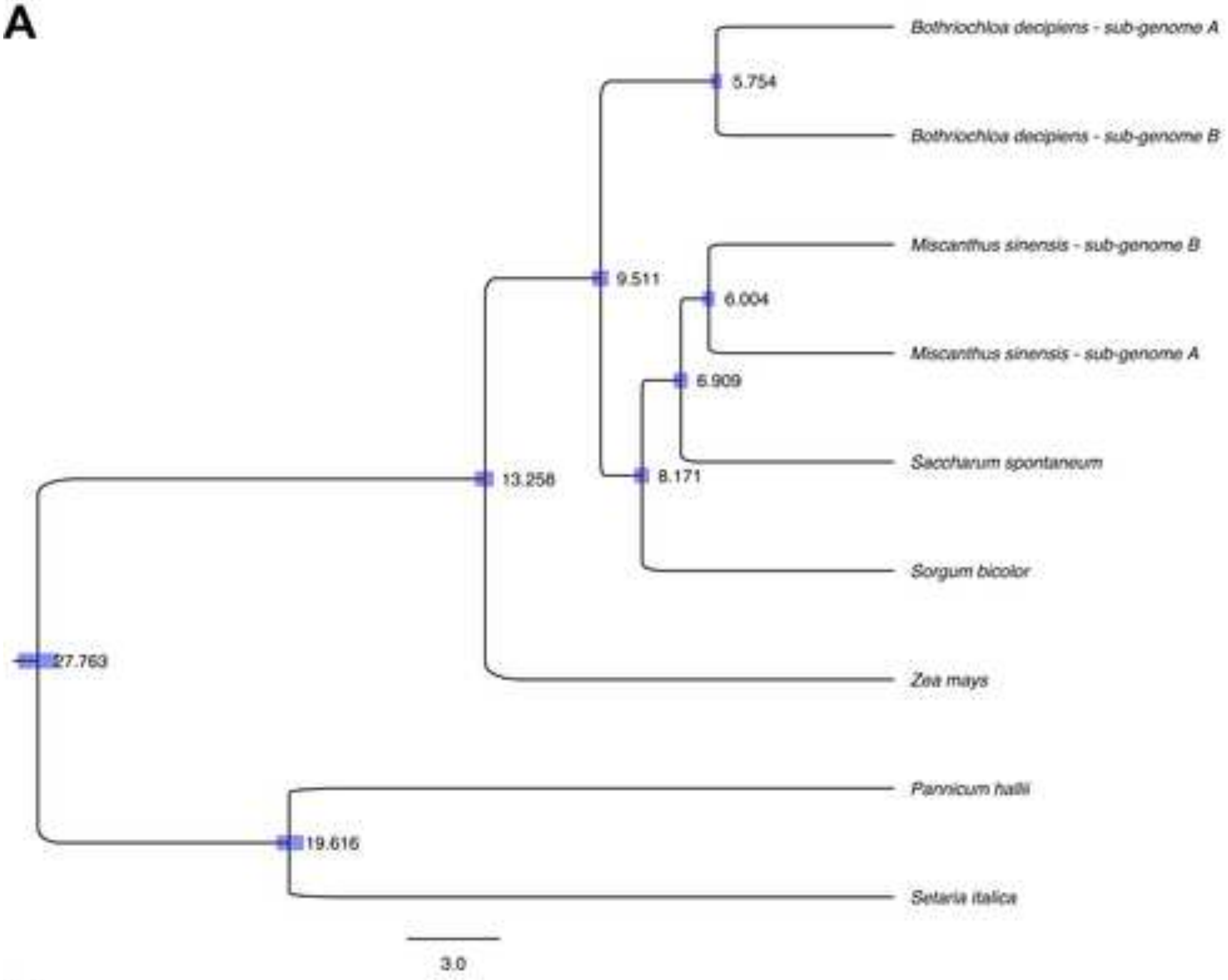

B

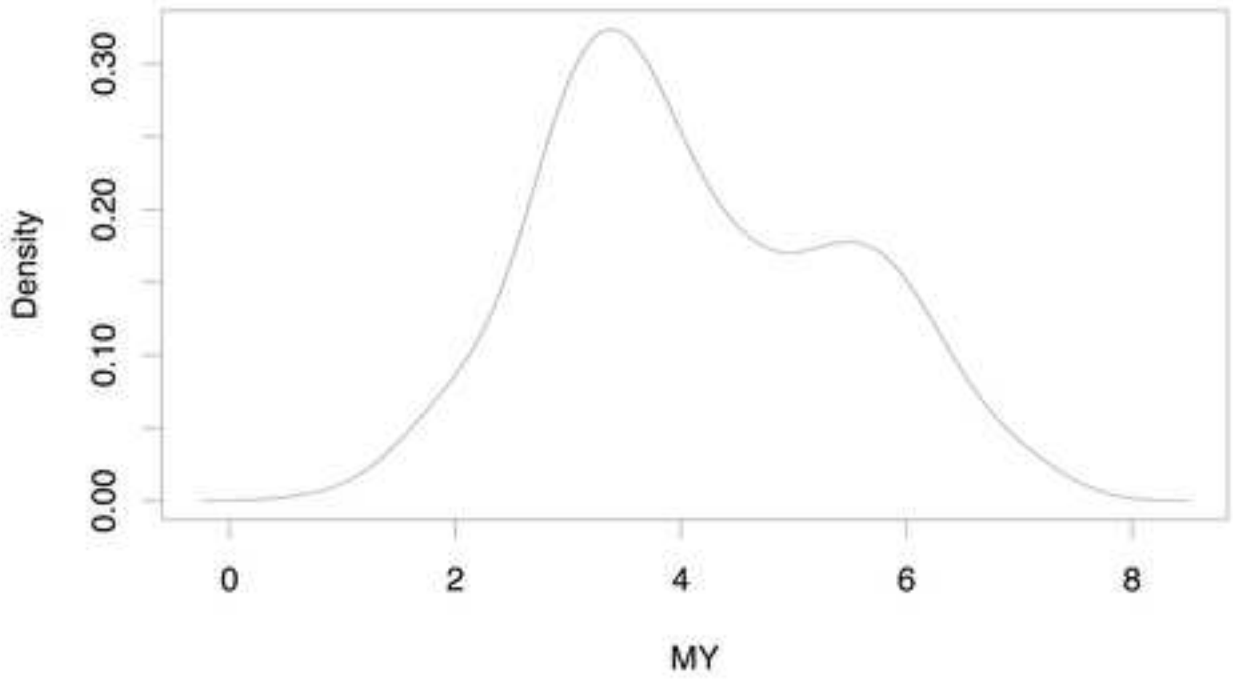

Figure_5

Click here to access/download;Figure;Figure_5.png

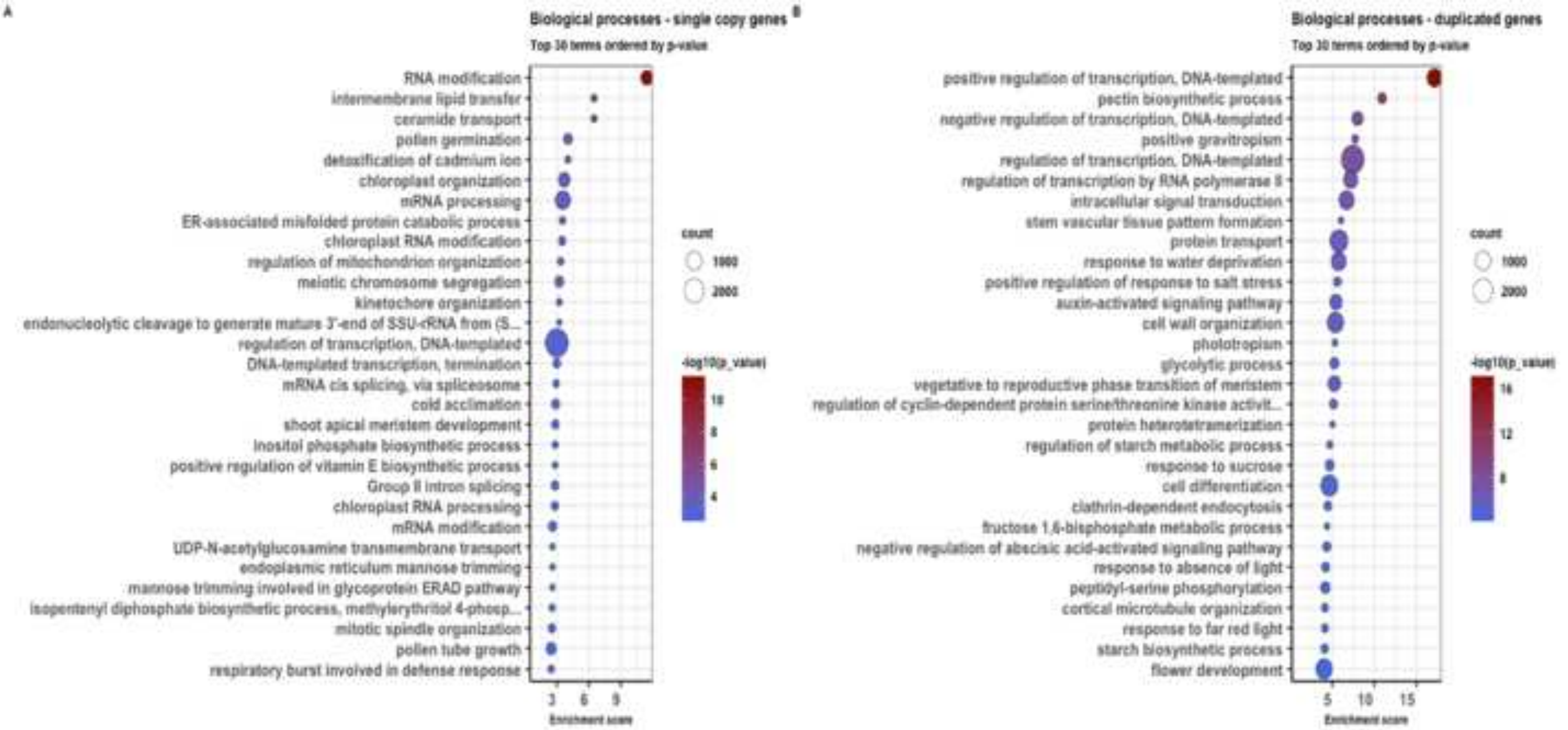

Supplementary Material- Figure_S1

Click here to access/download
Supplementary Material
Supplementary_Figure_S1.png

Supplementary Material-Figure_S2

Click here to access/download
Supplementary Material
Supplementary_figure_S2.png

Click here to access/download
Supplementary Material
Supplementary_Figure_S3.png

Click here to access/download
Supplementary Material
Supplementary_Figure_S4.png

Supplementary Material-Figure_S5

Click here to access/download
Supplementary Material
Supplementary_Figure_S5.png

Click here to access/download
Supplementary Material
Supplementary_Figure_S6.png

Click here to access/download
Supplementary Material
Supplementary_Figure_S7.png

Click here to access/download
Supplementary Material
Supplementary_Figure_captions_and_tables.docx
