# Supplementary material for: Genome assembly of an Australian native grass species reveals a recent whole-genome duplication and biased gene retention of genes involved in stress response
Source: Gigascience. 2023 May 12;12:giad034. doi: 10.1093/gigascience/giad034 (PMC10176504; doi:10.1093/gigascience/giad034)
Supplement: giad034_GIGA-D-22-00164_Revision_1 [file giad034_giga-d-22-00164_revision_1.pdf]

GigaScience

Genome assembly of an Australian native grass species reveals a recent whole genome duplication and biased gene retention of genes involved in stress response

--Manuscript Draft--

Manuscript Number:	GIGA-D-22-00164R1
Full Title:	Genome assembly of an Australian native grass species reveals a recent whole genome duplication and biased gene retention of genes involved in stress response
Article Type:	Research
Funding Information:	Hermon Slade Foundation (HSF1703) — Dr Kathryn Hodgins
Abstract:	Background<br><br>The adaptive significance of polyploidy has been extensively debated and chromosome level genome assemblies of polyploids can provide insight into this topic. The Australian grass, Bothriochloa decipiens, belongs to the BCD clade, a group with a complex history of hybridization and polyploidy. This is the first genome assembly and annotation of a species that belongs to this fascinating yet complex group.<br><br>Findings<br><br>Using a combination of Illumina short reads, 10X Genomics linked reads and Hi-C sequencing data we assembled a highly contiguous genome of Bothriochloa decipiens , with a total length of 1,218.22 Mb and scaffold N50 of 42.637 Mb. Comparative analysis revealed that the species is a diploidized allotetraploid. We clustered the 20 major scaffolds, representing the 20 chromosomes, into the two sub genomes of the parental species using unique repeat signatures. Found evidence of biased fractionation and differences in the activity of transposable elements between the sub genomes prior to hybridization. Duplicates were enriched for genes involved in transcription and response to external stimuli like drought, supporting a biased retention of duplicated genes following whole genome duplication.<br><br>Conclusions<br><br>Our results support hypotheses explaining the biased retention of duplicated genes following polyploidy and point to differences in repeat activity associated with sub genome dominance. Bothriochloa decipiens is a widespread species with the ability to establish across many soil types, making it useful for ecological restoration of Australian grasslands. This reference genome is a valuable resource for future population genomic research involving Australian grasses which may be helpful in ecological restoration projects.
Corresponding Author:	Nissanka Prasangi De Silva, Ph.D<br>Monash University School of Biological Sciences<br>Clayton, Victoria AUSTRALIA
Corresponding Author Secondary Information:	
Corresponding Author's Institution:	Monash University School of Biological Sciences
Corresponding Author's Secondary Institution:	
First Author:	Nissanka Prasangi De Silva, B.Sc
First Author Secondary Information:	
Order of Authors:	Nissanka Prasangi De Silva, B.Sc<br>Christopher Lee, Ph.D<br>Paul Battlay, Ph.D

Powered by Editorial Manager® and ProduXion Manager® from Aries Systems Corporation

	Alexandre Fournier-Level, Ph.D
	Joslin Moore, Ph. D
	Kathryn Hodgins, Ph.D
**Order of Authors Secondary Information:**	
**Response to Reviewers:**	REVIEWER COMMENTS

Reviewer #1: (Remarks to the Author):

This manuscript describes the genome sequencing project of Bothriochloa decipiens, an Australian grass from BCD clade. Using the newly sequenced genome of B. decipiens, the authors have found evidence of biased fractionation and differences in LTR-retrotransposons activity between the sub genomes prior to hybridization. The method used to organise the sub-genomes using repeat signatures is interesting. Also, future work with more in-depth analysis revealing the relationship between the active LTRs and high number of retained genes in sub-genome A would be really informative. Overall, as BCD clade represents species with a complex history of hybridization and polyploidy, the availability of chromosomal level genome of Bothriochloa decipiens would facilitate understanding evolutionary studies at various levels. Hence, I accept the manuscript for publication.

The placement of figure legends in between the main document texts (Pg 8:153-156, 10:187-191, 11:232-234, 12:259-262, 15:295-296) is hard to follow. Please keep all figure legends in one page.
We have now removed the figure legends in between the main text document and added all figure legends to page 40 (line 794-810).

Reviewer #2: (Remarks to the Author):

De Silva et al. present a chromosome-scaffolded genome of Bothriochloa decipiens. The manuscript is primarily a genome report, focusing on gene content and pattern of gene retention post whole genome duplication. There are a few areas that the authors should consider in a revision to improve the genome annotation and subsequent analyses.

The transposon annotation and description could use more detail on the superfamilies that were identified. More diversity of superfamilies, especially those considered DNA elements, should be described. The methods for annotating the elements focused on LTRs and MITEs. There is no indication of annotation for elements like helitrons. I would recommend using software like EDTA since it covers the majority of TE types.
We thank the reviewer for this suggestion to expand our repeat annotation to include more details on the repeat family diversity present in the genome. We have now used the EDTA pipeline as suggested by the reviewer to further annotate the repeats and have included a table depicting the major repeat classes and the superfamilies present within these major classes along with the percentage of the genome covered by each superfamily (Table 3, page 9). Further we have included a new section in our methods “Detailed identification of repeat family diversity in the genome” to describe the use of the EDTA pipeline for the repeat annotations page 31 (lines 592-598).

The finding that the more dominant subgenome also had more TE activity is interesting. It would be useful for the authors to describe the location of TEs relative to gene space. Other studies have demonstrated that there is a link between TE density near genes and paralog loss post WGD.
We thank the reviewer for this interesting suggestion. In order to address this suggestion we used a pipeline name “TE density” (Teresi et al., 2022) that calculates TE density defined as the number of TE-occupied base pairs in a given window divided by the window value relative to the genes in the genome. We calculated the TE densities using this for all the genes present in the genome as well as genes identified as single copy and duplicate copy as identified previously by us in our analyses to identify biases in gene retention between the subgenomes. Using these calculated average TE densities with relation to gene space, we used Mann-Whitney U tests to

Powered by Editorial Manager® and ProduXion Manager® from Aries Systems Corporation

assess if the average TE density values were significantly different between the two subgenomes and also between genes identified as single copy or duplicated. We did not find any evidence of significant difference in TE density values in relation to gene space between the two subgenomes or between the genes retained as single copy or as duplicates. This new analysis is reported in the manuscript in page 17 lines 263-283. The methods followed for this new addition is written in page 37 (lines 727-741).

Check the spelling of "Andropogoneae" and "homoeologous" throughout.
We thank the reviewer for pointing out this oversight. We have now checked each time the word “Andropogoneae” appears in the manuscript and corrected any spelling errors We have now checked each time the word “homoeologous” appears in the manuscript and corrected any spelling errors.

Figure 1: I would create a key that uses the track colours to assign the letters. As they are placed, they are difficult to see. Another option would be to refer to the track colours in the text instead of letters.
We thank the reviewer for pointing out this oversight. Now we have removed the letters from Figure 1 and are referring to the track colours in the figure legend instead of the letters page 40 (lines 795-798)

Line 70: Should not use the word "theory" this way. These are hypotheses.
We thank the reviewer for pointing out this oversight. We have now replaced the word “theories” with “hypotheses” in line 55.

Line 79: Should be written at "subgenome". This applies throughout the manuscript.
We thank the reviewer for pointing out this oversight. We have now checked all appearances of the word “subgenome” in the manuscript and amended where necessary.

Lines 81-84: I think this needs to be reworded. I believe the intention is to say that genes that have a higher expression level are more likely to be retained if one of the paralogs is lost. If a copy is pseudogenized and a comparison of expression of the two copies is made, it must be the one that is not pseudogenized that has a higher expression level.
We thank the reviewer for this suggestion to improve the clarity of the manuscript. We have now re-written the sentencein lines 62-68.

Line 120: It would be helpful to also cite some literature demonstrating B. decipiens has n = 20 chromosomes (at least in some accessions). Assemblies are not necessarily indicative of chromosome number—especially when some Hi-C methods require a target chromosome number in the scaffolding process. Chheda, de Wet, and Harlan (1961) is one such publication.
https://www.tandfonline.com/doi/pdf/10.1080/00087114.1961.10796026
We thank the reviewer for this suggestion. We have now included two references depicting the haploid chromosome number of B. decipiens including the one suggested by the reviewer and reworded the sentence to improve clarity in lines 95-96.

Table 1: "Total number" needs to indicate what this is a total number. I assume scaffolds/contigs, but this should be stated.
We have added the word “scaffolds” after “Total number” in Table 1.

Line 127: RNA-Seq or transcriptome sequencing is the common way to write this. RNA-Seq (or RNASeq) is a well-known method, so I would think you can get by without the need to describe what RNA stands for in parentheses.
We have removed the description in parentheses and replaced the word as RNA-Seq data in line 103.

Line 134: Missing something after [37] to connect the portions of the sentence.
We have re-written that sentence more clearly in line 111.

Powered by Editorial Manager® and ProduXion Manager® from Aries Systems Corporation

Lines 143-150: There should be more information regarding the annotation of TEs in the genome. The use of a program like EDTA (https://genomebiology.biomedcentral.com/articles/10.1186/s13059-019-1905-y) is recommended. A table depicting the different superfamilies would be a good addition, but there should be at least percentages for the major superfamilies to better describe the TE diversity.
We thank the reviewers for this suggestion to include a detailed repeat annotation in the manuscript. We have included a table with a summary of the presence of the major repeat classes in the genome and also the superfamilies with the percentage of the genome covered by each superfamily presented (Table 3, page 9). Further we have included a new section in our methods “Detailed identification of repeat family diversity in the genome” to describe the use of the EDTA pipeline for the repeat annotations (lines 592-598).

Line 169: BLASTP is repeated.
We have now included parentheses where necessary to remove the appearance of the word BLASTP repeating in line 156.

Lines 212-220: It would be helpful if this was expanded for clarity. Is the claim here that there is homoeologous exchange with replacement or is there fractionation of the region post or with exchange?
We used the HMM to determine if there was evidence for exchange of chromosomal regions between the subgenomes. In particular we were interested in if there was evidence of reciprocal homoeologous exchange as this would not be evident in the synteny plots and we did not want to misassign genes to the wrong subgenome for downstream analysis. We provided a clearer description to further describe why we conducted the analysis line 191-202.

Line 305: Probably better to say "suggest that" instead of "revealed how".
We thank the reviewer for this suggestion. We have now amended the sentence as suggested in line 295.

Line 308: Providing the densities of transposons per subgenome, especially in regard to proximity to gene space would be very helpful in understanding the relative subgenome dominance/transposon interaction. Other studies have suggested that higher TE abundance/density near genes results in a locus (and cumulatively subgenome) being dominated. It is curious if that is the case here or not. More relative representation of some elements does not exactly tell us this.
We thank the reviewer for this interesting and thoughtful suggestion. In order to address this suggestion we have now calculated the TE density flanking genes using a software pipeline called TE density (Teresi et al., 2022). Further we tested if there is a significant difference between the TE density near genes in the two subgenomes using non-parametric tests (Mann-Whitney U test) and failed to find any significant difference, although it was in the predicted direction with lower densities near genes in the dominant subgenome. Similarly we tested for any significant differences in TE density near genes retained as single copy and duplicate genes and did not discover any difference. The results of these new tests can be found from lines 263-283 in the manuscript while the new methods are found from lines 727-741.

Line 319: This is the description that needs to be used in lines 81-84.
We thank the reviewer for this suggestion to improve the clarity of the manuscript, as mentioned above we have now reworded the sentence referred as “line 81-84” now in “lines 62-68”.

Line 326: Use of "theory". It is a hypothesis.
Now we have replaced the word “theory” with the word “hypothesis”.

Line 331: Just check all use of the word "theory".
Now we have replaced the word “theories” with the word “hypotheses” throughout the

Powered by Editorial Manager® and ProduXion Manager® from Aries Systems Corporation

manuscript.

Line 361: "Subfunctionalization" is what I think you mean instead of "specialisation".
We thank the reviewer for pointing out this oversight. We have now replaced the word "specialisation" with the word "subfunctionalization" in line 348.

Line 368-369: "Andropogoneae"
We thank the reviewer for pointing out this oversight. As mentioned before, we have now checked the spellings of the word "Andropogoneae" all throughout the manuscript and have fixed any spelling errors where necessary.

Line 386: Some discussion on the previous suggestion of B. decipiens being a diploid species of the BCD clade is warranted. How does this compare to the paleopolyploidy of B. decipiens. Though it is a paleopolyploid, B. decipiens has maintained a lot of its genes in paralog copies making it very much a polyploid species. I am curious how this is reconciled with previous literature.
Distinguishing between diploids and (paleo)polyploids can be ambiguous in flower plant families, many of which have a long history of repeated genome duplication followed by gradual diploidization. The base chromosome number of the group is 10 and we found previous literature addressing B. decipiens as an old world polyploid (De Wet et al., 1963) based on chromosome counts as it is n=20. We identified that the parental species speciated approximately 6 million years ago followed by a hybridization event ~3 million years ago. This genome history is very similar to the situation in Miscanthus sinensis (Mitros et al., 2020) and in both cases the subgenomes are sufficiently divergent that they are genetic diploids. In support of this we were easily able to resolve the subgenomes in our assembly based on their unique genetic composition and our unpublished population genomic analysis is also consistent with disomic inheritance of markers. However, have now stopped referring to the species as diploid and instead refer to it as a paleotetraploid and a "genetic diploid" to avoid confusion.

Line 427: Where did these 24 populations come from? It is unclear what they are doing in the paper other than serving as other sources for genome sizing.
We thank the reviewer for the opportunity to clarify this statement. These 24 populations were collected from different places from Victoria and NSW in Australia. Individual samples from these populations were used to test for ploidy to identify diploid individuals of B. decipiens, as the plant is very similar morphologically to one of its' close relatives Bothriochloa macra which is a neopolyploid species. We wanted to identify the species with the smaller and less complex genome, B. decipiens, for the sequencing and the assembly of the reference genome. We have now included a sentence on where these populations come from in the manuscript in lines 412-414 and also a small description on the reason for using all these populations is given from lines 414-417.

Line 429: Numbers less than ten should be written out: five instead of 5.
We have now replaced "5" with the word "five" in line 417.

Line 433: Missing one of the standards.
We have mistakenly written that three standards were used instead of two. Now we have corrected that mistake in line 419..

Line 434: Were the standards added to each of the Bothriochloa samples? This is a common practice for flow cytometry in plants but not clear from the text.
Our FCM method was standardised so that all samples were disrupted for the same amount of time, with the same amount of force and with the same volume of buffer, greatly decreasing preparation variability. Additionally, because we were sampling unknown samples (in fact, we did not even know at the time if the samples we were

measuring were diploid or polyploid), it would have been difficult to choose the correct standards to co-chop with because they could potentially overlap with our sample peaks. We did not directly add standards to the samples, but ran standards before and after runs to watch for shifts in fluorescence. This is stated in the line 432-434.

Line 444: I do not think propidium iodide needs to be capitalised.
We have now written the word “propidium iodide” without the unnecessary capitalization in line 430.

Line 458: Is the haploid not 1.28 Gb?
The haploid size estimation via flow cytometry was 1.25 Gb not 1.28 Gb. Further the size of the final assembly was 1.21 Gb (Table 1).

Lines 458-459: Is this known or speculation? Should probably remove.
This was not speculation as the species are virtually identical except for the ploidy and the anther number, which is very difficult to identify in the field. Furthermore, we have done flow cytometry, genetic analysis and morphological identification on many of these same populations to show that the polyploid is indeed B. macra. We removed “likely” in line 444 to eliminate the possibility that another species was the likely cause of the ploidy variation.

Line 464: Is there some indication of how Dovetail did the isolations? At least a general technique if proprietary?
We assume the reviewer is referring to the DNA extraction process by “how Dovetail did the isolations”. We have included the method that Dovetail followed in order to extract DNA from the samples that were shipped in from line 449-456. Further to improve the clarity of this section we reworded the sentence as “The tissue was then shipped to Dovetail Genomics for the completion of DNA extractions by using the following steps.” in line 449.

Line 469: What is G2? Is there a more descriptive name for this?
The term “G2” refers to a buffer solution used in DNA/RNA extractions to enhance the yield of extracted DNA/RNA. In order to improve the clarity we have now written G2 DNA enhancer buffer solution instead of G2 in line 454..

Line 471: Usually said as "library construction" even if there is more than one.
We have now written “library construction” instead of “library constructions” in line 466.

Lines 520-525: What are the threshold parameters for breaks and joins?
We added more details in lines 495-502.

Line 542: What parameters were used for Trinity?
Trinity was run by specifying the sequence type (seqType) as fastq(fq) and specifying the two paired end reads using the —left and –right options. We did not use any other specific parameters while running Trinity. Now we have explained this in the text in line 520-522.

Line 552: How were the false positives identified?
Common feature of false positives is that the alignment between them (presumed “LTRs”), often extends beyond the boundary of the “LTR”. So we retrieved the flanking sequences of the LTRs, and used Blast to see if the flanking sequences were also alignable, and if so the putative LTR candidates were excluded. This was done using custom perl scripts provided in the instructions that we followed for advanced repeat library construction for the MakerP pipeline. We have now reworded the sentence on identifying false positives to enhance the clarity of the manuscript in lines 534-538.

Line 558: What parameters were used for RepeatModeler?
Here we used default parameters for running RepeatModeler. First a database was generated using the unmasked sequences that were not masked as repeats in the earlier steps using “BuildDatabase” command and the resulting database was the only input to run RepeatModeler in this step. We have now written that a database created out of the unmasked sequences was used as an input file to run RepeatModeler in this

Powered by Editorial Manager® and ProduXion Manager® from Aries Systems Corporation

step in line 542.

Line 718: How was convergence in Tracer assured? What ESS values had to be reached?
We used Tracer v. 1.6 to visualise replicate analyses, and determined convergence when the joint density (posterior) of each replicate stabilised to a overlapping, stationary distribution. We continued analyses until the ESS reached at least 200. We have included this information in the manuscript in line 697-699.

Reviewer #3: (Remarks to the Author):

The manuscript "Genome assembly of an Australian native grass species reveals a recent whole genome duplication and biased gene retention of genes involved in stress response" assembled a genome of Bothriochloa decipiens with a total length of 1.2Gb and scaffold N50 of 42.637 Mb, and further found it is a diploidized allotetraploid by comparative analysis. The authors here mainly focused on the WGD event and sub-genome dominance, particularly in relation with stress responses (Duplicates were enriched for genes involved in transcription and response to external stimuli like drought) or improved adaptation of C4 species to the environment of Australia.

The data here, such as fig5 seemed not to support the point of stress responses very well.
We have now added Table 5 in results to support the point of stress response. Particularly in Table 5b we report a summary of the most significant GO terms (P-value ≤ 1 x 10-3) relating to transcription and external stress response among overrepresented genes in genes retained as duplicates as reported by the gene enrichment analysis. In this we report the terms: response to water deprivation, positive regulation of response to salt stress, negative regulation of abscisic acid signalling pathway, response to absence of light and response to cold. All these terms came up as significant GO terms (P-value ≤ 1 x 10-3) among genes retained as duplicates. Further we have now put Fig 5 in supplementary material as Figure S4 for further illustration of the point.

It is very interesting that Bothriochloa decipiens is a diploidized allotetraploid, instead of a diploid species thought originally. And the assembly of two subgenomes was a pretty good job without clear information of diploid ancestors. A question here is whether there is an even closer one other than sorghum.
We would like to acknowledge that there are closer diploid relatives than Sorghum to our study species B. decipiens, but Sorghum was the only closest diploid relative with a genome assembly and high quality genome annotations that was essential for the downstream comparative analyses conducted in this manuscript.

About the dominance of subgenome, since it might not be shared by all polyploids, any more evidence about how the stresses affected biased gene retention here in Bothriochloa decipiens?
We could not find published evidence showing how stresses have affected gene retention in B. decipiens. However in an unpublished population genomic analysis in B. decipiens individuals, loci that showed strong associations with environmental variation were more likely to be retained as duplicates in the genomes, lending further support to the common finding that loci involved in adaptation tend to be retained as duplicated. We note that we did not claim that the biassed gene retention among subgenomes is caused by stresses, but rather that genes retained as duplicated are more likely to be involved in adaptation. There could be equal loss of non-stress related genes between the subgenomes (no biassed fractionation) resulting in this pattern in gene function related to stress response in the retained duplicates. It would be interesting, however, to determine if there was an interaction between the parental genomes' stress response profile and genome dominance, but we do not have any expression data in this species to address this question.

What's the contig N50?
We have now included a new row of information presenting the contig N50 values of the genome assemblies in Table 1, the genome assembly statistic summary table.

Powered by Editorial Manager® and ProduXion Manager® from Aries Systems Corporation

	Could the authors explain why not use 3rd generation sequencing methods such as Nanopore, SMRT, but 10X ?<br>The sequencing was done at a time when long read sequencing was more expensive and error prone. In consultation with Dovetail, we decided that 10X was the best and most cost effective approach.<br><br>Line 38: Found evidence of biased fractionation and differences in the…, was subject missing here?<br><br>We corrected this typo.<br><br>There is a nice review about subgenome,"One subgenome to rule them all: underlying mechanisms of subgenome dominance", and it would be good to cite it here.<br>We have now cited this publication on subgenome dominance in lines 68,265 and 308.<br><br>Line 323: DNA methylation is known for its role in epigenetic gene silencing [52-54] and in 324 restricting TE activity. Wondering whether there were enough data here to justify the effects of DNA methylation of TE on biased gene retention/gene expression of subgenome?<br><br>We modified this section to simply point out the pattern and suggest further exploration (lines 312-318).
Additional Information:	
**Question**	**Response**
Are you submitting this manuscript to a special series or article collection?	No
**Experimental design and statistics**<br><br>Full details of the experimental design and statistical methods used should be given in the Methods section, as detailed in our Minimum Standards Reporting Checklist. Information essential to interpreting the data presented should be made available in the figure legends.<br><br>Have you included all the information requested in your manuscript?	Yes
**Resources**<br><br>A description of all resources used, including antibodies, cell lines, animals and software tools, with enough information to allow them to be uniquely identified, should be included in the	Yes

Methods section. Authors are strongly encouraged to cite Research Resource Identifiers (RRIDs) for antibodies, model organisms and tools, where possible.<br><br>Have you included the information requested as detailed in our Minimum Standards Reporting Checklist?	
**Availability of data and materials**<br><br>All datasets and code on which the conclusions of the paper rely must be either included in your submission or deposited in publicly available repositories (where available and ethically appropriate), referencing such data using a unique identifier in the references and in the "Availability of Data and Materials" section of your manuscript.<br><br>Have you have met the above requirement as detailed in our Minimum Standards Reporting Checklist?	Yes

Manuscript
Click here to access/download;Manuscript;Bdecipiens_genome_revision_fin

Genome assembly of an Australian native grass species reveals a recent whole genome duplication and biased gene retention of genes involved in stress response

Nissanka P. De Silva[1*], Christopher Lee[1], Paul Battlay[1], A. Fournier-Level[2], Joslin L. Moore[1,3], Kathryn A. Hodgins[1]

[1] School of Biological Sciences, Monash University, Clayton, Victoria, Australia, 3800. Email: nissanka.desilva@monash.edu

[2] School of BioSciences, University of Melbourne, Melbourne, Victoria, Australia, 3010.

[3] Arthur Rylah Institute for Environment Research, Heidelberg, Victoria, Australia, 3084.

* Corresponding author

Nissanka Prasangi De Silva [0000-0002-6122-7343];

Christopher Lee [0000-0003-1108-8514];

Paul Battlay [0000-0001-6050-1868];

Alexandre Fournier-Level [0000-0002-6047-7164];

Joslin L. Moore [0000-0001-9809-5092];

Kathryn A. Hodgins [0000-0003-2795-5213];

Abstract

Background

The adaptive significance of polyploidy has been extensively debated and chromosome level genome assemblies of polyploids can provide insight into this. The Australian grass, *Bothriochloa decipiens,* belongs to the BCD clade, a group with a complex history of hybridization and polyploid. This is the first genome assembly and annotation of a species that belongs to this fascinating yet complex group.

Findings

Using Illumina short reads, 10X Genomics linked reads and Hi-C sequencing data we assembled a highly contiguous genome of *Bothriochloa decipiens,* with a total length of 1,218.22 Mb and scaffold N50 of 42.637 Mb. Comparative analysis revealed that the species experienced a relatively recent whole genome duplication. We clustered the 20 major scaffolds, representing the 20 chromosomes, into the two subgenomes of the parental species using unique repeat signatures. We found evidence of biased fractionation and differences in the activity of transposable elements between the subgenomes prior to hybridization. Duplicates were enriched for genes involved in transcription and response to external stimuli, supporting a biased retention of duplicated genes following whole genome duplication.

Conclusions

Our results support the hypotheses of a biased retention of duplicated genes following polyploidy and point to differences in repeat activity associated with subgenome dominance. *Bothriochloa decipiens* is a widespread species with the ability to establish across many soil types, making it a prime candidate for climate change resilient ecological restoration of Australian grasslands. This reference genome is a valuable resource for future population genomic research on Australian

grasses.

Key words - genome assembly, annotation, paleo- allopolyploidization, whole genome duplication (WGD), biased fractionation

Background

Whole genome duplication (WGD), or polyploidy, occurs via the doubling of chromosomal material either involving one species (autopolyploidy) or via hybridization of two species (allopolyploidy). The polyploid origin of many plant species has long been recognized [1,2], but while polyploidy is commonly observed in angiosperms, its evolutionary importance has been controversial. Some studies support the hypothesis that polyploidy can drive rapid adaptive evolution [3,4], while others have argued that polyploidy has played a minimal role in evolution and contributed little to adaptation [5]. However, there is growing evidence that ancestral WGD events caused key changes in major angiosperm clades, supporting their successful diversification [6].

After a WGD event, various molecular changes occur to restore the diploid state (diploidization; [7–11] via genome rearrangement, gene loss and epigenetic change [7,12]. Genes that encode DNA repair mechanisms and organelles functions tend to revert to single copy status following WGD events [13,14]. However, other duplicated genes may be retained [15]. Several hypotheses explain the patterns of duplicated gene retention and their evolutionary fate. For instance, the gene balance hypothesis states that genes coding for products that are dose-sensitive are protected from fractionation because if fractionated, the stoichiometry of the products and other gene products that they interact with will be affected and may bring about negative or lethal effects to the organism [16]. These principles are thought to apply to genes responsible for controlling functions related to gene regulation such as transcription factors or kinases acting as hubs with the potential to control

entire gene networks [17,18].

Functionally distinct subgenomes may also be retained after WGD. Biased fractionation through the preferential loss of the duplicated genes from the same subgenome has been observed in many polyploids [19–22]. Gene expression also tends to be biased between the subgenomes with higher gene expression in the subgenome that experiences less gene loss [19,20,23,24]. The less fractionated and more transcriptionally active subgenome is referred to as the dominant subgenome, and this asymmetry frequently occurs in hybrids with divergent parental genomes [25,26].

Poaceae (grasses) is the most successful plant family in terms of occurrence, ecological dominance and species richness [27] and approximately 80% of species in this family are polyploid and all grasses are derived from a polyploid event called rho [28]. Our study species, *Bothriochloa decipiens* (NCBI:txid883134), belongs to the tribe Andropogoneae (subfamily Panicoideae). This tribe contains many ecologically and economically important species, and independent allopolyploidization events have been exceptionally frequent in this group [29]. *Bothriochloa decipiens* is part of a cosmopolitan grass genus [30] closely related to *Capillipedium* and *Dichanthium* (together referred to as BCD). These three genera have the ability to interbreed despite their morphological differences; the term compilospecies was coined to describe this type of hybrid species complex [31,32] and *Bothriochloa decipiens* may be a donor species to this compilospecies complex [33].

Here we report a chromosome-level genome assembly, annotation and comparative analysis of a species from the BCD clade, *Bothriochloa decipiens*. This is the first genome assembly and annotation of a species that belongs to this fascinating yet complex group. Our highly contiguous *B. decipiens* genome assembly showed clear evidence of recent paleo-polyploidy. Using repeat signatures diverged between putative homoeologous chromosomes we were able to organize

chromosomes into subgenomes, allowing estimation of the timing of the speciation event prior to the most recent allopolyploidization event in this species. We further describe signatures of biased fractionation between subgenomes, as well as biases in the functions of genes retained as duplicated or single copy. This genome reference will act as an important resource for population genomic analysis of the BCD clade and will aid our understanding of the rich history of allopolyploidy in this group and its evolutionary significance.

Analyses

Genome size estimation, genome assembly and transcriptome assembly

Using flow cytometry (FCM) (see methods) the haploid genome size of the accession COB1-7 was estimated to be 1.25 Gb (Giga base pairs). The genome was assembled combining assemblies from linked read sequencing (10X) with HiRise™ scaffolding using Chicago and Hi-C libraries (Dovetail Genomics; [34] Table 1). As *B. decipiens* is known to have a haploid chromosome number n=20 [35,36] and the final assembly had a L90=20 (Table 1), we assumed that these scaffolds represented the 20 haploid chromosomes of *B. decipiens*.

Table 1. Statistics of the *Bothriochloa decipiens* genome assembly

**Assembly statistics**	**10X**	**10X+Chicago**	**10X+Chicago+Hi C**
**Contig L50/N50 (number/size)**	19,068 scaffolds \ 80.27 kb (kilo base pairs)	19,068 scaffolds \ 80.27 kb	16,083 scaffolds\ 80.20 kb
**Scaffold L50/N50 (number/size)**	125 scaffolds \ 2.733 Mb (mega base pairs)	116 scaffolds \ 3.080 Mb	10 scaffolds \53.95 Mb

**Scaffold L90/N90 (number/size)**	1,808 scaffolds \ 0.023 Mb	616 scaffolds \ 0.090 Mb	20 scaffolds \ 42.637 Mb
**Largest scaffold size**	14.847 Mb	14.607 Mb	95.095 Mb
**Total number of scaffolds**	25,759	19,068	15,895
**Total genome size**	1,217.63 Mb	1,218.36 Mb	1,218.22 Mb
**% Gaps**	6.78	6.84	6.86
**BUSCO[†](*n*)**	39:200:13:3	43:200:9:3	45:200:8:2
**BUSCO[†] (%)**	15:78:5:1	16:78:3:1	17:78:3:0.8

[†]Number of BUSCO (Benchmarking Universal Single-Copy Orthologs) genes found in the assembly using the eukaryota odb9 dataset. Genes are split into four categories: complete and single-copy, complete and duplicated, fragmented, and missing, and reported respectively.

RNA-Seq data from two tissues (leaf and stem) was used to assemble the transcriptome of *B. decipiens* using Trinity v.2.8.5 (RRID:SCR_013048) [37]. The final transcriptome assembly contained 197,655 transcripts, with 104,784 Trinity annotated genes, with an average length of 1,062 bp and a N50 length of 1,677.

Genome annotation, functional annotation, quality validation and repeat identification

We identified 60,652 putative protein coding genes (Supplementary Table S1). This was done by running iterative runs of the genome annotation pipeline MAKER v.3.01.03 (MAKER, RRID:SCR_005309) [38] and training gene predictions using AUGUSTUS v.3.3.3 (RRID:SCR_008417) [39] and SNAP v.2013-11-29 [40]. Functional annotations for the predicted genes were done by searching against several databases (see methods) (Table 2). We identified 94.1% of the core eukaryotic genes amongst our annotated genes, 22.7% being single copy, 71.4% being duplicated and 2.4% fragmented compared to BUSCO markers present in the library "eukaryota_odb10.2020-09.10". Of the total genome assembly, 57.88% corresponded to repetitive elements (Table 3). Repeated elements annotation was done using EDTA v.2.0.0 (RRID:SCR_022063) [41] (see methods). The majority of the repeated elements were long terminal repeats (LTRs) (39.51%). We also observed a quite large proportion of helitrons (10.35%) being present in the genome. A detailed summary of the TE annotations is provided in Table 3. Analysis of the *B. decipiens* repeat and gene density across each chromosome, reveals that gene density was low towards the centre of each scaffold, where repeat density was high (Figure 1).

Table 2. Summary of functional annotation for protein coding genes.

**Database**	**Number of gene models with annotation**	**Percentage of gene models with annotation (%)**
UniProtKB/Swiss-Prot	42,417	69.93
Tair10	49,099	80.95
Pfam	43,444	71.62
KEGG (Kyoto Encyclopedia of Genes and Genomes) orthology	17,465	28.79
KEGG orthology - E.C (Enzyme Commission) number annotations	8302	13.68

Table 3. Summary of length of genome sequence repeat-masked by repeat element class.

Major repeat class	Superfamily	Mbp present	% Of genome
Long terminal repeats (LTRs)	Copia	76.29	6.72
	Gypsy	315.39	27.80
	Unknown	56.62	4.99
Terminal inverted repeats (TIRs)	CACTA	28.09	2.48
	Mutator	20.17	1.78
	PIF Harbinger	8.19	0.72
	Tc1 Mariner	12.62	1.11
	hAT	6.60	0.58
	polinton	0.04	0.00
Non-LTR	LINE element	0.62	0.05
Non-TIR	helitron	117.49	10.35
Repeat region		14.59	1.29

Synteny and whole genome duplication

In order to determine if *B. decipiens* had undergone a whole genome duplication, a reciprocal BLASTP (Basic Local Alignment Search Tool Program) (RRID:SCR_001010) [42] was conducted using *B. decipiens* protein sequences as the query against themselves and homoeologous scaffolds were identified using the collinear blocks obtained via MCScanX (RRID:SCR_022067) [43]. This was also corroborated by the alignment against themselves of the 20 largest scaffolds (> 40Mb; representing 1.11 Gb of the 1.22 Gb genome) using Minimap2 v.2.1.8 (RRID:SCR_018550) [44] (Supplementary Figure S1). Of these 20 scaffolds, ten pairs (with more than 50% matching across both scaffolds) were identified as homoeologous (Supplementary Figure S1). Similarly, we identified collinear blocks between the two putative subgenomes of *B. decipiens* and *Sorghum bicolor* (the closest diploid relative with a high quality genome assembly) by conducting a reciprocal BLASTP [42] comparing protein sequences from each species using MCScanX [43]. We identified 33,146 *B. decipiens* genes that were orthologous to 19,611 *S. bicolor* genes across syntenic blocks. A relatively recent paleo-polyploidization event was evident as each chromosome from *S. bicolor* almost completely aligned to a pair of *B. decipiens* scaffolds as seen in the dot plot in Figure 2B. Further, these pairs of *B. decipiens* scaffolds show large syntenic blocks of duplicated genes as seen in the dot plot in Figure 2A. There was also some evidence of rearrangements between the subgenomes: for example, a translocation from scaffold 18 (homoeologous to scaffold 10) to scaffold 8 which appears to have regions from both subgenomes as a result (Figure 2). Therefore, this translocation likely occurred after the allopolyploidization event. Translocations are apparent in the *B. decipiens* genome alignment against itself (Supplementary Figure S1) and also in the syntenic relationship between the *B. decipiens* chromosomes when all 20 of them are aligned against themselves (Supplementary Figure S2). Other structural changes can be observed, including several inversions, clearly

identifiable on scaffold 13 when compared to scaffold 15 or to chromosome six in *S. bicolor* (Figure 2 (B)).

Genomic exchange across subgenomes and homoeologous pairs/chromosomes

The difference in the distribution of repetitive elements between pairs of homoeologous chromosomes can provide evidence of subgenome ancestry [45]. Diagnostic repeat signatures are found on one of each homoeologous pair of chromosomes, and they represent the remains of mobile elements with different activity in the diploid ancestors before the merging of the two genomes [45]. As there is no genomic data from any close diploid relative that does not share the most recent allopolyploidization event, we clustered the putative homoeologous chromosomes based on repeat abundance using kmer distributions. We partitioned the *B. decipiens* genome into subgenomes A and B by modifying the methods described in [46]see methods). We found 919 13-mers (13-bp sequences) occurring at least 100 times across the whole genome and also were at least threefold-enriched in one of the homoeologous chromosomes relative to the other. Based on the consistent enrichment for these 919 13-mers along the putative homoeologous chromosomes, each scaffold of a pair was assigned to a subgenome (Figure 3). The A group was defined based on the excessive abundance of 773 13-mers, and the B group based on the abundance of the other 146 13-mers (Figure 3). We then computed the densities of A- and B-preferred 13-mers across the scaffolds (Supplementary Figure S3A&B) and identified potential homoeologous exchange between subgenomes. Scaffold 8 from the B subgenome had a high density of subgenome A-preferred kmers at one end of the scaffold (Supplementary Figure S3B) consistent with the observation of a translocation from the dot plots (Figure 2). We then tested for homoeologous exchange across the subgenomes using a Hidden Markov Model implemented (HMM) in the R\HMM package [47]. We did this to determine if there was evidence for exchange of chromosomal regions between the subgenomes. In particular, we were interested in evidence of

reciprocal homoeologous exchange as this would not be apparent in the dot plots (e.g., Figure 2). However, we did not find evidence of reciprocal homoeologous exchange. For each chromosome clustered into the A or B subgenome, only rare instances of assignment to the alternate subgenome by the HMM occurred (except scaffold 8 mentioned above). Specifically, alternative assignment occurred in three regions (scaffold 9, 12, and 16), but these were regions with few A and B preferred kmers, and therefore sampling error would be more likely to contribute to misassignment to subgenomes using the HMM based approach. Also, these instances did not reflect reciprocal exchanges between the subgenomes. Impacts of these ambiguities in subgenome assignment were examined by including and excluding these regions in downstream analyses relying on subgenome assignment (i.e., biased fractionation).

We examined subgenome enriched LTRs (long terminal repeats), as differences in LTR activity in parental species can help differentiate subgenomes and can be used to assess the timing of allopolyploidy. We identified LTR repeats that belonged to nine LTR families that were at least three times more common in one subgenome. A or B-preferred kmers also overlapped with these repeated elements. There were eight LTR subfamilies (Grande1_ZM_pol/Gypsy, RIRE2_pol/Gypsy, Copia-11_SB/Copia, Copia-73_Mad/Copia, Copia-13_SB/Copia, SZ7_pol/Gypsy, CRM/Gypsy, Atlantys_OS_polGypsy) identified in subgenome A and only one (Copia-9_SB/Copia) identified in subgenome B. Their genomic locations are shown in Supplementary Figure S3C&D.

The timeline of paleo tetraploidy

We used 1:1 orthologs identified using OrthoFinder v.2.3.8 (RRID:SCR_017118) [48] across members of the Andropogoneae tribe using *Panicum hallii* and *Setaria italica* as outgroups to identify the likely timing of the subgenome divergence in *B. decipiens* (see methods; Figure 4A). We found that the diploid progenitors of the allopolyploid ancestor of *Bothriochloa decipiens*

speciated approximately 5.8 MYA (Million years ago). Our tree also dated the divergence of the progenitors of *M. sinensis* to around 6 MYA.

Timing of subgenome-specific LTR expansion

The greater abundance of LTR subfamilies specific to one subgenome is a signal of mobile element activity unique to one of the diploid ancestors of the allopolyploid [45]. Therefore, dating the insertion events of the subgenome specific LTRs (see methods) can be used to provide a rough estimate of the timing of the hybridization event. We found that the subgenome-specific LTR activity began 2-3 MYA and peaked around 3.5 MYA (Figure 4B). Activity declined around 6 MYA (Figure 4B), and this coincides with the timing of the speciation event of the diploid ancestors prior to allopolyploidy event inferred through the phylogenetic analysis (Figure 4A). Overall, this analysis suggests that the diploid ancestors may have evolved independently for about 3-4 MYA before the hybridization event.

Biases in gene retention between subgenomes

We analysed the collinear blocks between *S. bicolor* and *B. decipiens* to assess differences in retention of duplicated genes between subgenomes. Subgenome-specific retention was inferred as the number of genes retained in a given subgenome divided by the number of inferred ancestral (i.e., pre duplication) gene numbers. Collinear blocks obtained from the two independent scanning methods McScanX [43] and OrthoFinder v.2.3.8 [48] were used in two independent analyses to confirm patterns of fractionation (see methods). We then used a two-sided Fisher's exact test to determine if there was a significant difference in retention of genes between the subgenomes under the null hypothesis that gene loss between subgenomes was random. The percentage of genes retained was significantly higher in subgenome A compared to subgenome B (McScanX, Fisher's exact test, *P*-value = 2.2×10^{-16} and OrthoFinder, Fisher's exact test, *P*-value = 1.63×10^{-10} (Table 4).

Table 4. Subgenome-specific gene retention

**Clustering Method**	**Ancestral genes (retained + single)**	**Ancestral genes retained on A**	**Ancestral genes retained on B**	**Ancestral genes retained on A+B**	**Percent retained on A (%)**	**Percent retained on B (%)**	**Percent retained on A+B (%)**
**McScanX**	19,523	16,666	15,464	12,195	85	79	62
**OrthoFinder**	15,262	12,716	12,278	9,620	83	80	53

Differences in the function of duplicated genes retained after the WGD compared to those returning to single copy was tested through gene ontology enrichment analysis using the R\topGo package (RRID:SCR_014798) [49]. All single-copy genes in A or B, or duplicated genes in both A and B were used as foreground genes and the remaining ancestral genes (retained duplicated or single copy) as background genes. GO (Gene ontology) terms related to organelle functions such as chloroplast organization, chloroplast RNA modification and regulation of mitochondrion organization were among the most significant terms associated with genes returning to single copy (Table 5a, Supplementary Figure S4A). The most significantly enriched terms for genes maintained as duplicates were those relating to transcription, including positive and negative regulation of transcription, and terms related to external stress responses such as response to water deprivation and salt stress (Table 5b, Supplementary Figure S4B).

Table 5. Summary of the gene enrichment analysis. a) Summary of the most significant GO terms (Fisher's exact test *P*-value $\leq 1 \times 10^{-3}$) relating to RNA modification, transmembrane activity and organelle functions among overrepresented genes in genes retained as single copies as reported by the gene enrichment analysis. b) Summary of the most significant GO terms (Fisher's exact test *P*-value $\leq 1 \times 10^{-3}$) relating to transcription and external stress response among overrepresented genes in genes retained as duplicates as reported by the gene enrichment analysis

a)

**GO ID**	**Term**	**Annotated**	**Significant**	**Expected**	**P-value**
GO:0009451	RNA modification	451	128	74	3.6×10^{-12}
GO:0120009	Intermembrane lipid transfer	14	11	2.26	3.3×10^{-7}
GO:0009658	Chloroplast organization	442	92	71.26	2.1×10^{-4}
GO:0071712	ER (endoplasmic reticulum)-associated misfolded protein catabolism	23	11	3.71	3.0×10^{-4}
GO:1900865	Chloroplast RNA modification	53	22	8.55	3.3×10^{-4}

GO:0010821	Regulation of mitochondrian organization	17	10	2.74	4.5×10^{-4}

b)

GO ID	Term	Annotated	Significant	Expected	P-value
GO:0045893	Positive regulation of transcription	962	550	455.35	7.4×10^{-18}
GO:0045892	Negative regulation of transcription	593	333	280.69	7.0×10^{-9}
GO:0006355	Regulation of transcription	3176	1759	1503.31	1.9×10^{-8}
GO:0006357	Regulation of transcription by RNA polymerase	836	456	395.71	6.5×10^{-8}
GO:0009414	Response to water deprivation	963	460	455.82	1.7×10^{-6}
GO:1901002	Positive regulation of response to salt stress	84	60	39.76	2.4×10^{-6}

GO:0009788	Negative regulation of abscisic acid signalling pathway	107	70	50.65	4.6×10^{-5}
GO:0009646	Response to absence of light	88	60	41.65	6.1×10^{-5}
GO:0009409	Response to cold	689	371	326.13	3.8×10^{-4}

TE density between the subgenomes

TE density is known to be lower in genic regions for the dominant subgenome in some other neo and paleopolyploids [25,26,50], therefore we analyzed the TE density in regions flanking genes to test if a differences in TE density exists between the two subgenomes and between single copy and duplicated genes identified in the biased gene retention analysis. This is thought to reflect increased downregulation of genes owing to methylation of nearby TEs [51,52]. The density of TEs with 5000 bp of genes was not significantly different between the subgenomes upstream of the genes (Mann-Whitney U Test, *P*-value = 3.3×10^{-1}) (Table 6a) and also downstream of genes (Mann-Whitney U Test, *P*-value = 1.4×10^{-1}) (Table 6a) of genes. The TE density flanking single copy and duplicated genes was also not significantly different for the upstream of genes (Mann-Whitney U Test, *P*-value = 2.6×10^{-1}) (Table 6b) or downstream of genes (Mann-Whitney U Test, *P*-value = 4.5×10^{-1}) (Table 6b) of genes.

Table 6. Summary of TE density in flanking regions of genes. a) Genome specific TE density flanking genes (within 5000 bp for the start or end of the gene). b) TE density distribution with relation to the location of genes near single copy genes and genes retained as duplicated (within 5000 bp of the start or end of the gene).

a)

Subgenome	Mean TE density to the upstream of the genes	Mean TE density to the downstream of the genes
A	0.374 ± 0.01	0.375 ± 0.01
B	0.382 ± 0.01	0.383 ± 0.01

b)

Gene type	Mean TE density to the upstream of the genes	Mean TE density to the downstream of the genes
Single copy	0.362 ± 0.008	0.357 ± 0.007
Duplicated	0.347 ± 0.005	0.349 ± 0.005

Discussion

Here we report the chromosome-level genome assembly of *Bothriochloa decipiens,* a native Australian grass species important in grassland rehabilitation. Our comparative analysis revealed that this species is a paleo-polyploid, consistent with previous phylogenetic analysis of the group [29]. Our assembly and comparative analyses revealed a relatively recent whole genome duplication. Although the diploid progenitors are unknown, our clustering based on unique repeat signatures grouped the chromosomes into subgenomes. Phylogenetic analysis suggested that the diploid ancestors of the paleo-allopolyploid *Bothriochloa decipiens* speciated approximately 5.8 MYA. Additionally, we showed evidence of biased fractionation with significantly higher gene retention in one of the subgenomes. This subgenome also appeared to have had more active LTRs just prior to the allopolyploidy event. Consistent with expectations, genes that were retained as duplicates following the WGD event were enriched for functions involving transcription and stress response.

Patterns of gene loss and retention following allopolyploidy

The two subgenomes show asymmetric gene loss, with subgenome A retaining more genes than subgenome B (Table 4). This 'biased fractionation' commonly occurs after allopolyploidization [53] and has been observed in *Arabidopsis* [21], maize [22,54], and *Brassica* [19], although it is not always observed [55]. Biased fractionation can be a result of genome dominance, where gene expression tends to be higher in one subgenome compared to the other, leading to greater gene loss in the subgenome with reduced expression [19,20,23]. Unlike some previous studies [25,26,50], we did not find a difference in the density of TEs flanking genes between the subgenomes. Differences in the density of methylated TEs nearby genes is thought to contribute to biased fractionation in some paleopolyploids [24,56,57]. DNA methylation is known for its role in epigenetic gene silencing [51,52,58] and in restricting TE activity [59]. Interestingly, subgenome A also appears to

have had more active LTRs at the time of the most recent allopolyploidization event, as evidenced by the greater number of diagnostic kmers and LTR families associated with subgenome A (Supplementary Figure S3). It would be interesting to explore if the higher LTR activity prior to the WGD (Figure 4B) and the dominance of subgenome A (Table 4) were related. More retained genes and more active TEs have also been observed in the dominant subgenome of *Miscanthus* [46], but further investigation into the relationship between TE activity and genome dominance is warranted.

Functions overrepresented among the genes retained as duplicates, and the genes retained as single copy in the two subgenomes are aligned with well-established hypotheses and empirical work [60–64]. Genes containing domains responsible for functions such as RNA modifications and transmembrane activity tend to revert to singletons in plants [63]. Congruently, we identified genes related to RNA modification and transmembrane activity in our GO enrichment analysis of single copy genes (Table 5a, Supplementary Figure S4A). We also identified enriched GO terms related to organelles such as chloroplast, mitochondria and endoplasmic reticulum in the single-copy gene set. Genes encoding functions related to organelles are commonly retained as single copies [61]. The deleterious effect of altering the gene dosage balance could explain the retention of single copy genes responsible for organelle-mediated processes such as photosynthesis in chloroplasts and respiration in mitochondria. These functions involve proteins from both the organelle and nuclear genome. The interactions between these non-nuclear genomes are regulated to maintain the balance of the protein products created from them [65–67]. During a WGD, this balance could get affected as only the nuclear genome is duplicated, not the organelle's [14]. Alternatively, biased fractionation may preserve coadapted genes [68]. Nuclear encoded genes performing organelle functions and organelle genes have coevolved in each ancestral genome separately, and biased gene loss and reversion to single copy might maintain coadapted gene complexes and prevent negative interactions of genes between the subgenomes [68]. Alongside genes reverting to single copies, many genes were retained as duplicates. Genes coding for

subunits of multimeric proteins or complexes, transcription factors and signal transduction mechanisms are biased to avoid fractionation [60,62,64]. GO terms related to transcription, signal transduction and protein biosynthesis were over-represented amongst retained duplicate genes (Table 5b, Supplementary Figure S4B)). The gene dosage hypothesis explains these patterns of biased retention [16]: if either gene copy that codes for a dosage-sensitive gene product that interacts with other gene products is lost, the dosage imbalance may be deleterious to the organism. We also found that retained duplicated genes had an overrepresentation of GO terms related to response to external stressors like water deprivation, salt stress and response to abscisic acid signalling (Table 5b, Supplementary Figure S4B). Genes that give plants the ability to respond to various environmental stresses are frequently retained as duplicates after WGD [69]. As neofunctionalization and subfunctionalization can be possible fates of genes retained as duplicates [70,71], retention of such genes may promote adaptive evolution to abiotic stresses [72–75]. Multiple paleo polyploidization events that occurred independently throughout the history of evolution of angiosperms may have promoted the diversification of this clade across a wide range of environmental conditions by contributing to adaptation to new environments and stress [76].

The evolutionary history of the Andropogoneae

Species in the Andropogoneae clade, which *B. decipiens* belongs to, are dominant in modern day C4 grasslands. Most allopolyploidization events in the Andropogoneae occurred recently, in the late Miocene [29], which coincides with the expansion of C4 grasslands [77]. Our phylogenetic analysis suggests the speciation event leading to the ancestral genomes occurred at the end of the Miocene, approximately 5.8 MYA, which corresponds to other estimates reported for *Bothriochloa spp*. [29]. It also appears to have occurred at a similar time as the speciation event leading to the subgenomes A and B in *Miscanthus* [46]. For *B. decipiens* the insertion times of the subgenome-specific LTRs suggest that these species were diverged for up to 4 million years

before the hybridization event, although the date estimates are prone to error due to substitution rate variation among LTR families [78].

The genus *Bothriochloa* belongs to a group known as the BCD clade along with the two other genera, *Capillipedium* and *Dichanthium* [79]. Species in this clade are able to interbreed even though they are morphologically diverged. *Bothriochloa bladhii* has been identified as a compliospecies, which is a species able to absorb genomes from different species in the BCD complex [29,79]. However, *B. decipiens* is not included in the early studies on the relationships among the BCD clade [31]. Interestingly, a more recent phylogenetic study of species from the BCD clade in Australia suggested that *B. decipiens* may be an ancestor of this compliospecies complex because it had direct phylogenetic relationships with other species in the BCD clade, including *Bothriochloa bladhii*, making *B. decipiens* a key species to further our understanding of the evolution of this group [33]. Due to advances in sequencing technology and assembly methods, we now have the opportunity to assemble large and complex genomes to chromosomal levels and further our understanding of the evolution of such diverse and complex groups as the BCD clade. Our high-quality reference genome should spur future comparative genomic studies of allopolyploidy and hybridization in this clade and in grasses more generally.

Most allopolyploidization events in the Andropogoneae are recent, such as the one we report in *B. decipiens*. These events are concurrent with the expansion of C4 grasslands, and some studies argue that the allopolyploidy gave these grasses the ability to adapt to new environments, thereby enabling successful establishment and expansion [80,81]. The biased retention of duplicated genes related to stress response provides support for this hypothesis, however a causal relationship between allopolyploidization and C4 grassland expansion remains to be established. Future analysis examining the adaptive significance of retained duplicates using both comparative and population genomic approaches across a greater number of taxa, including groups with frequent WGD such as the Andropogoneae, will further our understanding of the adaptive significance of allopolyploidy

and its potential role in niche expansion for C4 grasses.

Potential implications

This genome will be an important resource for population genomic studies involving native grasses in this genus. Such analyses will shed light on the adaptive genetic landscape of these important foundation species, which could play a critical role in the development of climate change resilient grassland restoration practices in Australia and elsewhere [82,83]. Further this genome will be important in broader comparative analyses of the Andropogoneae which should provide greater insight into the evolutionary significance of allopolyploidy.

Methods

Species description

Bothriochloa decipiens (blue pitted grass) is a warm season, perennial, tufted grass that can grow up to a meter in height [84]. Due to its ability to establish well from direct seeding on many soil types, and the ability to withstand pressure caused by overgrazing, it has become an important species for rehabilitation. It is widespread in subtropical New South Wales (NSW) and Queensland as well as tropical Queensland [85]. It is a close relative, and phenotypically similar to, the polyploid *Bothriochloa macra,* which is a widespread native grass species in south-eastern Australia. The sporophytic chromosome number of *B. decipiens* is reported to be 2n=40 [86] and it is a genetic diploid.

Sample collection

The seeds used to grow the diploid *B. decipiens* accession COB1-7 used in this study were

collected from Cobbitty, NSW (34°03'N, 150°68'E). Using these seeds, a plant was grown and maintained at Monash University, Clayton to obtain leaf and inflorescence tissue samples for DNA and RNA extractions for the study.

Flow cytometry

We used FCM to estimate the genome size and predict the relative ploidy of 24 populations of *Bothriochloa macra* and *Bothriochloa decipiens* collected from different locations in states Victoria and NSW, and also sought evidence for within population variation in ploidy. Estimating ploidy from different populations was necessary as *B. decipiens* and polyploid *B. macra* are extremely similar morphologically and ploidy is the best method to reliably distinguish the two species [33]. We estimated the ploidy of at least five plants from each population following a modified plant FCM protocol [87]. Leaf samples from each population were collected from greenhouse grown plants and immediately placed on ice for same-day cytometric analysis. Two DNA genome size standards were selected, *Solanum lycopersicum* (2C = 1.96) and *Pisum sativum* (2C = 9.09) and grown from seed. Approximately 40 mg (milligram) of fresh leaf material was used for each sample, and placed into a 2.0 mL (millilitre) tube with a single 3 mm (millimetre) tungsten carbide bead and 436 µL (microliters) of an ice-cold nuclei suspension buffer modified from de Laats buffer (1984): 15mM (millimolar) HEPES, 1mM EDTA, 0.2% (v/v) Triton X-100, 80mM KCl, 20mM NaCl, 300 mM sucrose, 0.5 mM spermine, 15mM β-mercaptoethanol, 0.25 mM PVP. Adjusted to pH7. Samples were placed in a Qiagen Tissuelyser II and ground for 24 seconds at 25 hertz, and then the sample rack was reversed and ground again. The homogenate was filtered through two layers of Millipore Miracloth (22-25 µm (micrometre) pore size) suspended in a 3-piece nozzle. One µL of 10 µg (microgram)/µL RNAse was added for every 100 µL of filtrate and incubated at 37℃ for 20 minutes. Fifteen µL of 0.1 µg/ 444 µL of propidium iodide station solution was added to the filtrate and samples were run on the BD Accuri ™ C6 Cytometer using the settings outlined in [88]. Internal standards were run separately on the cytometer at the

beginning and end of the session – no change in dye fluorescence was recorded over that period of time.

A total of 38 samples produced an observable signal in the FCM run. All samples, excluding standards, were run in a blind fashion so that prior knowledge of expected ploidy did not bias the identification of nuclei peaks. The 2C values were determined for all *Bothriochloa* samples by comparing the FL2-A value of the sample to the internal standards, *Solanum* and *Pisum*, which have a known 2C value of 1.96 and 9.09pg respectively [87]. The average 2C genome size of diploid and polyploid plants was 2.80 pg (range 2.56 –2.99 pg) and 5.38 pg (range 4.94 – 5.91 pg), respectively (Supplementary Figure S5). Only one population (COB1) consisted of diploid individuals, and all individuals from this population were tested to confirm our findings. The COB1-7 accession was 2.56 pg which leads to a haploid genome size estimate of 1.25 Gb (Supplementary Figure S6). The polyploid samples were the closely related and phenotypically similar *B. macra*.

DNA extraction

For DNA extraction, fresh leaf tissue was collected from diploid individual COB1-7, flash frozen in liquid nitrogen and stored at -80 ℃. The tissue was then shipped to Dovetail Genomics for the completion of DNA extractions by using the following steps. To obtain high molecular weight DNA for 10X Genomics linked read sequencing, 1.8g of leaf material was ground with mortar and pestle to a fine powder to which 200mL of prewarmed CTAB and 100µL BME was added. This was incubated at 68℃ for 15 minutes. Once incubated, a mixture of 2x phenol chloroform, 1x isoamyl and 0.7x isopropanol was added and centrifuged to form a pellet. The pellet was combined with 9.5 mL of G2 DNA enhancer buffer solution, 200µL protease and 19µL RNase. Again, the mixture was incubated at 50℃ for 1 hour. The precipitated genomic DNA was used in library construction.

10X Library preparation sequencing and 10X assembly

Genomic DNA (gDNA) with an adjusted concentration between 1.0 -1.25 ng/µL was used to prepare the whole genome sequencing libraries using the Chromium Genome Library and Gel Bead Kit v.2, Chromium Genome Chip Kit v.2, Chromium i7 Multiplex Kit and Chromium controller according to manufacturer's instructions (10X Genomics). Genomic DNA was combined with Master Mix, a library of Genome Gel Beads, and partitioning oil to create Gel Bead-in-Emulsions (GEMs) on a Chromium Genome Chip. The GEMs were isothermally amplified with primers containing an Illumina Read 1 sequencing primer, a unique 16bp (base pairs) 10X barcode and a 6bp random primer sequence. Bar-coded DNA fragments were recovered for Illumina library construction. The amount and fragment size of post-GEM DNA was quantified prior using a Bioanalyzer 2100 with an Agilent High sensitivity DNA kit. Prior to Illumina library construction, the GEM amplification product was sheared on an E220 Focused Ultrasonicator (Covaris, Woburn, MA) to approximately 350bp. Then, the sheared GEMs were converted to a sequencing library following the 10X standard operating procedure. The library was quantified by qPCR (quantitative polymerase chain reaction) with a Kapa Library Quant kit (Kapa Biosystems-Roche) and sequenced on a NovaSeq6000 sequencer (RRID:SCR_020150) (Illumina, San Diego, CA) with paired-end 150bp reads.

Chicago library preparation and sequencing

A Chicago (RRID:SCR_014941) library was prepared as described in [34]. Briefly, ~500ng of high molecular weight gDNA was reconstituted into chromatin *in vitro* and fixed with formaldehyde. Fixed chromatin was digested with DpnII, the 5' overhangs filled in with biotinylated nucleotides, and then free blunt ends were ligated. After ligation, crosslinks were reversed and the DNA purified from protein. Purified DNA was treated to remove biotin that was not internal to ligated fragments. The DNA was then sheared to ~350bp fragments and sequencing

libraries were generated using NEBNext Ultra II kit with Illumina-compatible indices. Biotin containing fragments were isolated using streptavidin beads before PCR enrichment of each library. The libraries were sequenced on an Illumina HiSeq X Ten (RRID:SCR_016385) to produce 467 million 2x150bp paired end reads.

Dovetail HiC library preparation and sequencing

A Dovetail HiC library was prepared as described in [89]. Briefly, for each library, formaldehyde was used to fix chromatin in the nucleus in place. Fixed chromatin was digested with DpnII, the 5' overhangs filled in with biotinylated nucleotides, and then free blunt ends were ligated. After ligation, crosslinks were reversed and the DNA purified. Purified DNA was treated to remove biotin that was not internal to ligated fragments. The DNA was then sheared to ~350bp mean fragment size and sequencing libraries were generated using NEBNext Ultra enzymes and Illumina-compatible adapters. Biotin containing fragments were isolated using streptavidin beads before PCR enrichment of each library. The libraries were sequenced on an Illumina HiSeq X Ten to produce 400 million 2x150bp paired end reads.

Genome assembly

The 10X sequence data were assembled *de novo* with Supernova (RRID:SCR_016756) [90]. This *de novo* assembly, along with Chicago library reads and Dovetail HiC library reads, was used as input data for HiRise (RRID:SCR_023037), a proprietary software designed specifically for using proximity ligation data to scaffold genome assemblies [34]. An iterative analysis was conducted. First, Chicago library sequences were aligned to the draft *de novo* assembly from Supernova using SNAP (RRID:SCR_007936) [91]. The separations of Chicago read pairs mapped to the draft scaffolds were analyzed by HiRise to estimate the genomic distance between read pairs, and the model was used to identify and break putative misjoins, score prospective joins, and make joins

above the default threshold. After aligning and scaffolding Chicago data, Dovetail HiC library sequences were aligned and scaffolded following the same method. After scaffolding using Chicago and HiC library data, visual inspection of the contact maps identified two misjoin events in the largest scaffold and one other scaffold (black circles in Supplementary Figure S7). Manual corrections were performed using link density plots in Juicebox (RRID:SCR_021172) [92] to make breaks within those two scaffolds and produce the link density plot for the final assembly (Supplementary Figure S8).

mRNA-seq library preparation

RNA was extracted separately from young (a few weeks) and old (a year) tissue (leaf and stem) from one individual using the Qiagen RNeasy kit. RNA was pooled and a library was synthesised and sequenced by Genewiz^{TM} on an Illumina Novaseq 6000 platform in 2x150bp mode, resulting in 64,756,621 reads.

Transcriptome assembly

Raw RNA-seq reads were first cleaned by trimming the adapters using Trimmomatic v. 0.38 (RRID:SCR_011848) with the parameter “ILLUMINACLIP:TruSeq3-PE.fa:2:30:10:2:keepBothReads LEADING:3 TRAILING:3 MINLEN:36” [93]. The trimmed reads was used to assemble the transcriptome using Trinity v.2.8.5 (RRID:SCR_013048) [37] using default parameters. Assembly statistics for the transcriptome assembly was obtained after running the TrinityStats.pl script on the reference transcriptome assembly fasta file (obtained from Trinity).

Annotation of repetitive sequences

A custom repeat library was constructed following recommendations of the MAKERP pipeline for advanced repeat construction [94]. Both structure-based and homology-based approaches were used to increase the power to detect repeats. Sequences of miniature inverted repeat transposable elements (MITEs) were collected using MITE-Hunter (RRID:SCR_020946) [95] using all the default parameters. Long terminal repeat retrotransposons (LTRs) were collected using LTRharvest (RRID:SCR_018970) and LTR-digest [96,97]. The candidates were filtered for false positives caused by other tandem repeats such as centromeres, tandem gene duplications or other transposable elements. A common feature of these sequences is that the alignment between the two putative “LTRs”' often extend beyond the boundary of the “LTR” into the flanking region, so these false positive candidates were excluded by identifying those sequences whose alignments extend beyond the LTR boundary into the flanking region. Representative sequences (exemplars) were chosen as described previously [54] to reduce the redundancy of the LTR. Then other repetitive elements were collected by first masking the genome sequence with the previously obtained MITE and LTR sequences. Then unmasked sequences were extracted and processed by RepeatModeler v.2.0.3 (RRID:SCR_015027) [98] by providing a database built out of the unmasked sequences as an input, to identify additional repeats. As many repeats carry gene fragments, all the collected repetitive elements were searched against a plant protein database that contains those from swissprot plant protein and NCBI (National Centre for Biotechnology Information) Refseq plants [99] with transposon proteins excluded. Elements with significant hits to genes were removed along with 50bp upstream and downstream of the hit. If the remaining sequence was less than 50bp then it was completely excluded. Sequences matching the plant proteins as well as 50bp of flanking sequences were removed using the package ProtExcluder [94]. After this if the remaining portion of the sequence was shorter than 50bp, the entire sequence was excluded. Sequences of all the identified repetitive elements were joined together to form a final custom repeat library to be used to mask the repetitive elements of the genome in the Maker genome annotation protocol [38].To

identify the type of repeat (including the repeat family), the unidentified repeats from RepeatModeler, as well as the LTRs and MITES from the custom library were searched against two transposase databases. The first was Tpases020812 [100]. This database is composed of transposase protein sequences from the RepeatMasker v.4.1.1 (RRID:SCR_012954) [101], and from two other sources [102,103], and was searched using BLASTX (RRID:SCR_001653) [104]. The second was the publicly available Dfam-curated library of repeats [105], which was searched using Hmmer v.3.3.1 (RRID:SCR_005305) [106] implemented through [107]. The sequences that matched the database were classified according to their top hits. Using this custom repeat library, RepeatMasker version 4.1.1 [101] was used to mask the genome and identify the distribution of repeat types.

Genome annotation

MAKER v.3.01.03 (RRID:SCR_005309) [38] genome annotation pipeline was used to annotate the genome. The input files provided for the first run were the genome assembly fasta file (Genbank accession JALGXP000000000), the reference transcriptome assembly fasta file (obtained from Trinity), and the protein homology evidence from a plant protein database [99] which combines the Swissprot plant protein database and NCBI Refseq for plants excluding transposable elements. Repetitive regions were masked using our custom repeat library. Additional regions with low complexity were soft masked using RepeatMasker v.4.1.1 [101]. Iterative runs of MAKER v.3.01.03 [38] were undertaken in order to train the gene predictors SNAP v.2013-11-29 [40] and AUGUSTUS v.3.3.3 [39] as recommended by [38]. The first round of annotation was based on alignments of the transcriptome to the genome. For the first round the est2genome option in the Maker control file was set to one to allow Maker to infer gene models directly from the RNA-seq evidence in the transcriptome. After the completion of the first round of annotations, gene models with an AED (Annotation Edit Distance) score of 0.25 or greater and a length of 50 or more amino acids were retained and used to train SNAP v.2013-11-29 [40] to obtain a SNAP hmm

file. We then trained AUGUSTUS v.3.3.3 [39] using BUSCO v.3.0.2 (RRID:SCR_015008) [108]. First, training sequences were identified using the gene models predicted by Maker from the first run by excising regions with mRNA annotations and 1000 bp on either side. These were used to run BUSCO using the embryophyte set of conserved genes and an initial hmm model from rice. After training both SNAP and Augustus, Maker was run again, with SNAP hmm and Augustus files. A total of three rounds of training for each gene predictor were run. We used the script genestats [109] to calculate the numbers and lengths of genes, exons, introns and UTR (untranslated region) sequences present in the predicted gene models by the final Maker run (Supplementary Table S1). We ran BUSCO v.5.1.3 [110] with the eukaryota_odb10 lineage data set on the predicted transcript fasta file by Maker to assess the quality and the completeness of the annotated genome.

Detailed identification of repeat family diversity in the genome

Apart from constructing a repeat library to be used in the Maker genome annotation pipeline above, EDTA v.2.0.0 [41] was used to obtain more information about the repeat diversity in the genome. Input files used to run the EDTA pipeline for a more accurate repeat annotation were the genome assembly fasta file, fasta file with the coding sequences of *B. decipiens* obtained after the final annotation round of MAKER above and a BED file with the locations of genes in the genome as predicted by Maker annotation pipeline above.

Subgenome and homoeologous exchange identification

First, we identified the 20 largest scaffolds (> 40Mb; representing 1.11 Gb of the 1.22 Gb genome). These scaffolds were then aligned against themselves using Minimap2 v.2.1.8 (RRID:SCR_018550) [44] to identify scaffolds that shared homology and synteny that would indicate putative homoeologous chromosomes. The alignments were plotted using the R\pafr

package v.0.0.2 [111]. Of these 20 scaffolds, ten pairs (with more than 50% matching across both scaffolds) were identified as the pairs of homoeologous scaffolds (Supplementary Figure S1). As there is no genomic data from any close diploid relatives that do not share the most recent allopolyploidization event, we clustered the putative homoeologous chromosomes based on repeat abundance using kmer distributions. We partitioned the *B. decipiens* genome into subgenomes A and B by modifying the methods described in [46]. Specifically, we first identified 13 base pair sequences (13-mers) using Jellyfish v. 2.3.0 (RRID:SCR_005491) [112] and retained kmers at high abundance in the assembly (100 x or above). For each pair of scaffolds, we compared the counts of these 13-mers, identifying those that differed in abundance by three-fold or more between scaffolds. To control for any differences in scaffold length impacting this assessment, we further reduced the set of diagnostic 13-mers to those that retained a three-fold difference after standardizing kmer count for the scaffold length, while keeping only those diverging in the same direction as the absolute kmer count. Hierarchical clustering of scaffolds based on difference in 13-mer counts was used to identify putative subgenomes as implemented in the R\ComplexHeatmaps package (RRID:SCR_017270) [113].

We tested for homoeologous exchange among the subgenomes using a Hidden Markov Model implemented in the R\HMM package [47]. We used the most common kmer type (A or B) in 1Mbp windows as the observed states and the subgenome type for each of the 1,121 windows. The initial HMM used equal starting probabilities and transition probabilities of 0.01. We trained the HMM emission probabilities (viterbiTraining) using Scaffold 5 and Scaffold 15 as they appeared not to be subject to any subgenome exchange based on the A and B kmer density plots (Supplementary Figure S3).

We examined subgenome enriched LTRs, as differences in LTR activity in parental species can help differentiate subgenomes and can be used to assess the timing of allopolyploidy. LTRs were used for this because they are rapidly evolving, making it easy to differentiate between related

subfamilies. The timing of insertions can be calculated by examining the substitution rates for members of the same subfamily using the 5' and 3' regions [46]. Specifically, intact retrotransposons in the genome were identified using LTR-HARVEST [96]. The 'best' option was used for pairing overlapping LTR sequences, allowing the inner sequences of the retrotransposons to contain gaps. We performed an all-versus-all BLAST (RRID:SCR_004870) [42] on the long terminal repeat segments of the identified LTRs with an e-value cut off of 1e-2. Hits with the percentage of alignment between query and subject equal or greater than 90% over their entire length were selected. We then used MCL algorithm [114] to cluster the filtered blast alignments into retrotransposon subfamilies using an inflation parameter (-I) of 3. We counted the occurrence and the total base pairs that each LTR subfamily obtained from above clustering in the putative A and B subgenomes identified above. We identified LTR subfamilies that were three times more common in one of the subgenomes using both occurrence and bp count. Then we determined if these repeats overlapped with multiple A or B genome preferred kmers to confirm that kmers were representing longer repetitive sequences and to confirm that these kmers were marking repeat expansion that occurred just before the allopolyploidy event.

Gene function prediction

The predicted protein sequences obtained from the final run of MAKER were aligned to the UniProtKB/Swiss-Prot [115] and TAIR10 [116] protein databases using BLASTP (RRID:SCR_001010) [42] with an e-value cut-off of 1.0e-5. The GO term associated with the best hit for each BLASTP search was identified in each of the three databases above and assigned to the *B. decipiens* query. InterProScan v. 5.51-85.0 (InterProScan, RRID:SCR_005829) [117] was used to search the query protein fasta against the Pfam [118] protein family database and identify functional protein domains. Pfam accessions and GO terms were retrieved for the *B. decipiens* query sequences. The query protein sequences were BLAST (Basic Local Alignment Search Tool)

searched against the KEGG database [119–121] using the online tool KofamKOALA - KEGG orthology search [122] with an e-value cut-off of 1.0e-5. KEGG orthology terms and enzyme codes were retrieved for each hit.

Genome synteny and whole genome duplication

To determine if the *B. decipiens* genome had undergone whole genome duplication, a reciprocal BLASTP was conducted using *B. decipiens* protein sequences as the query against themselves with a minimum e-value greater than le-5. Then MCScanX [43] was used to identify syntenic blocks within the genome. The collinear blocks obtained via MCScanX between the putative A and the B subgenome of *B. decipiens* were visualised using Synvisio [123]. Similarly, we identified collinear blocks between the two putative subgenomes of *B. decipiens* and *S. bicolor* by conducting a reciprocal BLASTP comparing protein sequences from each species using MCScanX and plotted the results using Synvisio.

Estimating the timeline of paleotetraploidy

We estimated the timing of speciation events in the Andropogoneae using *Panicum hallii* and *Setaria italica* as outgroups. The reference gene sets for *Sorghum bicolor* v3.1.1, *Panicum hallii* v2.2, *Setaria italica* v2.1, *M. sinensis* v7.1 and *Zea mays* (B73 RefGen_v4) were downloaded from Phytozome v12.1 (Phytozome, RRID:SCR_006507) [124]. The *Saccharum spontaneum* reference gene set [125] was also downloaded. We separated the A and B subgenomes of *M. sinensis* (as identified in [46]), as well as those of *B. decipiens*, to compare the timeline of the paleopolyploidy events between *Miscanthus* and *B. decipiens* species. We identified 1:1 orthologs between all species (or subgenomes) using OrthoFinder v.2.3.8 (OrthoFinder, RRID:SCR_017118) [48]. A core set of 392 single-copy genes were retained and multiple sequence alignments were performed for each orthologous cluster using Genodup [126] and Mafft (Mafft, RRID:SCR_011811) [127]. Poorly

aligned regions were removed using Gblocks 0.91b (Gblocks, RRID:SCR_015945) [128] and the final alignments were concatenated into a single alignment.

Phylogenomic analysis

The best model of evolution was inferred using jModelTest2 [129,130]. A phylogenetic tree was constructed using RAxML (RAxML, RRID:SCR_006086) [131] with the GTRGAMMAI model of evolution and 1000 bootstrap replicates. *Setaria italica* and *Panicum hallii* were designated as outgroups.

Divergence date estimation

Divergence among lineages in the phylogeny were estimated from the concatenated alignment using BEAST v.2.5 (BEAST, RRID:SCR_010228) [132,133] after using bModelTest [134] to infer the best substitution model. Parameters included the GTR (general time reversible) substitution model with unequal frequencies, four gamma categories, estimated shape and invariant sites. We chose a relaxed log normal clock with estimated clock rates. We set priors to constrain the estimated dates at the *Setaria-Panicum* (12.8-20 MYA), and the Andropogoneae (13-21.2 MYA) nodes, using a uniform distribution between the minimum age and maximum ages of divergence times obtained from the TimeTree database [135]. BEAST2 (BEAST2, RRID:SCR_017307) [132] analysis was conducted for 50 million generations, and logging at every 5000 trees. Convergence between runs was assessed with Tracer v.1.6 [136]. We used Tracer v. 1.6 to visualize replicate analyses, and determined convergence when the joint density (posterior) of each replicate stabilized to an overlapping, stationary distribution. We continued analyses until the ESS reached at least 200. Trees were summarised with TreeAnnotator [133] using a burn-in value of 20%.

Timing of subgenome specific LTR expansions

We aligned the long terminal repeats of each LTR family cluster using Mafft (Mafft,

RRID:SCR_011811) [127]. We computed Jukes-Cantor distance matrices using the R\ape package [137]. We estimated the divergence times of each LTR family as k/2r (k=divergence, r=substitution rate) [138]. We used 1.3 x 10^{-8} as the substitution rate per site per year [139].

Determination of biases in subgenome gene retention

We analysed the collinear blocks between *S. bicolor* and *B. decipiens* to assess differences in retention of duplicated genes between subgenomes. Subgenome-specific retention was inferred as the number of genes retained in a given subgenome divided by the number of inferred ancestral (i.e., pre duplication) gene numbers. Consequently, we calculated the number of ancestral (pre duplication) genes as those orthologous genes present in *S. bicolor* and in one or both of the two subgenomes. We then compared this number to the total number of genes present only in subgenome A, only in subgenome B or in both subgenomes. We then used a two-sided Fisher's exact test to determine if there was a significant difference in retention of genes between the subgenomes under the null hypothesis that gene loss between subgenomes was random. We also used the results from OrthoFinder (OrthoFinder, RRID:SCR_017118) to confirm this pattern. Specifically, we identified 1:1 and 1:2 orthologs between *S. bicolor* and *B. decipiens*, only retaining 1:2 orthologs that were mapped to chromosomes on both *B. decipiens* subgenomes.

Differences in the function of duplicated genes retained after the WGD compared to those returning to single copy was tested through gene enrichment analysis using R\topGo (topGO, RRID:SCR_014798) [49] where a Fisher's exact test was used compare the expected number of genes to the observed number of genes. All single-copy genes in A or B, or duplicated genes in both A and B were used as foreground genes and the remaining ancestral genes (retained duplicated or single copy) as background genes.

TE densities between the subgenomes

For this we used the software pipeline TE density [140]. Here TE density is defined as the number of TE-occupied base pairs in a given base pair range (window) divided by the window size. First we calculated the TE density relative to all the genes present in the genome by providing the gene annotation BED file obtained through the genome annotation by Maker and the TE annotation BED file obtained via the EDTA v.2.0.0 [41] pipeline. The window size was defined as 5000 bp for the calculations, although larger (50,000 bp) and smaller window size (500 bp) yielded qualitatively similar results. The program outputs calculated TE density value for both upstream and downstream of each gene. We then used a Mann - Whitney U test to determine if there was a significant difference between the average TE density in both up and down stream of genes found in the two subgenomes. We then investigated if there were any differences in TE density near genes identified as single copy or duplicated in the above analysis of biases in gene retention (McScanX scanning method). To do this we used a Mann - Whitney U test to determine if there was a significant difference between the average TE density in both up and down stream of genes found retained as single copy or as duplicated in the genome.

Availability of source code

R scripts used in this study can be found in the public github repository [141].

Data Availability

Raw reads for the genome assembly have been deposited under BioProject accession number PRJNA819081. Illumina library raw reads namely 10x, Chicago and HiC data have been deposited in the Sequence Read Archive (SRA) under study accession numbers SRR18458736, SRR18471564 and SRR18471563. RNAseq data have been deposited under SRA accession number SRR18471562. Genome assembly of *B. decipeins* is deposited in the NCBI genome database under the accession JALGXP000000000. All supporting data is available via the *GigaScience* repository, GigaDB [142].

Abbreviations

°C: degree Celsius, BCD : *Bothriochloa Capillipedium* and *Dichanthium*, BLAST : Basic Local Alignment Search Tool, BLASTP :Basic Local Alignment Search Tool Program, bp : base pairs, BUSCO : Benchmarking Universal Single-Copy Orthologs, DNA: Deoxyribonucleic Acid, FCM :flow cytometry, Gb : Giga bases, gDNA :genomic DNA, GO : Gene ontology, HMM :Hidden Markov Model, KEGG : Kyoto Encyclopedia of Genes and Genomes, LTR : long terminal repeats, Mb : mega base pairs, mg :milligram, MITE : miniature inverted repeat transposable element, mL :millilitre, mM : millimolar, mm : Millimetre, mRNA : Messenger RNA, MYA : Million Years Ago, NCBI : National Centre for Biotechnology Information, ng : nanogram, NSW : New South Wales, PCR : polymerase chain reaction, RNA : ribonucleic acid, Seq :sequencing, TE : transposable elements, UTR : untranslated region, WGD : Whole genome duplication, µg : microgram, µL : microliters, µm: micrometre

Competing Interests

The authors declare that they have no competing interests.

Funding

This study was supported by Hermon Slade Foundation - Grant number HSF1703, Monash Graduate Scholarship, Monash University, Monash International Tuition Scholarship, Monash University and Denis and Maisie Carr Award and Travel grant 2020, School of Biological Sciences, Monash University.

Author's Contributions

K. A. H. - conceived and designed the study. K.A.H., J.L.M. and A.F.L. - supervised the research, C.L. conducted the FCM, RNA extractions and phylogenetic analyses, K.A.H. and N.P.D. - annotated the genome, carried out the subgenome and homoeologous exchange identification, genome synteny and whole genome duplication analysis and timing of subgenome-specific LTR expansion, N.P.D. carried out repeat library construction, transcriptome assembly, N.P.D. and P.B. carried out functional predictions, determination of biases in subgenome gene retention. N.P.D and K.A.H. wrote the first draft of the manuscript. All authors approved the final version of the manuscript.

Acknowledgements

We thank Brendon Levot for collecting the seeds from which the individual specimen used in this study was grown from. Extraction of genomic DNA, preparation and sequencing of libraries and genome assembly was done by Dovetail Genomics. The preparation and sequencing of RNA-seq libraries was done by Genewiz. Permission to collect plant material for the study was granted by NSW National Park and Wildlife Service - Licence number- SL 799 102026.

Figure Legends

Figure 1. The *Bothriochloa decipiens* genome landscape. Location across the 20 chromosomes and distribution, in 1Mb windows of gene density (green track), repeat density (blue track), DNA-TE density (red track), LTR (long terminal repeats) -TE density (yellow track) and GC (guanine-cytosine) content (black track).

Figure 2 (A) The syntenic relationship between the pairs of homoeologous chromosomes in *B. decipiens*. (B) The syntenic relationship between the *B. decipiens* and *S. bicolor* orthologous genes. Each *S. bicolor* chromosome (so) shares synteny with a pair of *B. decipiens* chromosomes (bd) suggesting that the allotetraploid *B. decipiens* genome was formed by the hybridization of two *Sorghum*-like ancestors.

Figure 3. The differentiation of homoeologous pairs of chromosomes into subgenome A (blue) and subgenome B (red) based on the hierarchical clustering of Euclidean distances among scaffolds using counts of 13-mers.

Figure 4. (A) Phylogenetic tree (MYA) of the Andropogoneae showing the time (MYA) of divergence between the diploid ancestors of the allotetraploid *B. decipiens*. (B) Density distribution of divergence the time estimate for all LTR families with subgenome-specific expansion activity.

References

1. Grant V. Plant Speciation. 1981.

2. Ledyard Stebbins G. Variation and evolution in plants. Columbia University Press.1950.

3. Edger PP, Heidel-Fischer HM, Bekaert M, Rota J, Glöckner G, Platts AE, et al.. The butterfly plant arms-race escalated by gene and genome duplications. *Proc Natl Acad Sci U SA*. 2015;112:8362–8366

4. McCarthy EW, Chase MW, Knapp S, Litt A, Leitch AR, Le Comber SC. Transgressive phenotypes and generalist pollination in the floral evolution of Nicotiana polyploids. *Nat Plants*. 2016; 2:16119.

5. Stebbins GL. Processes of organic evolution. Second Edition. Prentice-Hall.1971.

6. Soltis PS, Soltis DE. Ancient WGD events as drivers of key innovations in angiosperms. *Curr Opin Plant Biol*. 2016;30:159–165.

7.Doyle JJ, Flagel LE, Paterson AH, Rapp RA, Soltis DE, Soltis PS, et al.. Evolutionary genetics of genome merger and doubling in plants. *Annu Rev Genet*. 2008; 42:443–461.

8. Freeling M. Bias in plant gene content following different sorts of duplication: tandem, whole-genome, segmental, or by transposition. *Annu Rev Plant Biol*. 2009; 60:433–453.

9. Lim KY, Soltis DE, Soltis PS, Tate J, Matyasek R, Srubarova H, et al.. Rapid chromosome evolution in recently formed polyploids in Tragopogon (Asteraceae). *PLoS One*. 2008; 3:e3353.

10.Mandáková T, Joly S, Krzywinski M, Mummenhoff K, Lysak MA. Fast diploidization in close mesopolyploid relatives of *Arabidopsis*. The Plant Cell. 2010;*22*(7), 2277–2290.

11.Tayalé A, Parisod C. Natural pathways to polyploidy in plants and consequences for genome re

organization. *Cytogenet Genome Res*. 2013;140:79–96.

12. Sémon M, Wolfe KH. Consequences of genome duplication. *Curr Opin Genet Dev*. 2007;17:505–512.

13. Conant GC. Comparative genomics as a time machine: how relative gene dosage and metabolic requirements shaped the time-dependent resolution of yeast polyploidy. *Mol Biol Evol*. 2014; 31:3184–3193.

14. De Smet R, Adams KL, Vandepoele K, Van Montagu MCE, Maere S, Van de Peer Y. Convergent gene loss following gene and genome duplications creates single-copy families in flowering plants. *Proc Natl Acad Sci U S A*. 2013;110:2898–2903.

15. Freeling M, Woodhouse MR, Subramaniam S, Turco G, Lisch D, Schnable JC. Fractionation mutagenesis and similar consequences of mechanisms removing dispensable or less-expressed DNA in plants. *Curr Opin Plant Biol*. 2012;15:131–139.

16. Birchler JA, Veitia RA. The gene balance hypothesis: implications for gene regulation, quantitative traits and evolution. *New Phytol*. 2010;186:54–62.

17. Rody HVS, Baute GJ, Rieseberg LH, Oliveira LO. Both mechanism and age of duplications contribute to biased gene retention patterns in plants. *BMC Genomics*. 2017;18:46.

18. Tasdighian S, Van Bel M, Li Z, Van de Peer Y, Carretero-Paulet L, Maere S. Reciprocally retained genes in the angiosperm lineage show the hallmarks of dosage balance sensitivity. *Plant Cell*. 2017; 29:2766–2785.

19. Cheng F, Wu J, Fang L, Sun S, Liu B, Lin K, et al.. Biased gene fractionation and dominant gene expression among the subgenomes of *Brassica rapa*. *PLoS One*. 2012;7:e36442.

20. Renny-Byfield S, Rodgers-Melnick E, Ross-Ibarra J. Gene fractionation and function in the

ancient subgenomes of Maize. *Mol Biol Evol*. 2017;34:1825–1832.

21. Thomas BC, Pedersen B, Freeling M. Following tetraploidy in an *Arabidopsis* ancestor, genes were removed preferentially from one homoeolog leaving clusters enriched in dose sensitive genes. *Genome Res*. 2006;16:934–946.

22. Woodhouse MR, Schnable JC, Pedersen BS, Lyons E, Lisch D, Subramaniam S, et al.Following tetraploidy in Maize, a short deletion mechanism removed genes preferentially from one of the two homoeologs. *PLoS Biol*. Public Library of Science. 2010;8:e1000409.

23. Garsmeur O, Schnable JC, Almeida A, Jourda C, D'Hont A, Freeling M. Two evolutionarily distinct classes of paleopolyploidy. *Mol Biol Evol*. 2014;31:448–454.

24. Schnable JC, Springer NM, Freeling M. Differentiation of the maize subgenomes by genome dominance and both ancient and ongoing gene loss. *Proc Natl Acad Sci U SA*.2011;108:4069–4074.

25. Alger EI, Edger PP. One subgenome to rule them all: underlying mechanisms of subgenome dominance. *Curr Opin Plant Biol*. 2020;54:108-113.

26. Edger PP, Smith R, McKain MR, Cooley AM, Vallejo-Marin M, Yuan Y, et al. Subgenome dominance in an interspecific hybrid, synthetic allopolyploid, and a 140-year-old naturally established neo-allopolyploid monkeyflower. *Plant Cell*. 2017;29:2150–2167.

27. Linder HP, Lehmann CER, Archibald S, Osborne CP, Richardson DM. Global grass (Poaceae) success underpinned by traits facilitating colonization, persistence and habitat transformation. *Biological Reviews of the Cambridge Philosophical Society*. 2018;*93*(2);1125–1144.

28. Stebbins GL, Ledyard Stebbins G. Polyploidy, hybridization, and the invasion of new habitats. Annals of the Missouri Botanical Garden. 1985;*72*(4):824–832.

29.Estep MC, McKain MR, Vela Diaz D, Zhong J, Hodge JG, Hodkinson TR, et al. Allopolyploidy,

diversification, and the Miocene grassland expansion. *Proc Natl Acad Sci US A*. 2014111:15149–15154.

30. Watson L, Macfarlane TD, Dallwitz MJ. The grass genera of the world: descriptions, illustrations, identification, and information retrieval; including synonyms, morphology, anatomy, physiology, phytochemistry, cytology, classification, pathogens, world and local distribution and references. 1992.

31. Harlan JR, de Wet JMJ. The compilospecies concept. *Evolution; International Journal of Organic Evolution*. 1963;17(4):497–501.

32. Wet JMJ, Harlan JR. *Bothriochloa intermedia* — a taxonomic dilemma. *Taxon*. 1970;19:339–340.

33. Sumadijaya A. Morphology, molecular phylogeny and genome content of *Bothriochloa* focusing on Australian taxa. Virginia Tech. 2015.

34. Putnam NH, O'Connell BL, Stites JC, Rice BJ, Blanchette M, Calef R, et al.. Chromosome-scale shotgun assembly using an in vitro method for long-range linkage. *Genome Res*. 2016; 26:342–350.

35. Chheda HR, De Wet JMJ, Harlan JR. Aneuploidy in Bothriochloa hybrids. *Caryologia*. Taylor & Francis;1961: 14:2, 205-217.

36. de Wet JMJ, Borgaonkar DS. Aneuploidy and apomixis in *Bothriochloa* and *Dichanthium*. *Bot Gaz*. The University of Chicago Press; 1963.

37. Grabherr MG, Haas BJ, Yassour M, Levin JZ, Thompson DA, Amit I, et al.. Full-length transcriptome assembly from RNA-Seq data without a reference genome. *Nature Biotechnology*. 2011; 29:644–652.

38. Cantarel BL, Korf I, Robb SMC, Parra G, Ross E, Moore B, et al.. MAKER: an easy-to-use

annotation pipeline designed for emerging model organism genomes. *Genome Res*. 2008;18:188–196

39. Stanke M, Schöffmann O, Morgenstern B, Waack S. Gene prediction in eukaryotes with a generalized hidden Markov model that uses hints from external sources. *BMC Bioinformatics*. 2006;7:62.

40. Korf I. Gene finding in novel genomes. *BMC Bioinformatics*. 2004;5:59.

41. Ou S, Su W, Liao Y, Chougule K, Agda JRA, Hellinga AJ, et al.Benchmarking transposable element annotation methods for creation of a streamlined, comprehensive pipeline. *Genome Biol*. 2019; 20(1):275.

42. Camacho C, Coulouris G, Avagyan V, Ma N, Papadopoulos J, Bealer K, et al.. BLAST+: architecture and applications. *BMC Bioinformatics*. 2009;10:421.

43. Wang Y, Tang H, Debarry JD, Tan X, Li J, Wang X, et al.. MCScanX: a toolkit for detection and evolutionary analysis of gene synteny and collinearity. *Nucleic Acids Res*. 2012;40:e49.

44. Li H. Minimap2: pairwise alignment for nucleotide sequences. *Bioinformatics*. 2018;34:3094–3100.

45. Session AM, Uno Y, Kwon T, Chapman JA, Toyoda A, Takahashi S, et al.. Genome evolution in the allotetraploid frog *Xenopus laevis*. *Nature*. 2016;538:336–343.

46. Mitros T, Session AM, James BT, Wu GA, Belaffif MB, Clark LV, et al.. Genome biology of the paleotetraploid perennial biomass crop *Miscanthus*. *Nat Commun*. 2020;11:5442.

47. Himmelmann ML: Package "HMM." https://cran.opencpu.org/web/packages/HMM/HMM.pdf (2022). Accessed 2022 Mar 29.

48. Emms DM, Kelly S. OrthoFinder: phylogenetic orthology inference for comparative genomics. *Genome Biol*. 2019; 20:238.

49. Alexa A, Rahnenfuhrer J. TopGO: enrichment analysis for gene ontology. R package version. 2006.

50. Edger PP, Poorten TJ, VanBuren R, Hardigan MA, Colle M, McKain MR, et al..Origin and evolution of the octoploid strawberry genome. *Nat Genet*. 2019;51(3):541-547.

51. Hirsch CD, Springer NM. Transposable element influences on gene expression in plants. *Biochim Biophys Acta Gene Regul Mech*. 2017;1860:157–165.

52. Stroud H, Greenberg MVC, Feng S, Bernatavichute YV, Jacobsen SE. Comprehensive analysis of silencing mutants reveals complex regulation of the *Arabidopsis* methylome. *Cell*. 2013;152:352–364.

53.Sankoff D, Zheng C, Zhu Q. The collapse of gene complement following whole genome duplication. *BMC Genomics*. 2010;11:313.

54. Schnable PS, Ware D, Fulton RS, Stein JC, Wei F, Pasternak S, et al.. The B73 maize genome: complexity, diversity, and dynamics. *Science*. 2009;*326*(5956):1112–1115.

55. Griffiths AG, Moraga R, Tausen M, Gupta V, Bilton TP, Campbell MA, et al.. Breaking free: The genomics of allopolyploidy-facilitated niche expansion in white clover. *Plant Cell*. 2019;31:1466–1487.

56. Cheng F, Sun C, Wu J, Schnable J, Woodhouse MR, Liang J, et al.. Epigenetic regulation of subgenome dominance following whole genome triplication in *Brassica rapa*. *New Phytol*. 2016;211(1):288-99.

57. Schnable JC, Freeling M. Genes identified by visible mutant phenotypes show increased bias toward one of two subgenomes of *maize*. *PLoS One*. 2011;6(3):e17855. doi: 10.1371/journal.pone.0017855. PMID: 21423772; PMCID: PMC3053395.

58. Weber M, Hellmann I, Stadler MB, Ramos L, Pääbo S, Rebhan M, et al.. Distribution, silencing potential and evolutionary impact of promoter DNA methylation in the human genome. *Nat Genet*. 2007; 39:457–466.

59. Martienssen RA, Colot V. DNA methylation and epigenetic inheritance in plants and filamentous fungi. *Science*. 2001; 293:1070–1074.

60. Blanc G, Wolfe KH. Functional divergence of duplicated genes formed by polyploidy during *Arabidopsis* evolution. *Plant Cell*. 2004;16:1679–1691.

61. Duarte JM, Wall PK, Edger PP, Landherr LL, Ma H, Pires JC, et al.. Identification of shared single copy nuclear genes in *Arabidopsis*, *Populus*, *Vitis* and *Oryza* and their phylogenetic utility across various taxonomic levels. *BMC Evol Biol*. 2010;10:61.

62. Maere S, De Bodt S, Raes J, Casneuf T, Van Montagu M, Kuiper M, et al.. Modeling gene and genome duplications in eukaryotes. *Proc Natl Acad Sci U S A*. 2005; 102:5454–5459.

63. Paterson AH, Chapman BA, Kissinger JC, Bowers JE, Feltus FA, Estill JC. Many gene and domain families have convergent fates following independent whole-genome duplication events in *Arabidopsis*, *Oryza*, *Saccharomyces* and *Tetraodon*. *Trends Genet*. 2006;22:597– 602.

64.Seoighe C, Gehring C. Genome duplication led to highly selective expansion of the *Arabidopsis thaliana* proteome. *Trends Genet*. 2004;20:461–464.

65. Haig D. From Darwin to Derrida: Selfish genes, social selves, and the meanings of life. MIT Press.2020.

66. Kleine T, Voigt C, Leister D. Plastid signalling to the nucleus: messengers still lost in the mists? *Trends Genet*. 2009; 25:185–192.

67. Greiner S, Rauwolf U, Meurer J, Herrmann RG. The role of plastids in plant speciation. *Mol*

Ecol. 2011;20(4):671-91.

68. Emery M, Willis MMS, Hao Y, Barry K, Oakgrove K, Peng Y, et al.. Preferential retention of genes from one parental genome after polyploidy illustrates the nature and scope of the genomic conflicts induced by hybridization. *PLoS Genet*. 2018;14:e1007267.

69. Wu S, Han B, Jiao Y. Genetic contribution of paleopolyploidy to adaptive evolution in angiosperms. *Mol Plant*. 2020;13:59–71.

70. McGrath CL, Lynch M. Evolutionary significance of whole-genome duplication.*Polyploidy and genome evolution*. 2012 (Vol. 9783642314421, pp. 1-20).

71. Ohno S. Evolution by Gene Duplication. 1970.

72. Cheng F, Wu J, Cai X, Liang J, Freeling M, Wang X. Gene retention, fractionation and subgenome differences in polyploid plants. *Nat Plants*. 2019;4:258–268.

73. Defoort J, Van de Peer Y, Carretero-Paulet L. The Evolution of gene duplicates in angiosperms and the impact of protein–protein interactions and the mechanism of duplication. *Genome Biol Evol*. Oxford Academic. 2019;11:2292–2305.

74. Jiao H, Wang Y, Zhang L, Jiang P, Zhao H. Lineage-specific duplication and adaptive evolution of bitter taste receptor genes in bats. *Mol Ecol*. 2018; 27:4475–4488.

75. Panchy N, Lehti-Shiu M, Shiu S-H. Evolution of gene duplication in plants. *Plant Physiol*. 2016;171:2294–2316.

76. Zhang L, Wu S, Chang X, Wang X, Zhao Y, Xia Y, et al.The ancient wave of polyploidization events in flowering plants and their facilitated adaptation to environmental stress. *Plant Cell Environ*. 2020; 43:2847–2856.

77. Edwards EJ, Osborne CP, Strömberg CAE, Smith SA, C4 Grasses Consortium, Bond WJ, et al.The origins of C4 grasslands: integrating evolutionary and ecosystem science. *Science*. 2010;328:587–591.

78. Wicker T, Keller B. Genome-wide comparative analysis of copia retrotransposons in Triticeae, rice, and *Arabidopsis* reveals conserved ancient evolutionary lineages and distinct dynamics of individual copia families. *Genome Res*. 2007;17:1072–1081.

79. De Wet JMJ, Harlan JR. Morphology of the compilospecies *Bothriochloa intermedia. Am J Bot*. Wiley; 1966;53:94-98.

80. Godfree RC, Marshall DJ, Young AG, Miller CH, Mathews S. Empirical evidence of fixed and homeostatic patterns of polyploid advantage in a keystone grass exposed to drought and heat stress. *R Soc Open Sci*. 2017; 4:170934.

81. Linder HP, Barker NP. Does polyploidy facilitate long-distance dispersal? *Ann Bot*. 2014;113:1175–1183.

82. Breed MF, Stead MG, Ottewell KM, Gardner MG, Lowe AJ. Which provenance and where? Seed sourcing strategies for revegetation in a changing environment. *Conserv Genet*.2013; 14:1–10.

83. Aitken SN, Whitlock MC. Assisted gene flow to facilitate local adaptation to climate change. *Annu Rev Ecol Evol Syst*. Annual Reviews.2013; 44:367–388.

84. Stanley TD, Ross EM. Flora of south-eastern Queensland. Queensland Dept. of Primary Industries.1983.

85. Simon BK, Alfonso Y. AusGrass2. Brisbane, Queensland, Australia. 2011.

86. De Wet JMJ, Higgins ML. Species relationships within the *Bothriochloa pertusa* complex. *Phyton, Vicente Lopez*. 1963. 20, 205-11.

87. Dolezel J, Greilhuber J, Suda J. Estimation of nuclear DNA content in plants using flow cytometry. *Nat Protoc*. 2007;2:2233–2244.

88. Galbraith DW, Lambert GM. High-throughput monitoring of plant nuclear DNA contents via flow cytometry. *Methods Mol Biol*. 2012; 918:311–325.

89. Lieberman-Aiden E, van Berkum NL, Williams L, Imakaev M, Ragoczy T, Telling A, et al.. Comprehensive mapping of long-range interactions reveals folding principles of the human genome. *Science*. 2009; 326:289–293.

90. Weisenfeld NI, Kumar V, Shah P, Church DM, Jaffe DB. Direct determination of diploid genome sequences. *Genome Res*. 2017;27:757–767.

91. Zaharia M, Bolosky WJ, Curtis K, Fox A, Patterson D, Shenker S, et al.. Faster and more accurate sequence alignment with SNAP. 2011. arXiv. http://arxiv.org/abs/1111.5572.

92. Robinson JT, Turner D, Durand NC, Thorvaldsdóttir H, Mesirov JP, Aiden EL. Juicebox.js provides a cloud-based visualization system for Hi-C Data. *Cell Syst*. 2018;*6*(2):256-258.

93. Bolger AM, Lohse M, Usadel B. Trimmomatic: a flexible trimmer for Illumina sequence data. *Bioinformatics*. 2014;30:2114–2120.

94. Campbell MS, Law M, Holt C, Stein JC, Moghe GD, Hufnagel DE, et al.. MAKER-P: a tool kit for the rapid creation, management, and quality control of plant genome annotations. *Plant Physiol*. 2014;164:513–524.

95. Han Y, Wessler SR. MITE-Hunter: a program for discovering miniature inverted-repeat transposable elements from genomic sequences. *Nucleic Acids Res*. 2010;38:e199.

96. Ellinghaus D, Kurtz S, Willhoeft U. LTRharvest, an efficient and flexible software for de novo detection of LTR retrotransposons. *BMC Bioinformatics*. 2008; 9:18.

97. Steinbiss S, Willhoeft U, Gremme G, Kurtz S. Fine-grained annotation and classification of de novo predicted LTR retrotransposons. *Nucleic Acids Res*. 2009;37:7002–7013.

98. Smit AFA, Hubley R. RepeatModeler (v.2.0.3). 2015. http://www.repeatmasker.org. Accessed 10 November 2020.

99.Plant Protein Database. http://www.hrt.msu.edu/uploads/535/78637/alluniRefprexp070416.gz. Accessed 20 January 2020.

100. All Transposase Protein Database. http://www.hrt.msu.edu/uploads/535/78637/Tpases020812.gz. Accessed 2 February 2020.

101. Smit AFA, Hubley R, Green P. *RepeatMasker Open-4.0*. http://repeatmasker.org. 2015.

102. Kennedy RC, Unger MF, Christley S, Collins FH, Madey GR. An automated homology based approach for identifying transposable elements. *BMC Bioinformatics*. 2011;12:130.

103. Jiang N, Bao Z, Zhang X, Hirochika H, Eddy SR, McCouch SR, et al.. An active DNA transposon family in rice. *Nature*. 2003;421:163–167.

104. Altschul SF, Gish W, Miller W, Myers EW, Lipman DJ. Basic local alignment search tool. *J Mol Biol*. 1990; 215:403–410.

105. Storer J, Hubley R, Rosen J, Wheeler TJ, Smit AF. The Dfam community resource of transposable element families, sequence models, and genome annotations. *Mob DNA*.2021; 12:2.

106. Eddy SR. A new generation of homology search tools based on probabilistic inference. *Genome Inform*. 2009; 23:205–211.

107. Dfamscan.pl. https://dfam.org/releases/current/infrastructure/dfamscan.pl.gz. Accessed 11 March 2020.

108. Seppey M, Manni M, Zdobnov EM. BUSCO: Assessing genome assembly and annotation 1035 completeness. *Methods Mol Biol*. 2019;1962:227–245.

109. Genestats. https://github.com/darencard/GenomeAnnotation/blob/97fa52d13eb7a8f6b59a0f6e7261e1e08e542126/genestats. Accessed 14 May 2021.

110.Manni M, Berkeley MR, Seppey M, Simão FA, Zdobnov EM. BUSCO update: novel and streamlined workflows along with broader and deeper phylogenetic coverage for scoring of eukaryotic, prokaryotic, and viral genomes. *Mol Biol Evol*. 2021; 38:4647–4654.

111. Winter D, Lee K, Cox M. Pafr: Read, manipulate and visualize “pairwise mapping ormat” Data. R package version 0.0.2. 2020.

112.Marçais G, Kingsford C. A fast, lock-free approach for efficient parallel counting of occurrences of k-mers. *Bioinformatics*. 2011;27:764–770.

113. Gu Z, Eils R, Schlesner M. Complex heatmaps reveal patterns and correlations in multidimensional genomic data. *Bioinformatics*. 2016;32:2847–2849.

114. van Dongen S, Abreu-Goodger C. Using MCL to extract clusters from networks. *Methods Mol Biol*. 2012;804:281–295.

115. UniProt Consortium. UniProt: the universal protein knowledgebase in 2021. *Nucleic Acids Res*. 2021; 49(D1):D480–D489.

116. Berardini TZ, Reiser L, Li D, Mezheritsky Y, Muller R, Strait E, et al.. The *Arabidopsis* information resource: Making and mining the “gold standard” annotated reference plant genome.

Genesis. 2015;53:474–485.

117. Quevillon E, Silventoinen V, Pillai S, Harte N, Mulder N, Apweiler R, et al.. InterProScan: protein domains identifier. *Nucleic Acids Res*. 2005; *33*(Web Server issue):W116–W120.

118. Punta M, Coggill PC, Eberhardt RY, Mistry J, Tate J, Boursnell C, et al.. The Pfam protein families database. *Nucleic Acids Res*. 2012; 40:D290–D301.

119. Kanehisa M. Toward understanding the origin and evolution of cellular organisms. *Protein Sci*. 2019; 28:1947–1951.

120. Kanehisa M, Goto S. KEGG: kyoto encyclopedia of genes and genomes. *Nucleic Acids Res*. 2000;28:27–30.

121. Kanehisa M, Furumichi M, Sato Y, Ishiguro-Watanabe M, Tanabe M. KEGG: integrating viruses and cellular organisms. *Nucleic Acids Res*. 2021; 49:D545–D551.

122. Aramaki T, Blanc-Mathieu R, Endo H, Ohkubo K, Kanehisa M, Goto S, et al.. KofamKOALA: KEGG Ortholog assignment based on profile HMM and adaptive score threshold. *Bioinformatics*. 2020;36:2251–2252.

123. Bandi V, Gutwin C. Interactive exploration of genomic conservation. 2020. https://openreview.net/pdf?id=7-C5VJWbnI.

124. Goodstein DM, Shu S, Howson R, Neupane R, Hayes RD, Fazo J, et al.. Phytozome: a comparative platform for green plant genomics. *Nucleic Acids Res*. 2012;40:D1178–1186.

125. Zhang J, Zhang X, Tang H, Zhang Q, Hua X, Ma X, et al.. Allele-defined genome of the autopolyploid sugarcane *Saccharum spontaneum* L. *Nat Genet*. 2018;50:1565–1573.

126.Mao Y. GenoDup Pipeline: a tool to detect genome duplication using the dS-based method.

PeerJ. 2019;7:e6303.

127.Katoh K, Standley DM. MAFFT multiple sequence alignment software version 7:improvements in performance and usability. *Mol Biol Evol*. 2013;30:772–780.

128. Talavera G, Castresana J. Improvement of phylogenies after removing divergent and ambiguously aligned blocks from protein sequence alignments. *Syst Biol*. 2007;56:564–577.

129. Darriba D, Taboada GL, Doallo R, Posada D. jModelTest 2: more models, new heuristics and parallel computing. *Nat Methods*. 2012; 9:772.

130. Guindon S, Gascuel O. A simple, fast, and accurate algorithm to estimate large phylogenies by maximum likelihood. *Syst Biol*. 2003;52:696–704.

131. Stamatakis A. RAxML version 8: a tool for phylogenetic analysis and post-analysis of large phylogenies. *Bioinformatics*. 2014; 30:1312–1313.

132. Bouckaert R, Vaughan TG, Barido-Sottani J, Duchêne S, Fourment M, Gavryushkina A, et al.. BEAST 2.5: An advanced software platform for Bayesian evolutionary analysis. *PLoS Comput Biol*. 2019;15:e1006650.

133. Suchard MA, Lemey P, Baele G, Ayres DL, Drummond AJ, Rambaut A. Bayesian phylogenetic and phylodynamic data integration using BEAST 1.10. *Virus Evol*. 2018; 4:vey016.

134. Bouckaert RR, Drummond AJ. bModelTest: Bayesian phylogenetic site model averaging and model comparison. *BMC Evol Biol*. 2017;17:42.

135. Kumar S, Stecher G, Suleski M, Hedges SB. TimeTree: A resource for timelines, timetrees, and divergence times. *Mol Biol Evol*. 2017; 34:1812–1819.

136.Rambaut A, Drummond AJ, Xie D, Baele G, Suchard MA. Posterior summarization in Bayesian

phylogenetics using Tracer 1.7. *Syst Biol*. 2018; 67:901–904.

137. Paradis E, Schliep K. Ape 5.0: An environment for modern phylogenetics and evolutionary analyses in R. *Bioinformatics*. Oxford University Press (OUP). 2019;35:526– 528.

138. Moniz de Sá M, Drouin G. Phylogeny and substitution rates of angiosperm actin genes. *Mol Biol Evol*. 1996;13:1198–1212.

139. Ma J, Bennetzen JL. Rapid recent growth and divergence of rice nuclear genomes. *Proc Natl Acad Sci U S A*. 2004;101:12404–12410.

140. Teresi SJ, Teresi MB, Edger PP. TE Density: a tool to investigate the biology of transposable elements. *Mob DNA*. 2022;*13*(1), 1-18.

141. R scripts used in this manuscript. https://github.com/NissankaPD/B_decipiens_genome.

142. De Silva NP, Lee C, Battlay P, Fournier-Level A, Moore JL, Hodgins KA. Supporting data for "Genome assembly of an Australian native grass species reveals a recent whole genome duplication and biased gene retention of genes involved in stress response" GigaScience Database. 2023. http://dx.doi.org/10.5524/102378

Figure

Click here to access/download;Figure;Figure_1.png

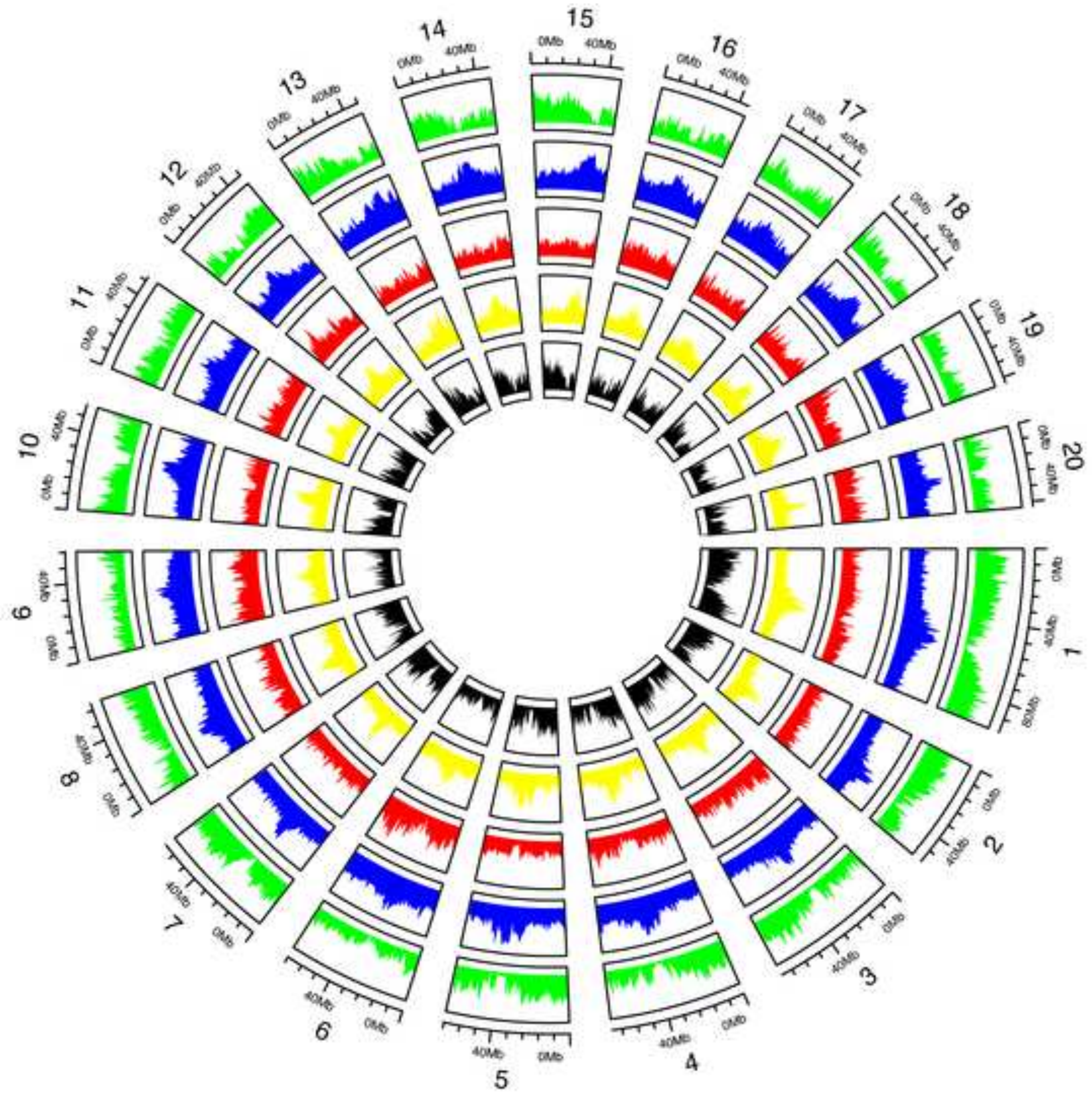

Figure Click here to access/download;Figure;Figure_2.png

A

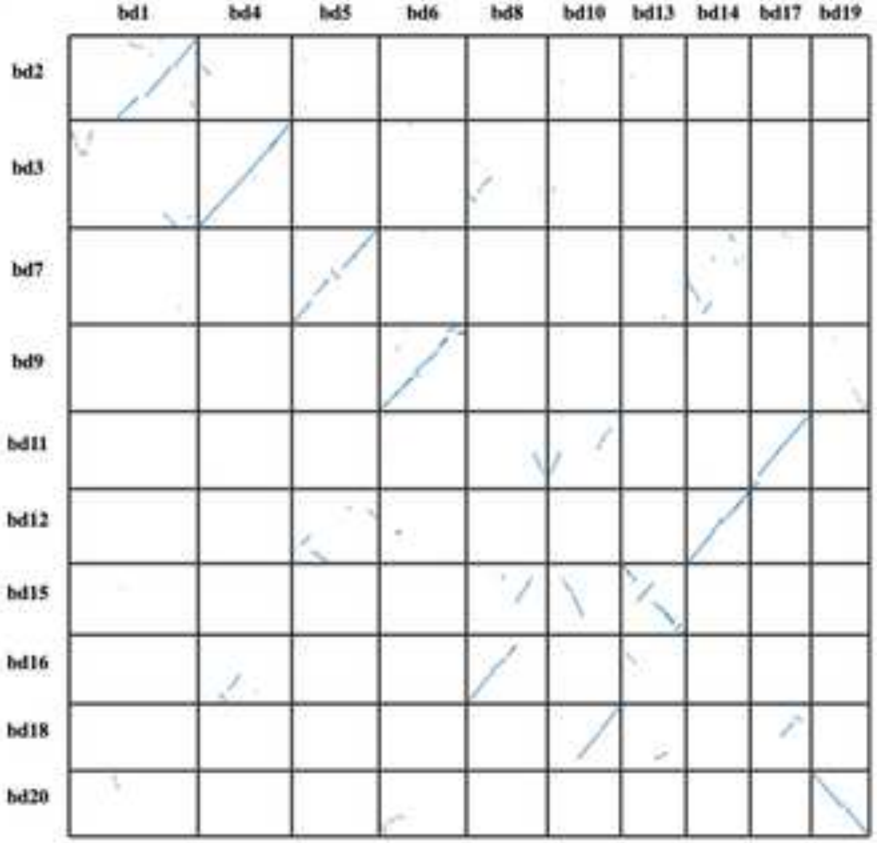

B

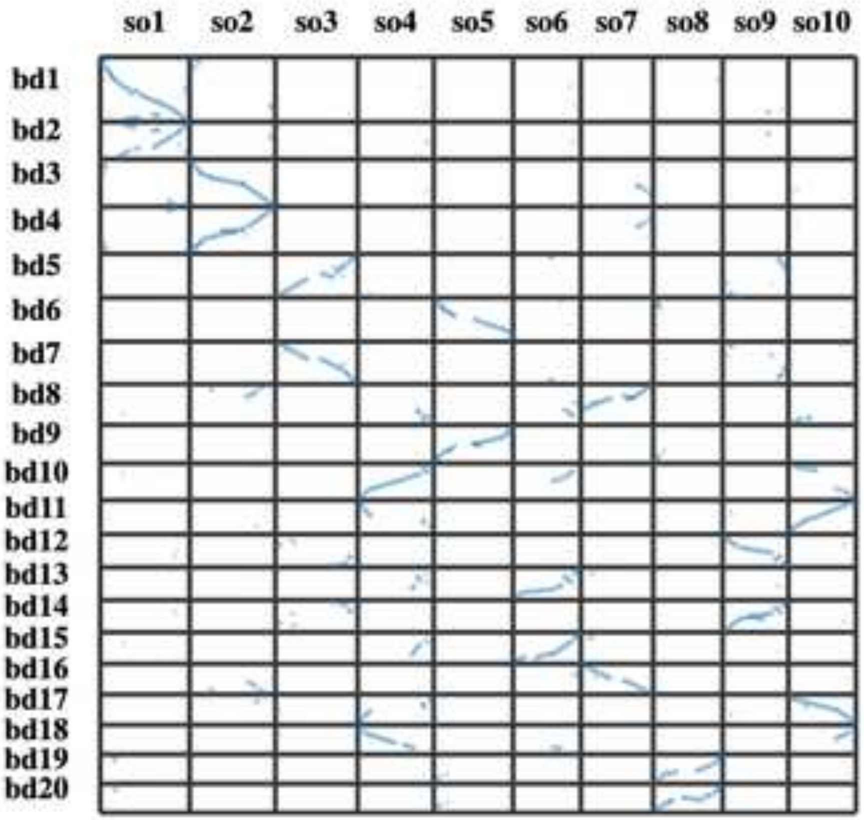

Figure

Click here to access/download;Figure;Figure_3.png

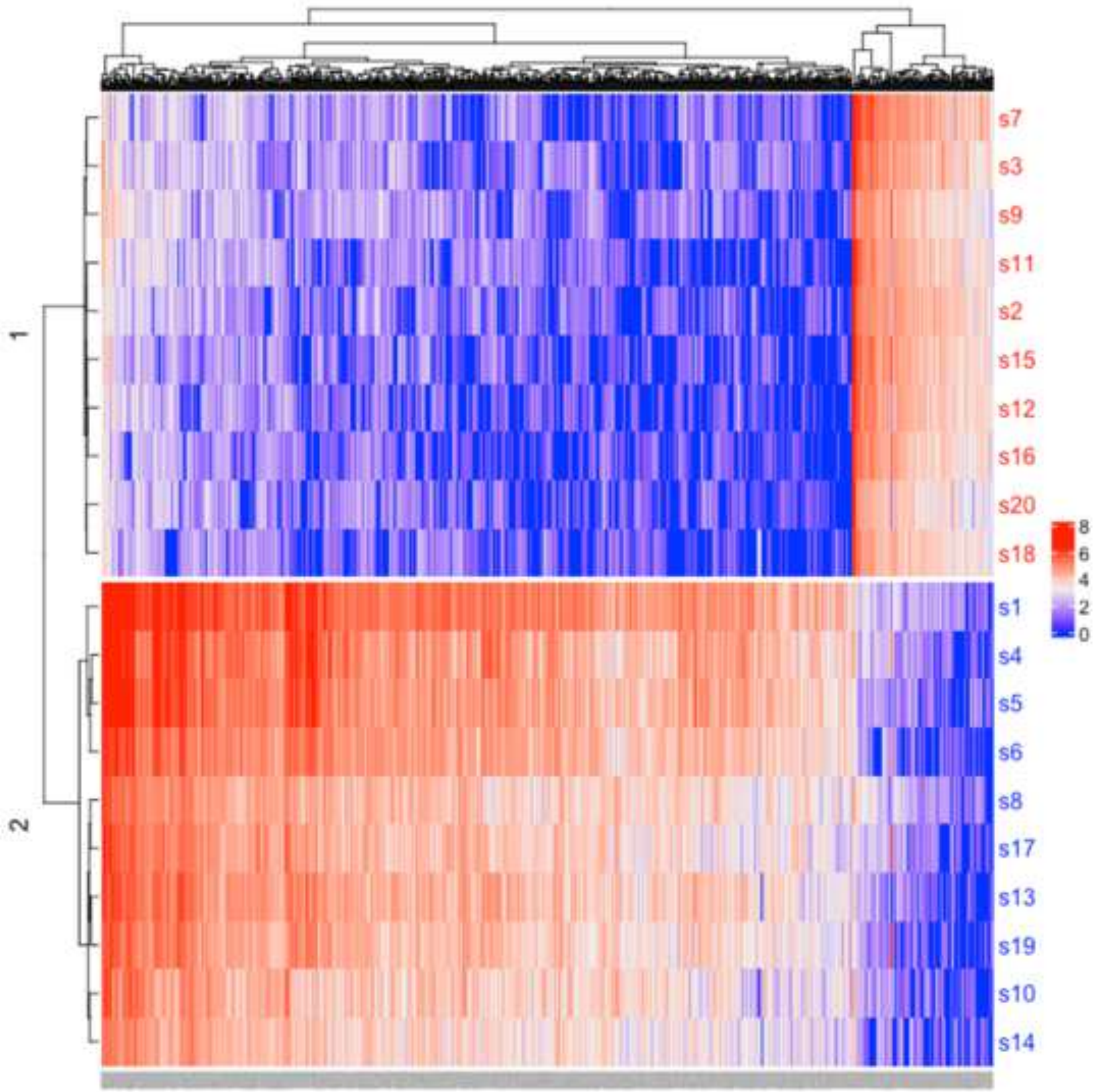

Figure Click here to access/download;Figure;Figure_4.png

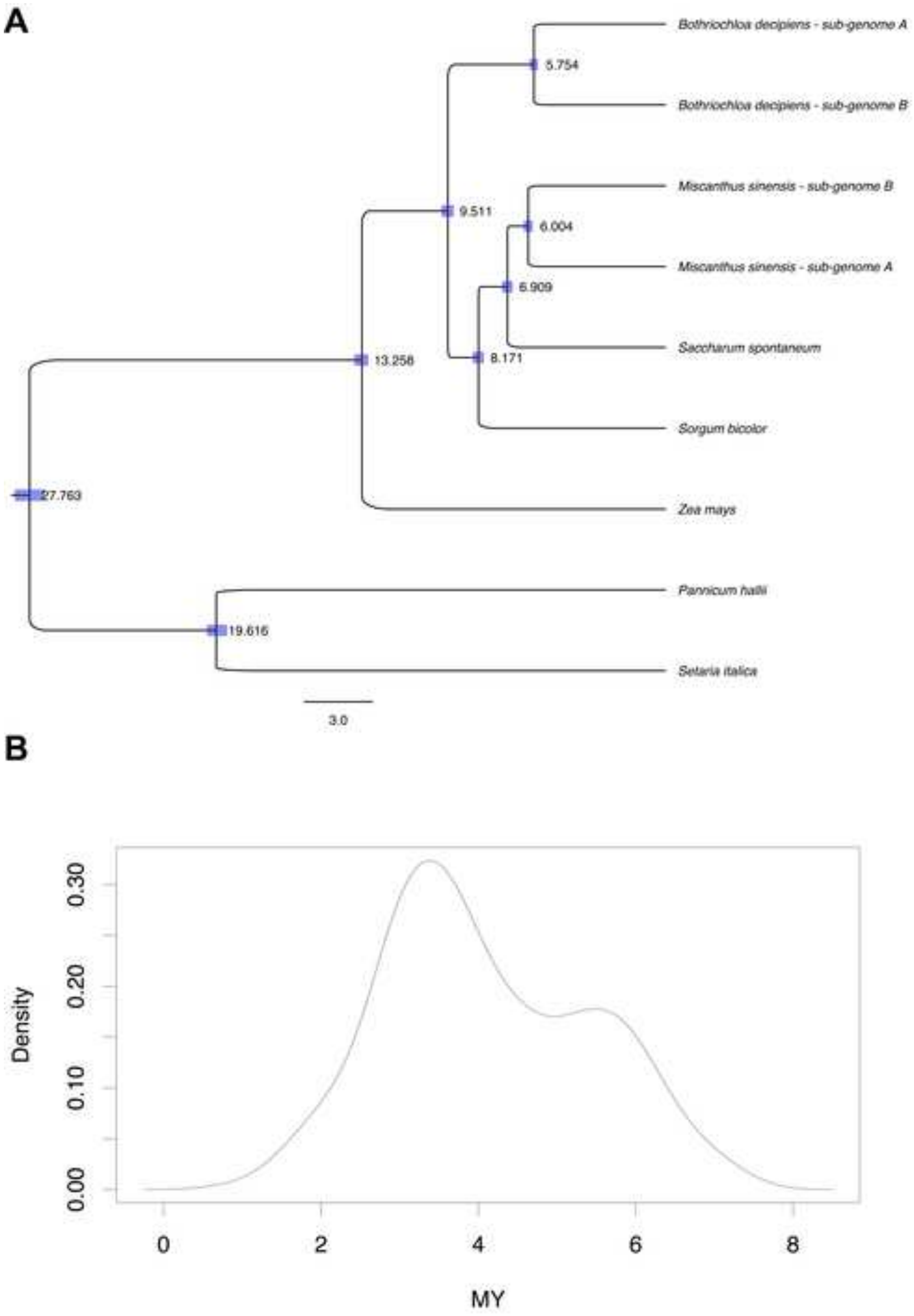

Click here to access/download
Supplementary Material
Supplementary_Figure_captions_and_tables.docx

Click here to access/download
Supplementary Material
Supplementary_Figure_S1.png

Click here to access/download
Supplementary Material
Supplementary_figure_S2.png

Click here to access/download
Supplementary Material
Supplementary_Figure_S3.png

Click here to access/download
Supplementary Material
Supplementary_figure_S4.png

Click here to access/download
Supplementary Material
Supplementary_Figure_S5.png

Click here to access/download
Supplementary Material
Supplementary_Figure_S6.png

Click here to access/download
Supplementary Material
Supplementary_Figure_S7.png

Click here to access/download
Supplementary Material
Supplementary_Figure_S8.png
