# Supplementary material for: Genome assembly of an Australian native grass species reveals a recent whole-genome duplication and biased gene retention of genes involved in stress response
Source: Gigascience. 2023 May 12;12:giad034. doi: 10.1093/gigascience/giad034 (PMC10176504; doi:10.1093/gigascience/giad034)
Supplement: giad034_Supplemental_Files [file giad034_supplemental_files.zip › Supplementary_Figure_captions_and_tables.docx]

**Supplementary material**

Figure S1 Mapping of the *Bothriochloa decipiens* genome against itself to identify putative homeologous chromosomes

Figure S2 The dot plot depicts the syntenic relationship of the 20 chromosomes of  *B. decipiens* (bd) aligned against themselves.

Figure S3 A) Density of sub genome A preferred kmers. B) Density of sub genome B preferred kmers. C) Repeats marked by overrepresented kmers in sub genome A. D) Repeats marked by overrepresented kmers in sub genome B. The scaffolds belonging to putative subgenomes (Figure 3) are paired together as homeologs one after another. These likely reflect repeat expansions during the period the species involved in the paleopolyploid event were separated.

Figure S4. Top 30 GO terms over-represented in A) genes retained as single-copy and B) genes retained as duplicates following the paleo tetraploidy event.

Figure S5. Indicative flow cytometry results of *Solanum* and *Pisum* size standards, and 2C genome sizes of diploid *Bothriochloa decipiens* and tetraploid *Bothriochloa macra*. *(a)* *Solanum*; FL2 mean = 182,944. *(b) B. decipiens*; FL2 mean = 249,158. (*c*) *Pisum*; FL2 mean = 777095. (*d*) *B. macra*; FL2 mean =480804. Smaller, additional peaks represent the smaller proportion of cells in G2 phase of cell division.

Figure S6. Estimated genome size for samples from all populations, and two internal standards (*Pisum sativum* and *Solanum lycopersicum*).

Figure S7. Misjoins in two scaffolds of the first assembly before the manual edits are shown in black circles.

Figure S8. Scaffold Hi-C contact map. The x and y axes give the mapping positions of the first and second read in the read pair respectively, grouped into bins. The colour of each square gives the number of read pairs within that bin. White vertical and black horizontal lines have been added to show the borders between scaffolds. Scaffolds less than 1 Mb are excluded

Table S1. Summary of the annotation for protein coding genes.

| Primary transcripts (i.e., longest at each locus) | 60,652 |
| --- | --- |
| Average exon number/gene | 5.46 |
| Median transcript length (bp) | 2880 |
| Median CDS length (bp) | 1017 |
| Median exon length (bp) | 269 |
| Median intron length (bp) | 359 |
| Median 3’ UTR length (bp) | 292 |
| Median 5' UTR length (bp) | 135 |

Table S2. Illumina shotgun libraries for *Bothriochloa decipiens*.

| Library Type | Bases (billions) | Read pairs (millions) | Read length |
| --- | --- | --- | --- |
| 10X | 120 | 400 million | 2x150bp |
| Chicago | 140 | 460 million | 2x150bp |
| HiC | 120 | 400 million | 2x150bp |
