# Supplementary figures and images for: Genome assembly of an Australian native grass species reveals a recent whole-genome duplication and biased gene retention of genes involved in stress response
Source: Gigascience. 2023 May 12;12:giad034. doi: 10.1093/gigascience/giad034 (PMC10176504; doi:10.1093/gigascience/giad034)

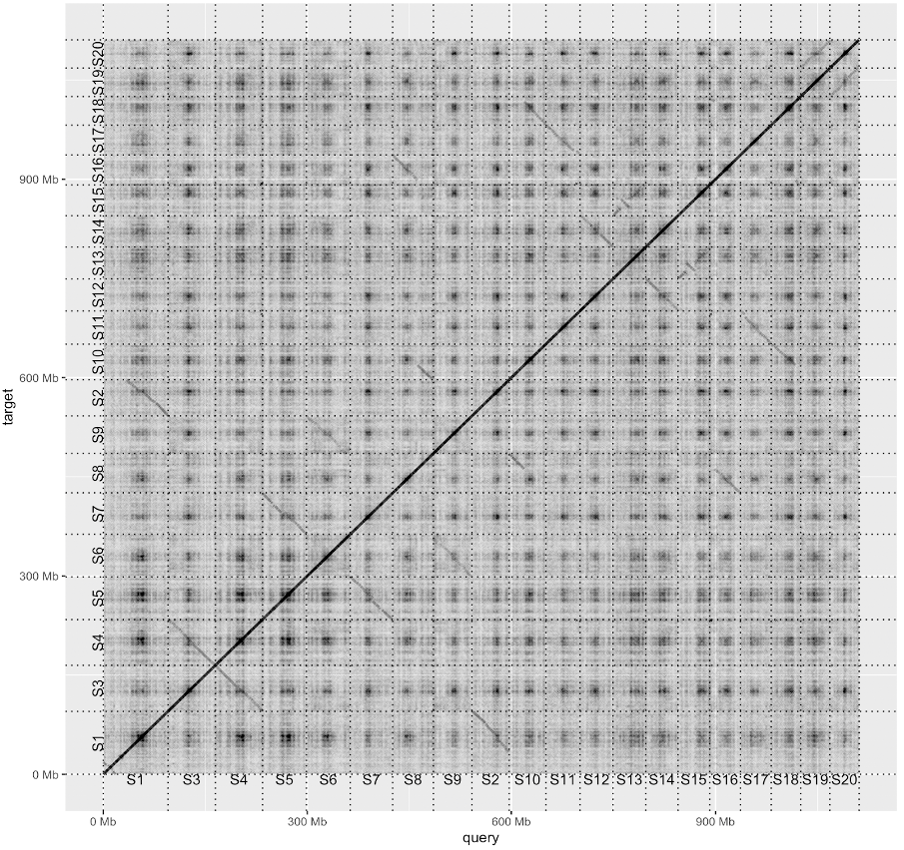

Supplement: giad034_Supplemental_Files [file giad034_supplemental_files.zip › Supplementary_Figure_S1.png]

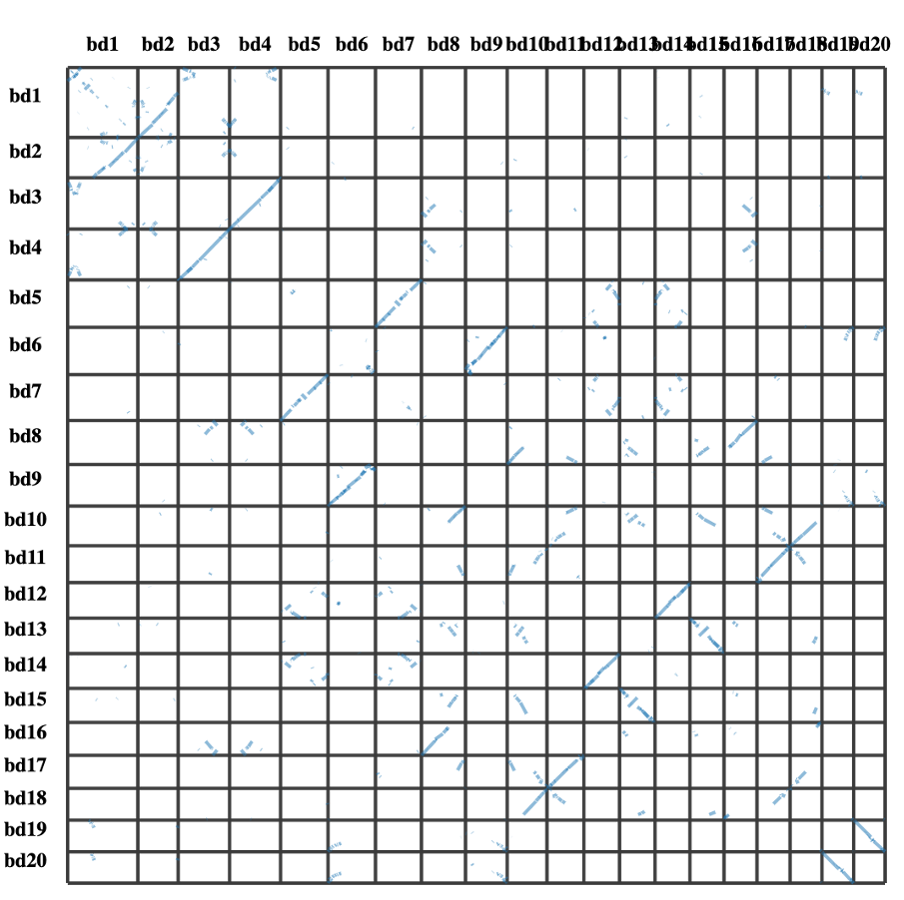

Supplement: giad034_Supplemental_Files [file giad034_supplemental_files.zip › Supplementary_figure_S2.png]

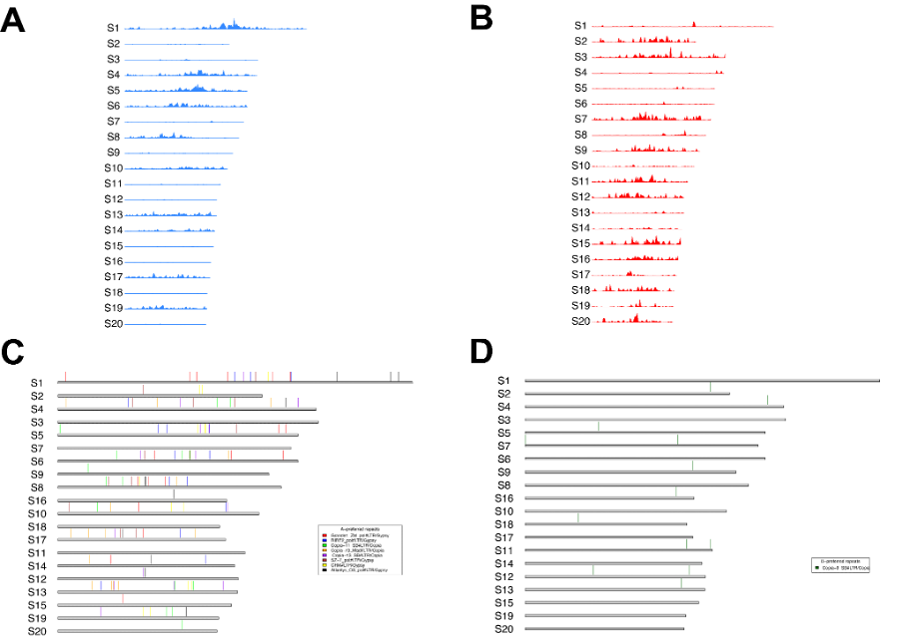

Supplement: giad034_Supplemental_Files [file giad034_supplemental_files.zip › Supplementary_Figure_S3.png]

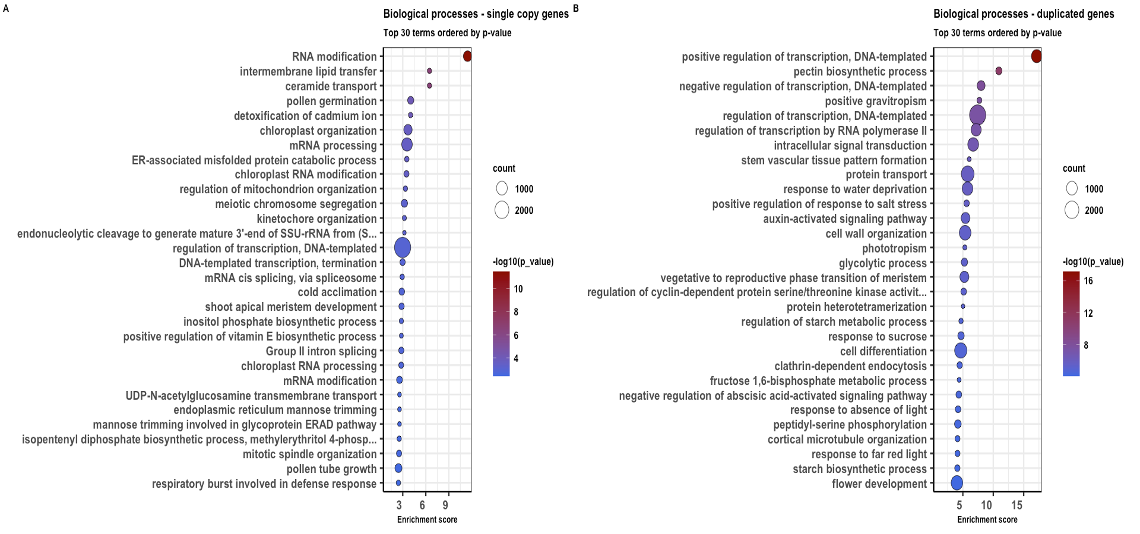

Supplement: giad034_Supplemental_Files [file giad034_supplemental_files.zip › Supplementary_figure_S4.png]

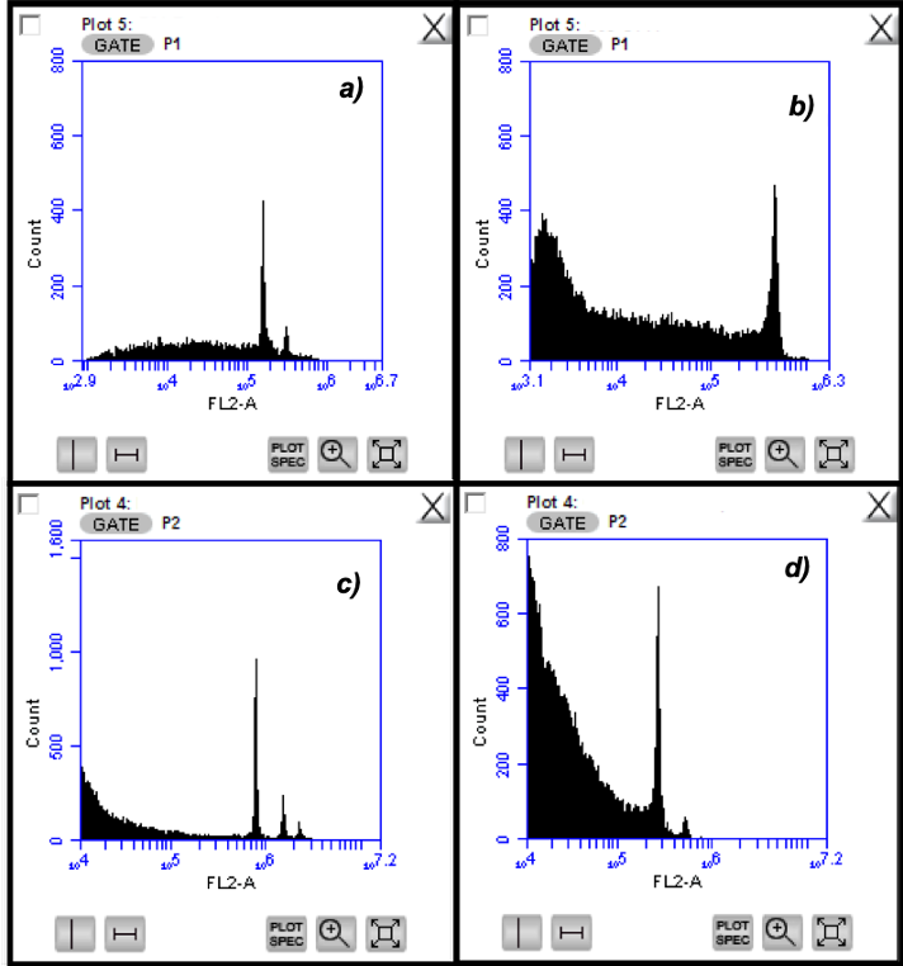

Supplement: giad034_Supplemental_Files [file giad034_supplemental_files.zip › Supplementary_Figure_S5.png]

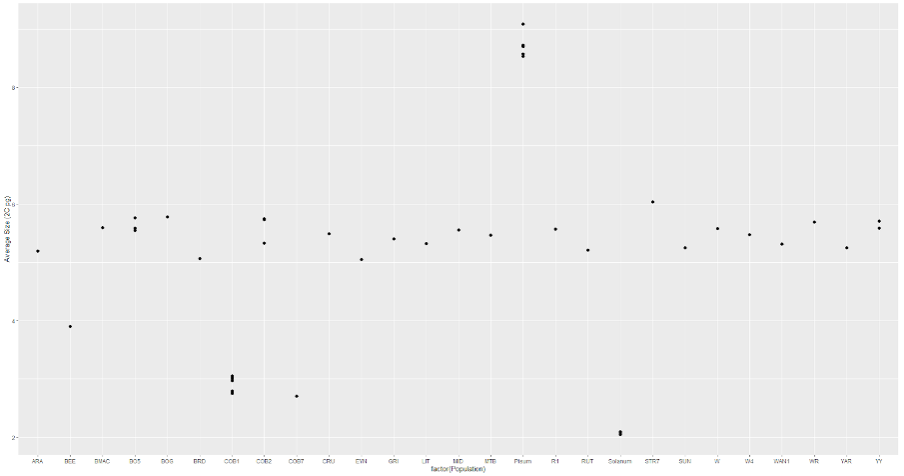

Supplement: giad034_Supplemental_Files [file giad034_supplemental_files.zip › Supplementary_Figure_S6.png]

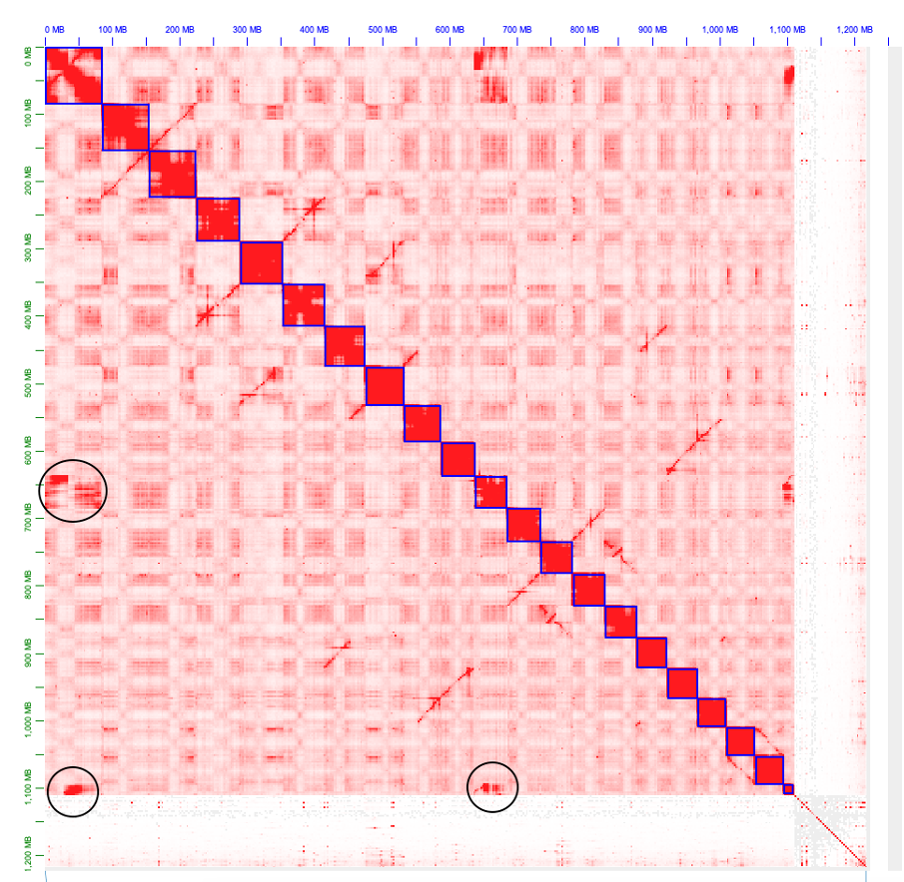

Supplement: giad034_Supplemental_Files [file giad034_supplemental_files.zip › Supplementary_Figure_S7.png]

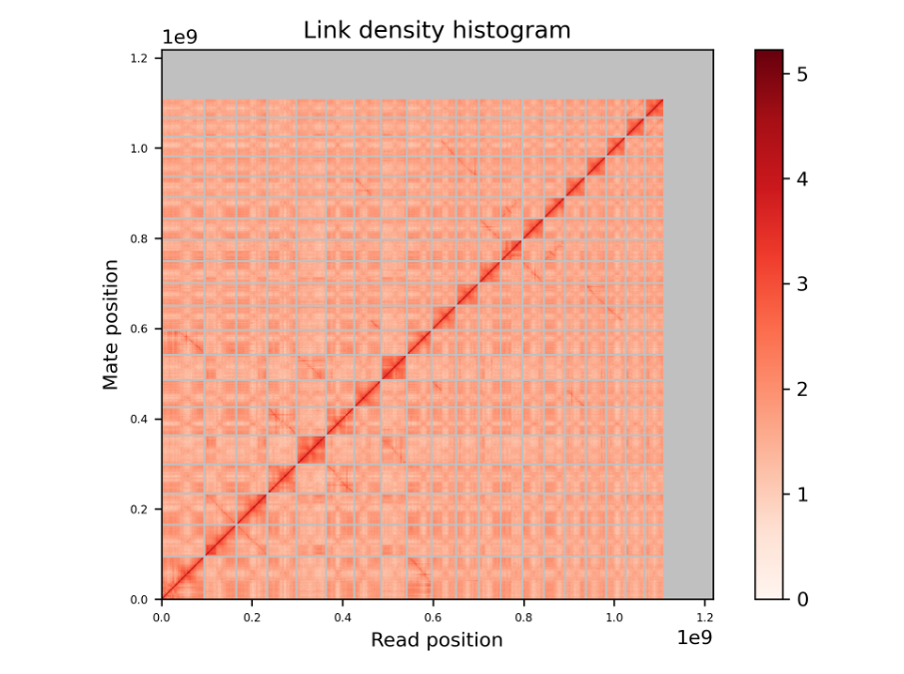

Supplement: giad034_Supplemental_Files [file giad034_supplemental_files.zip › Supplementary_Figure_S8.png]
