# Supplementary material for: Genome assembly of an Australian native grass species reveals a recent whole-genome duplication and biased gene retention of genes involved in stress response
Source: Gigascience. 2023 May 12;12:giad034. doi: 10.1093/gigascience/giad034 (PMC10176504; doi:10.1093/gigascience/giad034)
Supplement: giad034_Response_to_Reviewer_Comments_Original_Submission [file giad034_response_to_reviewer_comments_original_submission.pdf]

Author's Response To Reviewer Comments

Close

REVIEWER COMMENTS

Reviewer #1: (Remarks to the Author):

This manuscript describes the genome sequencing project of Bothriochloa decipiens, an Australian grass from BCD clade. Using the newly sequenced genome of B. decipiens, the authors have found evidence of biased fractionation and differences in LTR-retrotransposons activity between the sub genomes prior to hybridization. The method used to organise the sub-genomes using repeat signatures is interesting. Also, future work with more in-depth analysis revealing the relationship between the active LTRs and high number of retained genes in sub-genome A would be really informative. Overall, as BCD clade represents species with a complex history of hybridization and polyploidy, the availability of chromosomal level genome of Bothriochloa decipiens would facilitate understanding evolutionary studies at various levels. Hence, I accept the manuscript for publication.

The placement of figure legends in between the main document texts (Pg 8:153-156, 10:187-191, 11:232-234, 12:259-262, 15:295-296) is hard to follow. Please keep all figure legends in one page.
We have now removed the figure legends in between the main text document and added all figure legends to page 40 (line 794-810).

Reviewer #2: (Remarks to the Author):

De Silva et al. present a chromosome-scaffolded genome of Bothriochloa decipiens. The manuscript is primarily a genome report, focusing on gene content and pattern of gene retention post whole genome duplication. There are a few areas that the authors should consider in a revision to improve the genome annotation and subsequent analyses.

The transposon annotation and description could use more detail on the superfamilies that were identified. More diversity of superfamilies, especially those considered DNA elements, should be described. The methods for annotating the elements focused on LTRs and MITEs. There is no indication of annotation for elements like helitrons. I would recommend using software like EDTA since it covers the majority of TE types.
We thank the reviewer for this suggestion to expand our repeat annotation to include more details on the repeat family diversity present in the genome. We have now used the EDTA pipeline as suggested by the reviewer to further annotate the repeats and have included a table depicting the major repeat classes and the superfamilies present within these major classes along with the percentage of the genome covered by each superfamily (Table 3, page 9). Further we have included a new section in our methods "Detailed identification of repeat family diversity in the genome" to describe the use of the EDTA pipeline for the repeat annotations page 31 (lines 592-598).

The finding that the more dominant subgenome also had more TE activity is interesting. It would be useful for the authors to describe the location of TEs relative to gene space. Other studies have demonstrated that there is a link between TE density near genes and paralog loss post WGD.
We thank the reviewer for this interesting suggestion. In order to address this suggestion we used a pipeline name "TE density" (Teresi et al., 2022) that calculates TE density defined as the number of TE-occupied base pairs in a given window divided by the window value relative to the genes in the genome. We calculated the TE densities using this for all the genes present in the genome as well as genes identified as single copy and duplicate copy as identified previously by us in our analyses to identify biases in gene

retention between the subgenomes. Using these calculated average TE densities with relation to gene space, we used Mann-Whitney U tests to assess if the average TE density values were significantly different between the two subgenomes and also between genes identified as single copy or duplicated. We did not find any evidence of significant difference in TE density values in relation to gene space between the two subgenomes or between the genes retained as single copy or as duplicates. This new analysis is reported in the manuscript in page 17 lines 263-283. The methods followed for this new addition is written in page 37 (lines 727-741).

Check the spelling of "Andropogoneae" and "homoeologous" throughout.
We thank the reviewer for pointing out this oversight. We have now checked each time the word “Andropogoneae” appears in the manuscript and corrected any spelling errors We have now checked each time the word “homoeologous” appears in the manuscript and corrected any spelling errors.

Figure 1: I would create a key that uses the track colours to assign the letters. As they are placed, they are difficult to see. Another option would be to refer to the track colours in the text instead of letters.
We thank the reviewer for pointing out this oversight. Now we have removed the letters from Figure 1 and are referring to the track colours in the figure legend instead of the letters page 40 (lines 795-798)

Line 70: Should not use the word "theory" this way. These are hypotheses.
We thank the reviewer for pointing out this oversight. We have now replaced the word “theories” with “hypotheses” in line 55.

Line 79: Should be written at "subgenome". This applies throughout the manuscript.
We thank the reviewer for pointing out this oversight. We have now checked all appearances of the word “subgenome” in the manuscript and amended where necessary.

Lines 81-84: I think this needs to be reworded. I believe the intention is to say that genes that have a higher expression level are more likely to be retained if one of the paralogs is lost. If a copy is pseudogenized and a comparison of expression of the two copies is made, it must be the one that is not pseudogenized that has a higher expression level.
We thank the reviewer for this suggestion to improve the clarity of the manuscript. We have now re-written the sentencein lines 62-68.

Line 120: It would be helpful to also cite some literature demonstrating B. decipiens has n = 20 chromosomes (at least in some accessions). Assemblies are not necessarily indicative of chromosome number—especially when some Hi-C methods require a target chromosome number in the scaffolding process. Chheda, de Wet, and Harlan (1961) is one such publication.
https://www.tandfonline.com/doi/pdf/10.1080/00087114.1961.10796026
We thank the reviewer for this suggestion. We have now included two references depicting the haploid chromosome number of B. decipiens including the one suggested by the reviewer and reworded the sentence to improve clarity in lines 95-96.

Table 1: "Total number" needs to indicate what this is a total number. I assume scaffolds/contigs, but this should be stated.
We have added the word “scaffolds” after “Total number” in Table 1.

Line 127: RNA-Seq or transcriptome sequencing is the common way to write this. RNA-Seq (or RNASeq) is a well-known method, so I would think you can get by without the need to describe what RNA stands for in parentheses.
We have removed the description in parentheses and replaced the word as RNA-Seq data in line 103.

Line 134: Missing something after [37] to connect the portions of the sentence.
We have re-written that sentence more clearly in line 111.

Lines 143-150: There should be more information regarding the annotation of TEs in the genome. The use of a program like EDTA (https://genomebiology.biomedcentral.com/articles/10.1186/s13059-019-1905-y) is recommended. A table depicting the different superfamilies would be a good addition, but there should be at least percentages for the major superfamilies to better describe the TE diversity.
We thank the reviewers for this suggestion to include a detailed repeat annotation in the manuscript. We have included a table with a summary of the presence of the major repeat classes in the genome and also the superfamilies with the percentage of the genome covered by each superfamily presented (Table 3, page 9). Further we have included a new section in our methods "Detailed identification of repeat family diversity in the genome" to describe the use of the EDTA pipeline for the repeat annotations (lines 592-598).

Line 169: BLASTP is repeated.
We have now included parentheses where necessary to remove the appearance of the word BLASTP repeating in line 156.

Lines 212-220: It would be helpful if this was expanded for clarity. Is the claim here that there is homoeologous exchange with replacement or is there fractionation of the region post or with exchange?
We used the HMM to determine if there was evidence for exchange of chromosomal regions between the subgenomes. In particular we were interested in if there was evidence of reciprocal homoeologous exchange as this would not be evident in the synteny plots and we did not want to misassign genes to the wrong subgenome for downstream analysis. We provided a clearer description to further describe why we conducted the analysis line 191-202.

Line 305: Probably better to say "suggest that" instead of "revealed how".
We thank the reviewer for this suggestion. We have now amended the sentence as suggested in line 295.

Line 308: Providing the densities of transposons per subgenome, especially in regard to proximity to gene space would be very helpful in understanding the relative subgenome dominance/transposon interaction. Other studies have suggested that higher TE abundance/density near genes results in a locus (and cumulatively subgenome) being dominated. It is curious if that is the case here or not. More relative representation of some elements does not exactly tell us this.
We thank the reviewer for this interesting and thoughtful suggestion. In order to address this suggestion we have now calculated the TE density flanking genes using a software pipeline called TE density (Teresi et al., 2022). Further we tested if there is a significant difference between the TE density near genes in the two subgenomes using non-parametric tests (Mann-Whitney U test) and failed to find any significant difference, although it was in the predicted direction with lower densities near genes in the dominant subgenome. Similarly we tested for any significant differences in TE density near genes retained as single copy and duplicate genes and did not discover any difference. The results of these new tests can be found from lines 263-283 in the manuscript while the new methods are found from lines 727-741.

Line 319: This is the description that needs to be used in lines 81-84.
We thank the reviewer for this suggestion to improve the clarity of the manuscript, as mentioned above we have now reworded the sentence referred as "line 81-84" now in "lines 62-68".

Line 326: Use of "theory". It is a hypothesis.
Now we have replaced the word "theory" with the word "hypothesis".

Line 331: Just check all use of the word "theory".
Now we have replaced the word "theories" with the word "hypotheses" throughout the manuscript.

Line 361: "Subfunctionalization" is what I think you mean instead of "specialisation".
We thank the reviewer for pointing out this oversight. We have now replaced the word "specialisation" with the word "subfunctionalization" in line 348.

Line 368-369: "Andropogoneae"
We thank the reviewer for pointing out this oversight. As mentioned before, we have now checked the spellings of the word "Andropogoneae" all throughout the manuscript and have fixed any spelling errors where necessary.

Line 386: Some discussion on the previous suggestion of B. decipiens being a diploid species of the BCD clade is warranted. How does this compare to the paleopolyploidy of B. decipiens. Though it is a paleopolyploid, B. decipiens has maintained a lot of its genes in paralog copies making it very much a polyploid species. I am curious how this is reconciled with previous literature.
Distinguishing between diploids and (paleo)polyploids can be ambiguous in flower plant families, many of which have a long history of repeated genome duplication followed by gradual diploidization. The base chromosome number of the group is 10 and we found previous literature addressing B. decipiens as an old world polyploid (De Wet et al., 1963) based on chromosome counts as it is n=20. We identified that the parental species speciated approximately 6 million years ago followed by a hybridization event ~3 million years ago. This genome history is very similar to the situation in Miscanthus sinensis (Mitros et al., 2020) and in both cases the subgenomes are sufficiently divergent that they are genetic diploids. In support of this we were easily able to resolve the subgenomes in our assembly based on their unique genetic composition and our unpublished population genomic analysis is also consistent with disomic inheritance of markers. However, have now stopped referring to the species as diploid and instead refer to it as a paleotetraploid and a "genetic diploid" to avoid confusion.

Line 427: Where did these 24 populations come from? It is unclear what they are doing in the paper other than serving as other sources for genome sizing.
We thank the reviewer for the opportunity to clarify this statement. These 24 populations were collected from different places from Victoria and NSW in Australia. Individual samples from these populations were used to test for ploidy to identify diploid individuals of B. decipiens, as the plant is very similar morphologically to one of its' close relatives Bothriochloa macra which is a neopolyploid species. We wanted to identify the species with the smaller and less complex genome, B. decipiens, for the sequencing and the assembly of the reference genome. We have now included a sentence on where these populations come from in the manuscript in lines 412-414 and also a small description on the reason for using all these populations is given from lines 414-417.

Line 429: Numbers less than ten should be written out: five instead of 5.
We have now replaced "5" with the word "five" in line 417.

Line 433: Missing one of the standards.
We have mistakenly written that three standards were used instead of two. Now we have corrected that mistake in line 419..

Line 434: Were the standards added to each of the Bothriochloa samples? This is a common practice for flow cytometry in plants but not clear from the text.
Our FCM method was standardised so that all samples were disrupted for the same amount of time, with the same amount of force and with the same volume of buffer, greatly decreasing preparation variability. Additionally, because we were sampling unknown samples (in fact, we did not even know at the time if the samples we were measuring were diploid or polyploid), it would have been difficult to choose the correct standards to co-chop with because they could potentially overlap with our sample peaks. We did not directly add standards to the samples, but ran standards before and after runs to watch for shifts in

fluorescence. This is stated in the line 432-434.

Line 444: I do not think propidium iodide needs to be capitalised.
We have now written the word “propidium iodide” without the unnecessary capitalization in line 430.

Line 458: Is the haploid not 1.28 Gb?
The haploid size estimation via flow cytometry was 1.25 Gb not 1.28 Gb. Further the size of the final assembly was 1.21 Gb (Table 1).

Lines 458-459: Is this known or speculation? Should probably remove.
This was not speculation as the species are virtually identical except for the ploidy and the anther number, which is very difficult to identify in the field. Furthermore, we have done flow cytometry, genetic analysis and morphological identification on many of these same populations to show that the polyploid is indeed B. macra. We removed “likely” in line 444 to eliminate the possibility that another species was the likely cause of the ploidy variation.

Line 464: Is there some indication of how Dovetail did the isolations? At least a general technique if proprietary?
We assume the reviewer is referring to the DNA extraction process by “how Dovetail did the isolations”. We have included the method that Dovetail followed in order to extract DNA from the samples that were shipped in from line 449-456. Further to improve the clarity of this section we reworded the sentence as “The tissue was then shipped to Dovetail Genomics for the completion of DNA extractions by using the following steps.” in line 449.

Line 469: What is G2? Is there a more descriptive name for this?
The term “G2” refers to a buffer solution used in DNA/RNA extractions to enhance the yield of extracted DNA/RNA. In order to improve the clarity we have now written G2 DNA enhancer buffer solution instead of G2 in line 454..

Line 471: Usually said as "library construction" even if there is more than one.
We have now written “library construction” instead of “library constructions” in line 466.

Lines 520-525: What are the threshold parameters for breaks and joins?
We added more details in lines 495-502.

Line 542: What parameters were used for Trinity?
Trinity was run by specifying the sequence type (seqType) as fastq(fq) and specifying the two paired end reads using the —left and –right options. We did not use any other specific parameters while running Trinity. Now we have explained this in the text in line 520-522.

Line 552: How were the false positives identified?
Common feature of false positives is that the alignment between them (presumed “LTRs”), often extends beyond the boundary of the “LTR”. So we retrieved the flanking sequences of the LTRs, and used Blast to see if the flanking sequences were also alignable, and if so the putative LTR candidates were excluded. This was done using custom perl scripts provided in the instructions that we followed for advanced repeat library construction for the MakerP pipeline. We have now reworded the sentence on identifying false positives to enhance the clarity of the manuscript in lines 534-538.

Line 558: What parameters were used for RepeatModeler?
Here we used default parameters for running RepeatModeler. First a database was generated using the unmasked sequences that were not masked as repeats in the earlier steps using “BuildDatabase” command and the resulting database was the only input to run RepeatModeler in this step. We have now written that a database created out of the unmasked sequences was used as an input file to run RepeatModeler in this step in line 542.

Line 718: How was convergence in Tracer assured? What ESS values had to be reached?
We used Tracer v. 1.6 to visualise replicate analyses, and determined convergence when the joint density (posterior) of each replicate stabilised to a overlapping, stationary distribution. We continued analyses until the ESS reached at least 200. We have included this information in the manuscript in line 697-699.

Reviewer #3: (Remarks to the Author):

The manuscript "Genome assembly of an Australian native grass species reveals a recent whole genome duplication and biased gene retention of genes involved in stress response" assembled a genome of Bothriochloa decipiens with a total length of 1.2Gb and scaffold N50 of 42.637 Mb, and further found it is a diploidized allotetraploid by comparative analysis. The authors here mainly focused on the WGD event and sub-genome dominance, particularly in relation with stress responses (Duplicates were enriched for genes involved in transcription and response to external stimuli like drought) or improved adaptation of C4 species to the environment of Australia.

The data here, such as fig5 seemed not to support the point of stress responses very well.
We have now added Table 5 in results to support the point of stress response. Particularly in Table 5b we report a summary of the most significant GO terms (P-value ≤ 1 x 10-3) relating to transcription and external stress response among overrepresented genes in genes retained as duplicates as reported by the gene enrichment analysis. In this we report the terms: response to water deprivation, positive regulation of response to salt stress, negative regulation of abscisic acid signalling pathway, response to absence of light and response to cold. All these terms came up as significant GO terms (P-value ≤ 1 x 10-3) among genes retained as duplicates. Further we have now put Fig 5 in supplementary material as Figure S4 for further illustration of the point.

It is very interesting that Bothriochloa decipiens is a diploidized allotetraploid, instead of a diploid species thought originally. And the assembly of two subgenomes was a pretty good job without clear information of diploid ancestors. A question here is whether there is an even closer one other than sorghum.
We would like to acknowledge that there are closer diploid relatives than Sorghum to our study species B. decipiens, but Sorghum was the only closest diploid relative with a genome assembly and high quality genome annotations that was essential for the downstream comparative analyses conducted in this manuscript.

About the dominance of subgenome, since it might not be shared by all polyploids, any more evidence about how the stresses affected biased gene retention here in Bothriochloa decipiens?
We could not find published evidence showing how stresses have affected gene retention in B. decipiens. However in an unpublished population genomic analysis in B. decipiens individuals, loci that showed strong associations with environmental variation were more likely to be retained as duplicates in the genomes, lending further support to the common finding that loci involved in adaptation tend to be retained as duplicated. We note that we did not claim that the biassed gene retention among subgenomes is caused by stresses, but rather that genes retained as duplicated are more likely to be involved in adaptation. There could be equal loss of non-stress related genes between the subgenomes (no biassed fractionation) resulting in this pattern in gene function related to stress response in the retained duplicates. It would be interesting, however, to determine if there was an interaction between the parental genomes' stress response profile and genome dominance, but we do not have any expression data in this species to address this question.

What's the contig N50?
We have now included a new row of information presenting the contig N50 values of the genome assemblies in Table 1, the genome assembly statistic summary table.

Could the authors explain why not use 3rd generation sequencing methods such as Nanopore, SMRT, but 10X ?
The sequencing was done at a time when long read sequencing was more expensive and error prone. In

consultation with Dovetail, we decided that 10X was the best and most cost effective approach.

Line 38: Found evidence of biased fractionation and differences in the…, was subject missing here?

We corrected this typo.

There is a nice review about subgenome,"One subgenome to rule them all: underlying mechanisms of subgenome dominance", and it would be good to cite it here.
We have now cited this publication on subgenome dominance in lines 68,265 and 308.

Line 323: DNA methylation is known for its role in epigenetic gene silencing [52-54] and in 324 restricting TE activity. Wondering whether there were enough data here to justify the effects of DNA methylation of TE on biased gene retention/gene expression of subgenome?

We modified this section to simply point out the pattern and suggest further exploration (lines 312-318).

Close
