# Supplementary material for: Genome assembly of an Australian native grass species reveals a recent whole-genome duplication and biased gene retention of genes involved in stress response
Source: Gigascience. 2023 May 12;12:giad034. doi: 10.1093/gigascience/giad034 (PMC10176504; doi:10.1093/gigascience/giad034)
Supplement: giad034_Reviewer_2_Report_Original_Submission — Michael McKain -- 9/19/2022 Reviewed [file giad034_reviewer_2_report_original_submission.pdf]

Reviewer Report

Title: Genome assembly of an Australian native grass species reveals a recent whole genome duplication and biased gene retention of genes involved in stress response

Version: Original Submission **Date:** 9/19/2022

Reviewer name: Michael McKain

Reviewer Comments to Author:

De Silva et al. present a chromosome-scaffolded genome of Bothriochloa decipiens. The manscript is primary a genome report, focusing on gene content and pattern of gene retention post whole genome duplication. There are a few areas that the authors should consider in a revision to improve the genome annotation and subsequent analyses.
Issues to consider:
1) The transposon annotation and description could use more detail on the superfamilies that were identified. More diversity of superfamilies, especially those considered DNA elements, should be described. The methods for annotated the elements focused on LTRs and MITEs. There is no indication of annotation for elements like helitrons. I would recommend using software like EDTA since it is covers the majority of TE types.
2) The finding that the more dominant subgenome also had more TE activity is interesting. It would be useful for the authors to describe the location of TEs relative to gene space. Other studies have demonstrated that there is a link between TE density near genes and paralog loss post WGD.
Check spelling of "Andropogoneae" and "homoeologous" throughout.
Figure 1: I would create a key that uses the track colors to assign the letters. As they are placed, they are difficult to see. Another option would be to refer to the track colors in the text instead of letters.
Line 70: Should not use the word "theory" this way. These are hypotheses.
Line 79: Should be written at "subgenome". This applies throughout the manuscript.
Lines 81-84: I think this needs to be reworded. I believe the intention is to say that genes that have a higher expression level are more likely to be retained if one of the paralogs is lost. If a copy is pseudogenized and a comparison of expression of the two copies is made, it must be the one that is not pseudogenized that has a higher expression level.
Line 120: It would be helpful to also cite some literature demonstrating B. decipiens has n = 20 chromosomes (at least in some accessions). Assemblies are not necessarily indicative of chromosome numberâ€”especially when some Hi-C methods require a target chromosome number in the scaffolding process. Chheda, de Wet, and Harlan (1961) is one such publication.
https://www.tandfonline.com/doi/pdf/10.1080/00087114.1961.10796026
Table 1: "Total number" needs to indicate of what this is a total number. I assume scaffolds/contigs, but this should be stated.
Line 127: RNA-Seq or transcriptome sequencing is the common way to write this. RNA-Seq (or RNASeq) is a well-known method, so I would think you can get by without the need to describe what RNA stands for in parentheses.

Line 134: Missing something after [37] to connect the portions of the sentence.
Lines 143-150: There should be more information regarding the annotation of TEs in the genome. The use of a program like EDTA (https://genomebiology.biomedcentral.com/articles/10.1186/s13059-019-1905-y) is recommended. A table depicting the different superfamilies would be a good addition, but there should be at least percentages for the major superfamilies to better describe the TE diversity.
Line 169: BLASTP is repeated.
Lines 212-220: It would be helpful if this was expanded for clarity. Is the claim here that there is homoeologous exchange with replacement or is there fractionation of the region post or with exchange?
Line 305: Probably better to say "suggest that" instead of "revealed how".
Line 308: Providing the densities of transposons per subgenome, especially in regard to proximity to gene space would be very helpful in understanding the relative subgenome dominance/transposon interaction. Other studies have suggested that higher TE abundance/density near genes results in a locus (and cumulatively subgenome) being dominated. It is curious if that is the case here or not. More relative representation of some elements does not exactly tell us this.
Line 319: This is the description that needs to be used in lines 81-84.
Line 326: Use of "theory". It is a hypothesis.
Line 331: Just check all use of the word "theory".
Line 361: "Subfunctionalization" is what I think you mean instead of "specialization".
Line 368-369: "Andropogoneae"
Line 386: Some discussion on the previous suggestion of B. decipiens being a diploid species of the BCD clade is warranted. How does this compare to the paleopolyploidy of B. decipiens. Though it is a paleopolyploid, B. decipiens has maintained a lot of its genes in paralog copies making it very much a polyploid species. I am curious how this is reconciled with previous literature.
Line 427: Where did these 24 populations come from? It is unclear what they are doing in the paper other than serving as other sources for genome sizing.
Line 429: Numbers less than ten should be written out: five instead of 5.
Line 433: Missing one of the standards.
Line 434: Were the standards added to each of the Bothriochloa samples? This is a common practice for flow cytometry in plants but not clear from the text.
Line 444: I do not think propidium iodide needs to be capitalized.
Line 458: Is the haploid not 1.28 Gb?
Lines 458-459: Is this known or speculation? Should probably remove.
Line 464: Is there some indication of how Dovetail did the isolations? At least a general technique if proprietary?
Line 469: What is G2? Is there a more descriptive name for this?
Line 471: Usually said as "library construction" even if there is more than one.
Lines 520-525: What are the threshold parameters for breaks and joins?
Line 542: What parameters were used for Trinity?
Line 552: How were the false positives identified?
Line 558: What parameters were used for RepeatModeler?

Line 718: How was convergence in Tracer assured? What ESS values had to be reached?

Methods

Are the methods appropriate to the aims of the study, are they well described, and are necessary controls included? Choose an item.

Conclusions

Are the conclusions adequately supported by the data shown? Choose an item.

Reporting Standards

Does the manuscript adhere to the journal's guidelines on [minimum standards of reporting?]() Choose an item.

Choose an item.

Statistics

Are you able to assess all statistics in the manuscript, including the appropriateness of statistical tests used? Choose an item.

Quality of Written English

Please indicate the quality of language in the manuscript: Choose an item.

Declaration of Competing Interests

Please complete a declaration of competing interests, considering the following questions:

- Have you in the past five years received reimbursements, fees, funding, or salary from an organisation that may in any way gain or lose financially from the publication of this manuscript, either now or in the future?
- Do you hold any stocks or shares in an organisation that may in any way gain or lose financially from the publication of this manuscript, either now or in the future?
- Do you hold or are you currently applying for any patents relating to the content of the manuscript?
- Have you received reimbursements, fees, funding, or salary from an organization that holds or has applied for patents relating to the content of the manuscript?
- Do you have any other financial competing interests?
- Do you have any non-financial competing interests in relation to this paper?

If you can answer no to all of the above, write 'I declare that I have no competing interests' below. If your reply is yes to any, please give details below.

I declare that I have no competing interests.

I agree to the open peer review policy of the journal. I understand that my name will be included on my report to the authors and, if the manuscript is accepted for publication, my named report including any attachments I upload will be posted on the website along with the authors' responses. I agree for my report to be made available under an Open Access Creative Commons CC-BY license (http://creativecommons.org/licenses/by/4.0/). I understand that any comments which I do not wish to be included in my named report can be included as confidential comments to the editors, which will not be published.

Choose an item.

To further support our reviewers, we have joined with Publons, where you can gain additional credit to further highlight your hard work (see: https://publons.com/journal/530/gigascience). On publication of this paper, your review will be automatically added to Publons, you can then choose whether or not to claim your Publons credit. I understand this statement.

Yes Choose an item.
