# Supplementary material for: Genome assembly of an Australian native grass species reveals a recent whole-genome duplication and biased gene retention of genes involved in stress response
Source: Gigascience. 2023 May 12;12:giad034. doi: 10.1093/gigascience/giad034 (PMC10176504; doi:10.1093/gigascience/giad034)
Supplement: giad034_Reviewer_3_Report_Original_Submission — Kehua Wang -- 10/5/2022 Reviewed [file giad034_reviewer_3_report_original_submission.pdf]

Reviewer Report

Title: Genome assembly of an Australian native grass species reveals a recent whole genome duplication and biased gene retention of genes involved in stress response

Version: Original Submission **Date:** 10/5/2022

Reviewer name: Kehua Wang

Reviewer Comments to Author:

The manuscript "Genome assembly of an Australian native grass species reveals a recent whole genome duplication and biased gene retention of genes involved in stress response" assembled a genome of Bothriochloa decipiens with a total length of 1.2Gb and scaffold N50 of 42.637 Mb, and further found it is a diploidized allotetraploid by comparative analysis. The authors here mainly focused on the WGD event and sub-genome dominance, particularly in relation with stress responses (Duplicates were enriched for genes involved in transcription and response to external stimuli like drought) or improved adaptation of C4 species to the environmental of Australia. However, the data here, such as fig5 seemed not to support the point of stress responses very well. It is very interested that Bothriochloa decipiens is a diploidized allotetraploid, instead of a diploid species thought originally. And the assembly of two subgenomes was a pretty good job without clear information of diploid ancestors. A question here is whether there is an even closer one other than sorghum. About the dominance of subgenome, since it might not be shared by all polyploids, any more evidence about how the stresses affected biased gene retention here in Bothriochloa decipiens?
Several minor questions:
1) what's the contig N50?
2) Could the authors explain why not use 3rd generation sequencing methods such as Nanopore, SMRT, but 10X ?
3) line 38: Found evidence of biased fractionation and differences in the…, was a subject missing here?
4) There is a nice review about subgenome,"One subgenome to rule them all: underlying mechanisms of subgenome dominance", and it would be good to cite it here.
5) Line 323: DNA methylation is known for its role in epigenetic gene silencing [52-54] and in
324 restricting TE activity. Wondering whether there were enough data here to justify the effects of DNA methylation of TE on biased gene retention/gene expression of subgenome a?

Methods

Are the methods appropriate to the aims of the study, are they well described, and are necessary controls included? Choose an item.

Conclusions

Are the conclusions adequately supported by the data shown? Choose an item.

Reporting Standards

Does the manuscript adhere to the journal's guidelines on minimum standards of reporting? Choose an item.

Choose an item.

Statistics

Are you able to assess all statistics in the manuscript, including the appropriateness of statistical tests used? Choose an item.

Quality of Written English

Please indicate the quality of language in the manuscript: Choose an item.

Declaration of Competing Interests

Please complete a declaration of competing interests, considering the following questions:

- Have you in the past five years received reimbursements, fees, funding, or salary from an organisation that may in any way gain or lose financially from the publication of this manuscript, either now or in the future?
- Do you hold any stocks or shares in an organisation that may in any way gain or lose financially from the publication of this manuscript, either now or in the future?
- Do you hold or are you currently applying for any patents relating to the content of the manuscript?
- Have you received reimbursements, fees, funding, or salary from an organization that holds or has applied for patents relating to the content of the manuscript?
- Do you have any other financial competing interests?
- Do you have any non-financial competing interests in relation to this paper?

If you can answer no to all of the above, write 'I declare that I have no competing interests' below. If your reply is yes to any, please give details below.

I declare that I have no competing interests

I agree to the open peer review policy of the journal. I understand that my name will be included on my report to the authors and, if the manuscript is accepted for publication, my named report including any attachments I upload will be posted on the website along with the authors' responses. I agree for my report to be made available under an Open Access Creative Commons CC-BY license (http://creativecommons.org/licenses/by/4.0/). I understand that any comments which I do not wish to be included in my named report can be included as confidential comments to the editors, which will not be published.

Choose an item.

To further support our reviewers, we have joined with Publons, where you can gain additional credit to further highlight your hard work (see: https://publons.com/journal/530/gigascience). On publication of this paper, your review will be automatically added to Publons, you can then choose whether or not to claim your Publons credit. I understand this statement.

Yes Choose an item.
